# Author Correction: High monoclonal neutralization titers reduced breakthrough HIV-1 viral loads in the Antibody Mediated Prevention trials

**DOI:** 10.1038/s41467-024-46805-8

**Published:** 2024-03-22

**Authors:** Daniel B. Reeves, Bryan T. Mayer, Allan C. deCamp, Yunda Huang, Bo Zhang, Lindsay N. Carpp, Craig A. Magaret, Michal Juraska, Peter B. Gilbert, David C. Montefiori, Katharine J. Bar, E. Fabian Cardozo-Ojeda, Joshua T. Schiffer, Raabya Rossenkhan, Paul Edlefsen, Lynn Morris, Nonhlanhla N. Mkhize, Carolyn Williamson, James I. Mullins, Kelly E. Seaton, Georgia D. Tomaras, Philip Andrew, Nyaradzo Mgodi, Julie E. Ledgerwood, Myron S. Cohen, Lawrence Corey, Logashvari Naidoo, Catherine Orrell, Paul A. Goepfert, Martin Casapia, Magdalena E. Sobieszczyk, Shelly T. Karuna, Srilatha Edupuganti

**Affiliations:** 1 Vaccine and Infectious Disease Division, Fred Hutchinson Cancer Center, Seattle, WA USA; 2 Department of Global Health, University of Washington, Seattle, WA USA; 3 Public Health Sciences Division, Fred Hutchinson Cancer Center, Seattle, WA USA; 4 Department of Biostatistics, University of Washington, Seattle, WA USA; 5 Department of Surgery, Duke University Medical Center, Durham, NC USA; 6 Perelman School of Medicine, University of Pennsylvania, Philadelphia, PA USA; 7 Department of Medicine, University of Washington, Seattle, WA USA; 8 National Institute for Communicable Diseases, National Health Laboratory Service, Johannesburg, South Africa; 9 Antibody Immunity Research Unit, Faculty of Health Sciences, University of the Witwatersrand, Johannesburg, South Africa; 10 Centre for the AIDS Programme of Research in South Africa, University of KwaZulu-Natal, Durban, South Africa; 11 Division of Medical Virology, Faculty of Health Sciences, University of Cape Town and National Health Laboratory Service, Cape Town, South Africa; 12 Department of Microbiology, University of Washington, Seattle, WA USA; 13 Center for Human Systems Immunology, Duke University, Durham, NC USA; 14 Departments of Surgery, Immunology, and Molecular Genetics and Microbiology, Duke University, Durham, NC USA; 15 Family Health International, Durham, NC USA; 16 Clinical Trials Research Centre, University of Zimbabwe College of Health Sciences, Harare, Zimbabwe; 17 Vaccine Research Center, National Institute of Allergy and Infectious Diseases, National Institutes of Health, Bethesda, MD USA; 18 Institute for Global Health and Infectious Diseases, The University of North Carolina at Chapel Hill, Chapel Hill, NC USA; 19 Department of Laboratory Medicine and Pathology, University of Washington, Seattle, WA USA; 20 South African Medical Research Council, HPRU, Durban, South Africa; 21 Desmond Tutu HIV Centre, Institute of Infectious Disease and Molecular Medicine and Department of Medicine, University of Cape Town, Cape Town, South Africa; 22 Division of Infectious Diseases, Department of Medicine, University of Alabama at Birmingham, Birmingham, AL USA; 23 Facultad de Medicina Humana, Universidad Nacional de la Amazonia Peru, Iquitos, Peru; 24 Division of Infectious Diseases, Department of Medicine, Vagelos College of Physicians and Surgeons, New York-Presbyterian/Columbia University Irving Medical Center, New York, NY USA; 25 GreenLight Biosciences, Medford, MA USA; 26 Division of Infectious Diseases, Department of Medicine, Emory University School of Medicine, Atlanta, GA USA

**Keywords:** HIV infections, Applied mathematics, Scientific data, Antibodies

Correction to: *Nature Communications* 10.1038/s41467-023-43384-y, published online 14 December 2023

The originally published version of this Article contained several errors in references to previous work.

In the Introduction, references 23 and 26 were inadvertently omitted in two sentences, and have been added as follows: ‘The Antibody Mediated Prevention (AMP) trials were the first human efficacy studies of infused bnAbs for HIV-1 prevention (HVTN 704/HPTN 085 and HVTN 703/HPTN 081)^23^. The efficacy of a CD4-binding site bnAb, VRC01, was tested in men, women and transgender individuals who were vulnerable to HIV-1 in the Americas, sub-Saharan Africa and Europe^24–26^.’

The published Article incorrectly referenced Cale, E. M. et al. Neutralizing antibody VRC01 failed to select for HIV-1 mutations upon viral rebound. J Clin Invest 130, 3299–3304, 10.1172/JCI134395 (2020) in a sentence in the Discussion. The correct sentence instead cites reference 39 as follows: ‘The TZM-bl target cell assay used to quantify in vitro neutralization uses cells that are highly permissive to infection^39^, potentially allowing for larger reductions by VRC01.’

The published Article incorrectly referenced Gaebler, C. et al. Sequence Evaluation and Comparative Analysis of Novel Assays for Intact Proviral HIV-1 DNA. J Virol 95, 10.1128/JVI.01986-20 (2021) in a sentence in the Discussion. Gaebler et al. (2021) has been removed from the published Article. The correct sentence instead cites reference 64 as follows: ‘Moreover, the pseudoviruses used in the assay may be more VRC01 sensitive than wild-type viruses^64^.’

Due to a formatting error of the bibliography, several references were omitted in the published Article. These have been added as follows:

Reference 31 in the Introduction: ‘Viral load has long been associated with HIV-1 pathogenesis and progression^31^.’

Reference 36 in the Introduction: ‘Previously, breakthrough infections among some PrEP users appear to admit lower viral loads^36^.’

References 53–56 in the Discussion: ‘Blunting or delaying HIV-1 viremia might be relevant for pathogenesis^52^, reservoir creation^51^, viral evolution^53^, and/or onward transmission during acute infection. Furthermore, small but exciting studies^54,55^ showed other bnAbs can achieve prolonged (up to 2 years) HIV-1 suppression after stopping ART potentially due to a vaccinal effect in which infused bnAbs interact with virus, e.g., forming immune complexes, to enhance immune control^56^.’

References 67–69 in the Discussion: ‘Another explanation may be that cell-to-cell transmission of HIV-1 effectively lowers the ability of VRC01 to find virus to neutralize^67–69^.’

Reference 36 in the Discussion: ‘Viral load blunting and delay was also observed in breakthrough acquisition during ART-mediated prevention trials^36^.’

References 73–75 in the Discussion: ‘In contrast to ART monotherapy, which rapidly and deterministically leads to viral escape^73–75^, we show some evidence that bnAb monotherapy is relatively robust.’

References 56, 66 and 79 in the Discussion: ‘Our viral load model does not notably consider cell-to-cell transmission, any enhancement of CD8 T cell killing via VRC01, or any active mechanism whereby VRC01 kills infected cells (such as antibody dependent cellular cytotoxicity or ADCC) and/or mediates phagocytosis^66,79^. This would be particularly important to know given the hypothesized bnAb vaccinal effect mentioned above^56^ though it may be less critical here as VRC01 has demonstrated little effector functionality in prior studies^66^.’

This has been corrected in the PDF and HTML versions of the Article.

