# Supplementary material for: High monoclonal neutralization titers reduced breakthrough HIV-1 viral loads in the Antibody Mediated Prevention trials
Source: Nat Commun. 2023 Dec 14;14:8299. doi: 10.1038/s41467-023-43384-y (PMC10721814; doi:10.1038/s41467-023-43384-y)
Supplement: Supplementary file 1 — Supplementary Information [file 41467_2023_43384_MOESM1_ESM.pdf]

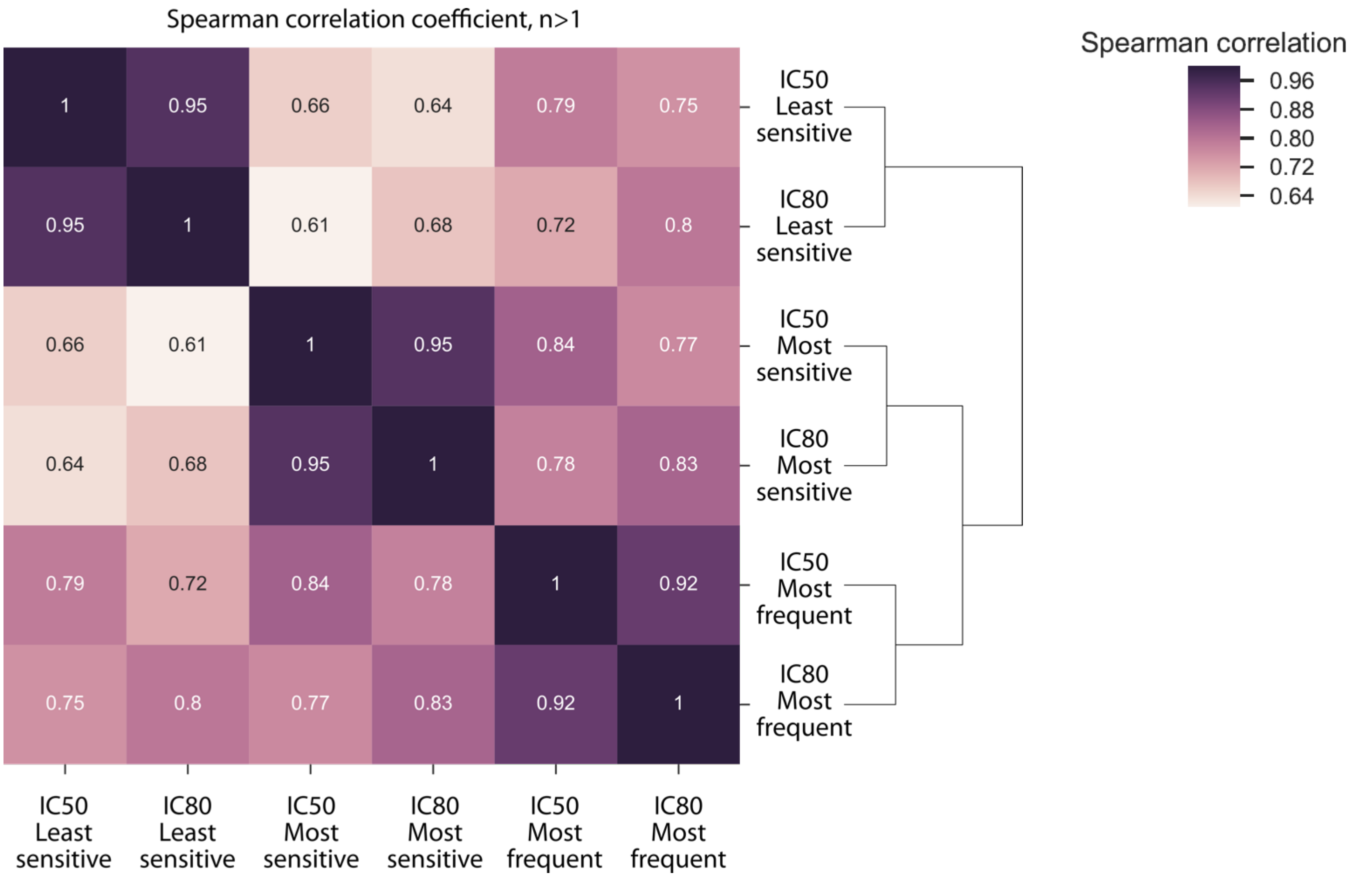

Supplementary Fig. 1. Correlation of sensitivity within host. Spearman correlation coefficient values for 6 acquired virus sensitivity measures for the 64 participants from whom more than 1 viral isolate was identified. Values are geometric means across experiments.

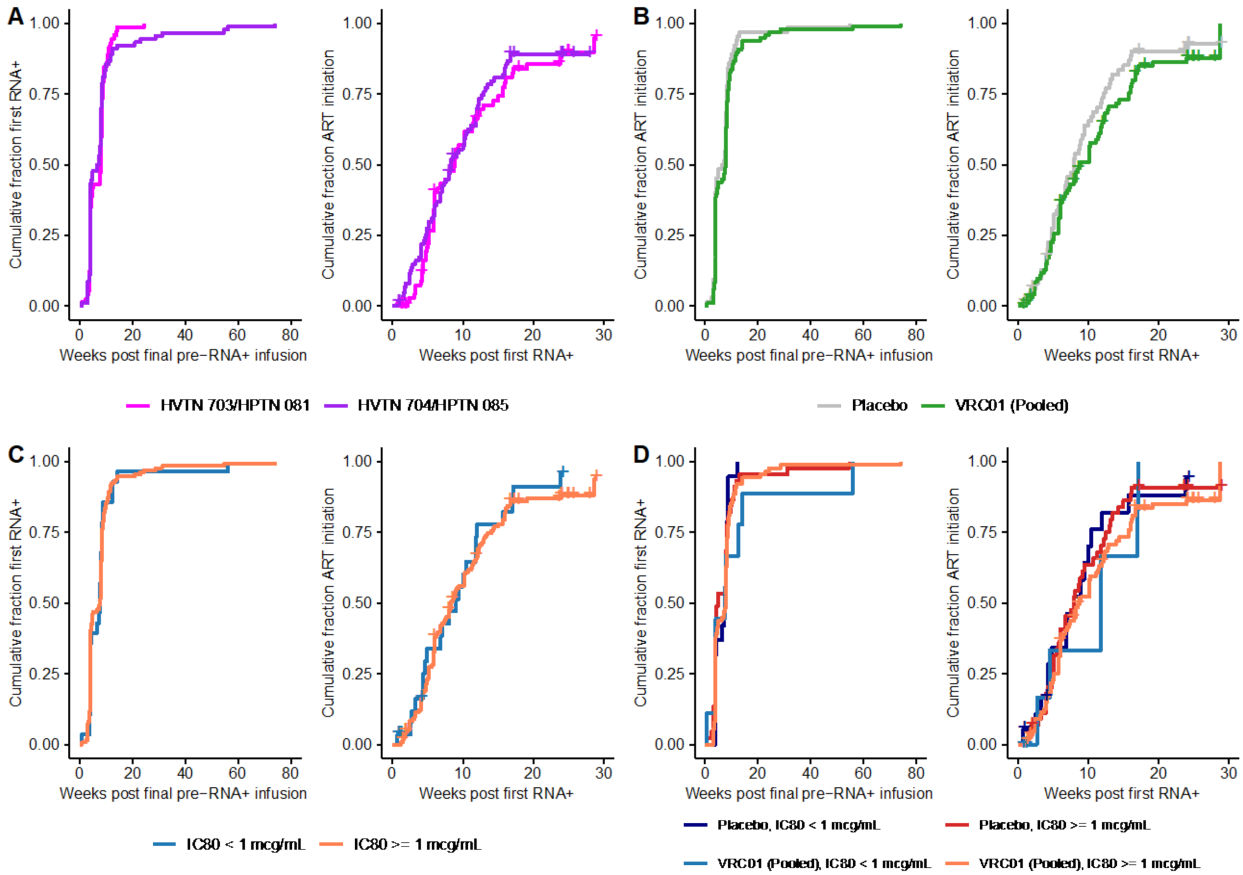

Supplementary Fig. 2. Cumulative incidence curves by groups for times between last negative, first positive, and ART initiation. The cumulative fraction of participants with a first RNA positive measurement by weeks post final pre-RNA+ infusion (left within each panel) and cumulative fraction of participants who had initiated ART by weeks post first RNA positive measurement (right within each panel) stratified by (A) trial, (B) treatment assignment, (C) VRC01 sensitivity category of acquired isolate with least sensitive IC80, and (D) the combination of treatment assignment and VRC01 sensitivity. Incidence curves were compared between strata using log-rank tests with all unadjusted p-values > 0.1. Statistics were calculated on the following numbers of participants: A) HVTN 703/HPTN 081 N=72, HVTN 704/HPTN 085 N=90; B) Placebo N=64, VRC01 Pooled = 98; C) IC80 < 1 μg/ml = 28, IC80 ≥ 1 μg/ml = 134, D) Placebo, IC80 < 1 μg/ml = 19; Placebo, IC80 ≥ 1 μg/ml = 45, VRC01 Pooled, IC80 < 1 μg/ml = 9; VRC01 Pooled, IC80 ≥ 1 μg/ml = 89.

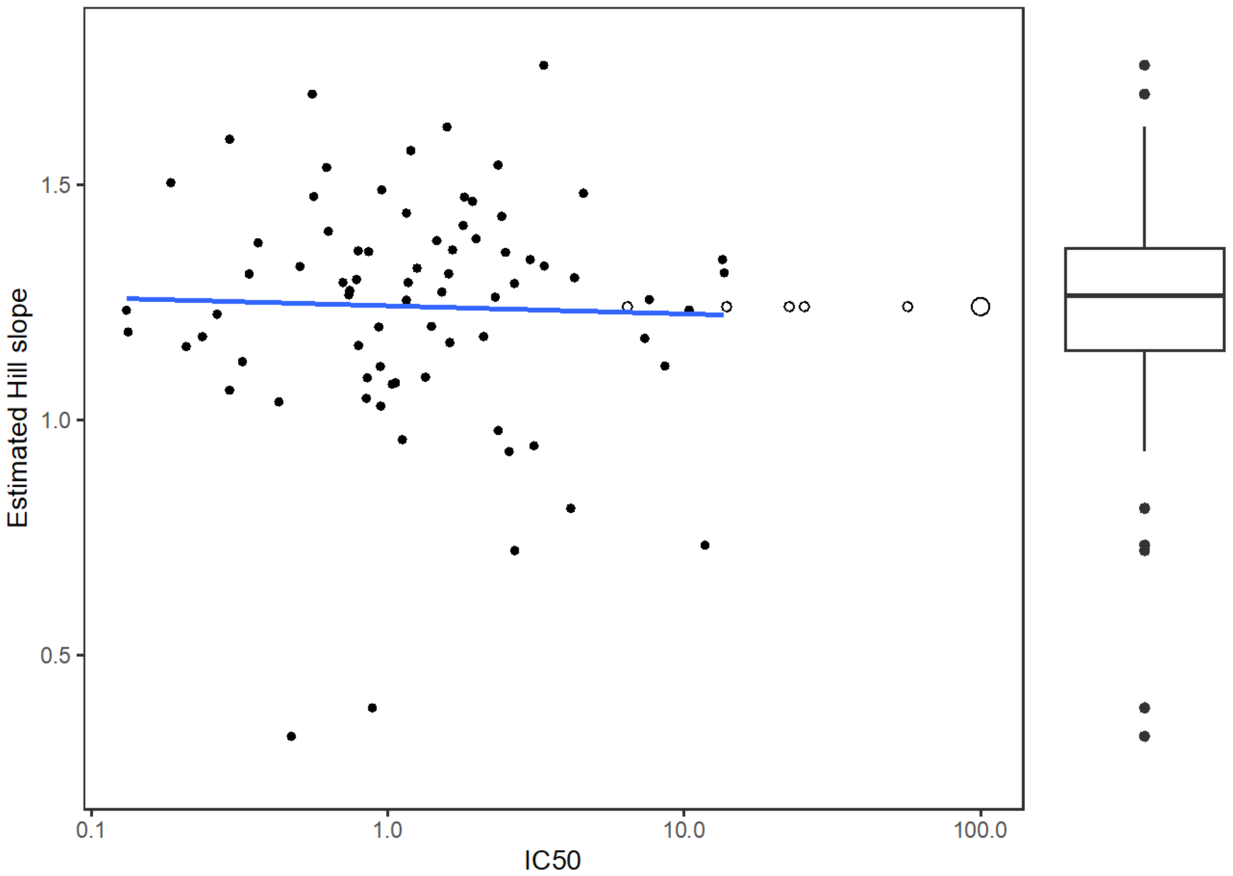

Supplementary Fig. 3. Estimated Hill slope for AMP viruses. The Hill slope was calculated from least sensitive viral IC50 and IC80 values using equation (3): h=-ln(4)/ln(IC50/IC80). Box plot indicates median ~1.3 with IQR ~1.1-1.4. Regression line depicts predicted mean Hill slope given IC50 values among sensitive viruses without IC80 censoring (IC50 < 100 and IC80 < 100). The 95% confidence interval of the predicted mean is depicted as the shaded region and the distribution of Hill slopes are depicted as a box plot on the right. As the regression line exhibited no trend, the overall mean of the Hill slope was used to impute Hill slopes for sensitive viruses with censored IC80 (IC50 < 100 and IC80 > 100, open circles) and resistant viruses (IC50 > 100, large open circle).

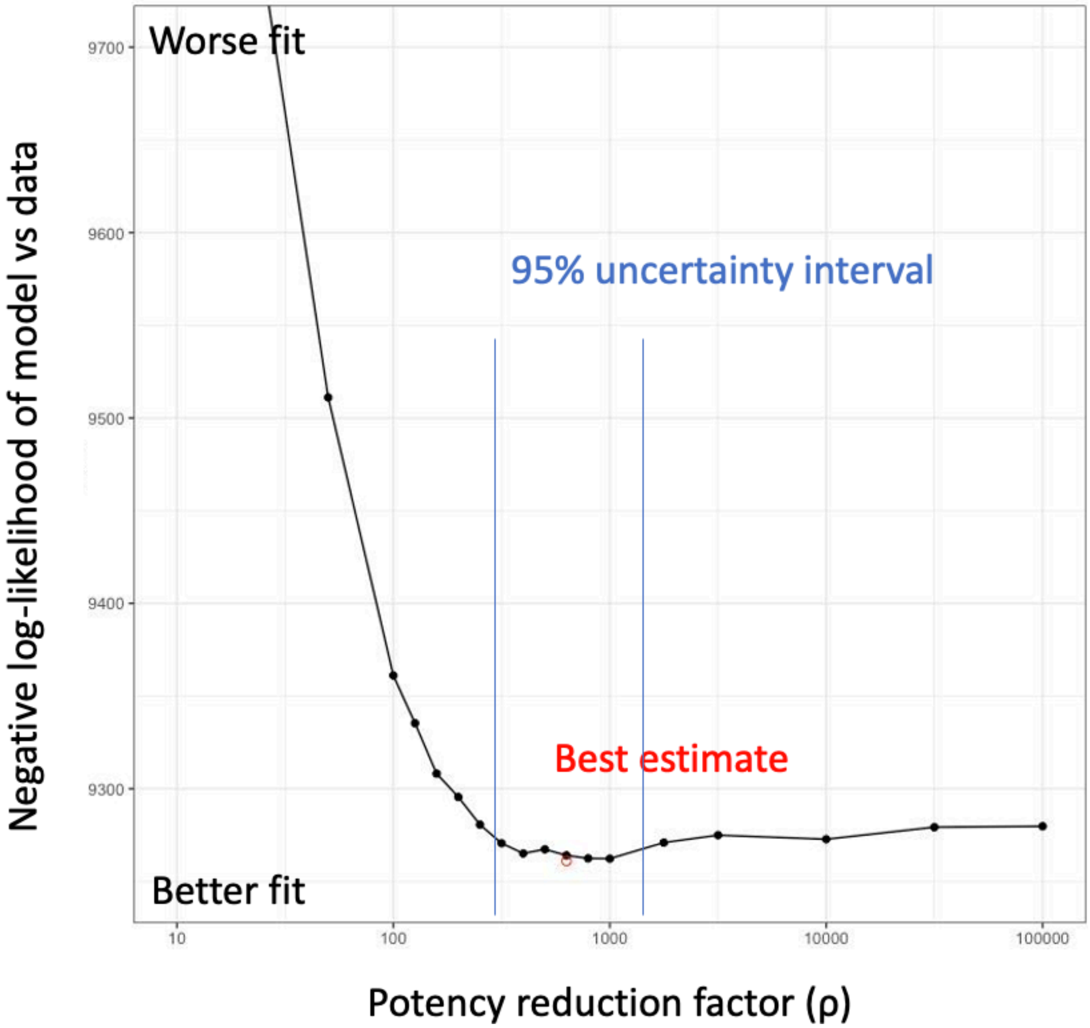

Supplementary Fig. 4. Estimation of potency reduction factor. The computed negative log-likelihood for varying values of the potency reduction factor, illustrating the best value having the highest likelihood and the uncertainty interval.

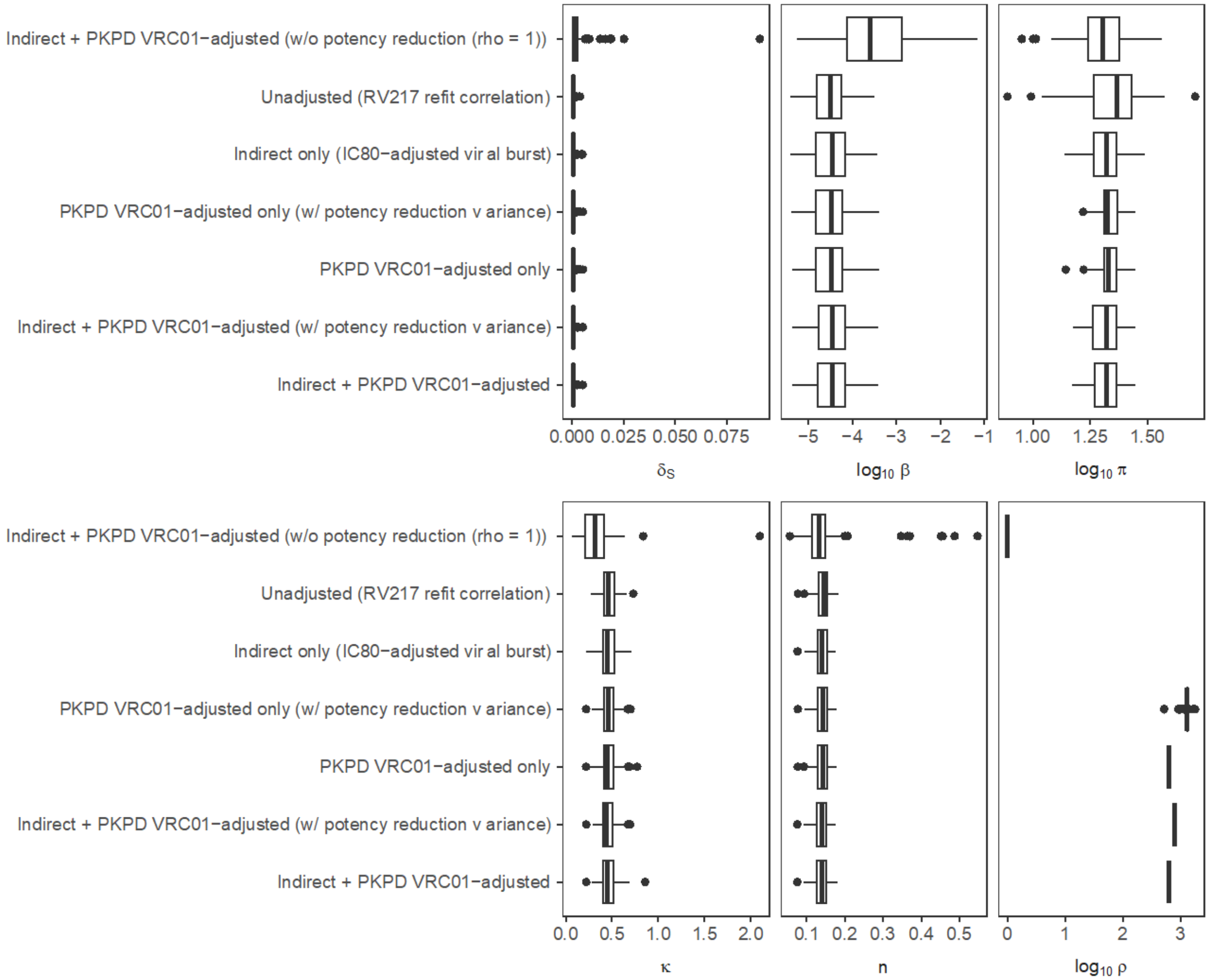

Supplementary Fig. 5. Parameter estimates across models. Values of estimated parameters are relatively constant across models. The notable difference is when potency reduction factor ρ is set to 1, infectivity ($\log_{10}\beta$) is forced to be much higher to account for the observed viral loads despite high titers of VRC01. Box plots indicate median, IQR (box) and 1.5x IQR (fliers).

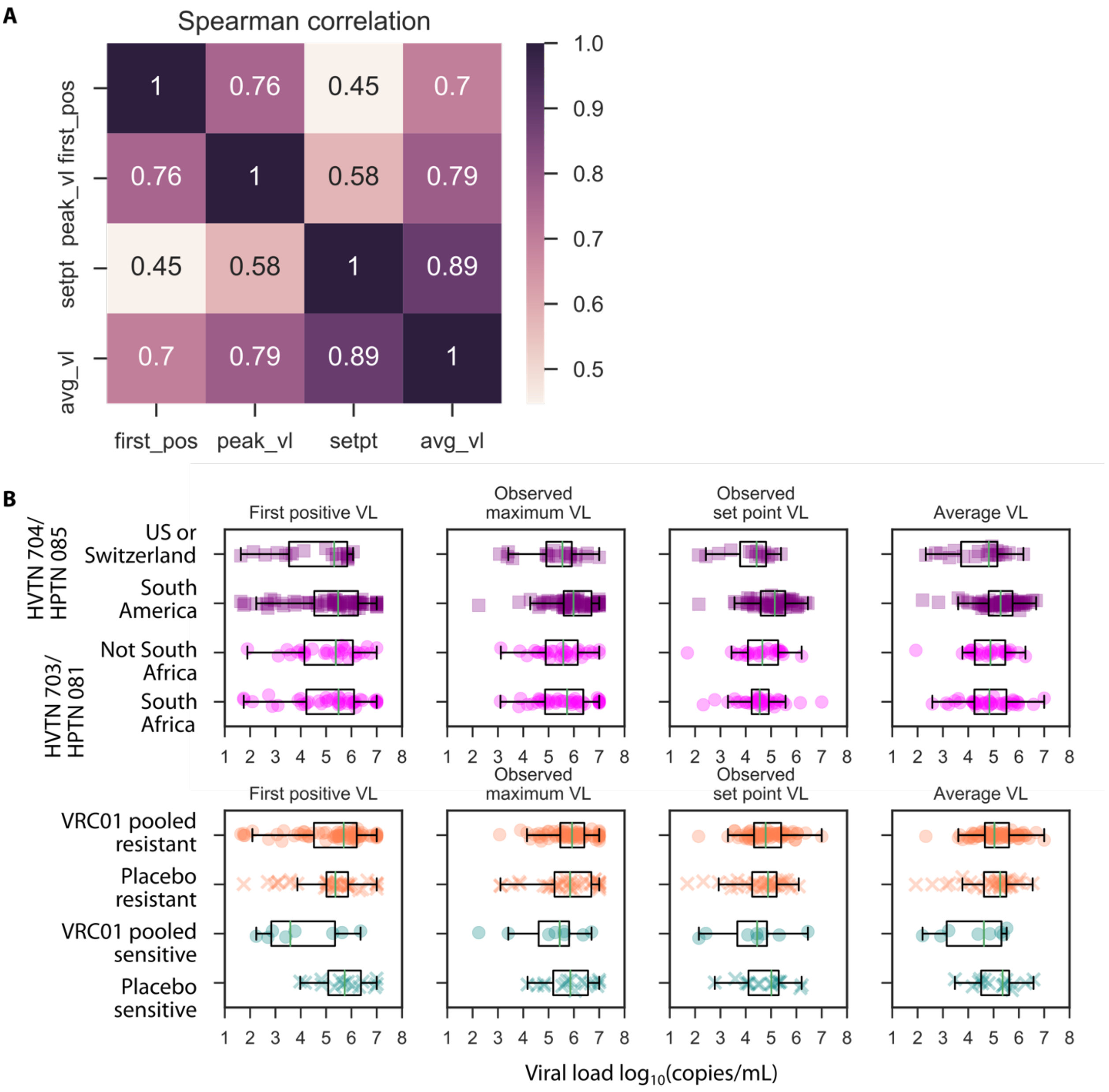

Supplementary Fig. 6. Observed viral dynamic metrics. A) Spearman correlation coefficient between observed viral load metrics. B) All comparisons of 4 observed viral load metrics by both geographic region (top, colors indicate trials) and VRC01 treatment/sensitivity groups (bottom, colors indicate sensitive vs resistant). Box plots indicate median, IQR (box) and 1.5x IQR (fliers).

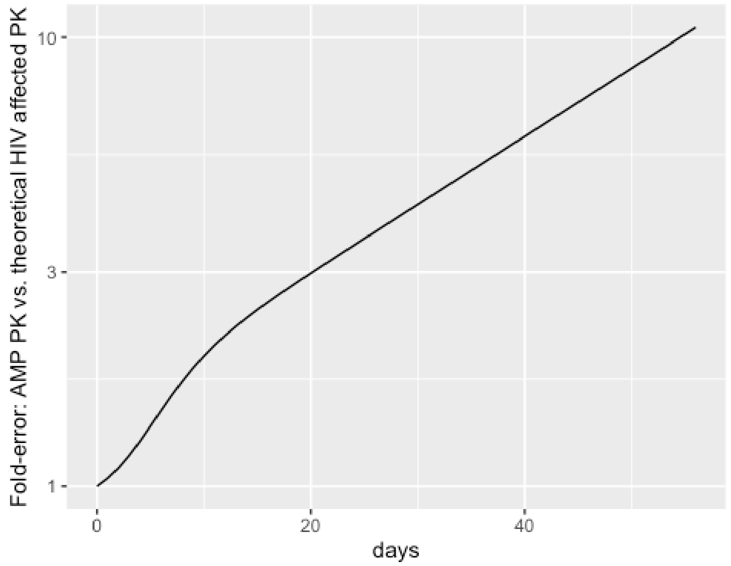

Supplementary Fig. 7. Simulating PK with a faster terminal half-life in people who acquire HIV. Based on data suggesting the bnAb PGDM1400 cleared 1.7x faster in PWH vs HIV-negative individuals, we simulated how much lower the VRC01 levels would be throughout dosing intervals if they followed the same decrease.

Supplementary Table 1. Numbers of HIV acquisitions during the AMP trial.
Data are grouped by treatment arm, acquired isolate information, and numbers of available longitudinal viral load measurements*.

	**Placebo (saline) (N=64)**	**VRC01 (10 mg/kg) (N=54)**	**VRC01 (30 mg/kg) (N=44)**	**Overall (N=162)**
**Region/Protocol**				
HVTN 703/HPTN 081				
South Africa	16 (25.0%)	15 (27.8%)	10 (22.7%)	41 (25.3%)
Not South Africa	13 (20.3%)	11 (20.4%)	7 (15.9%)	31 (19.1%)
HVTN 704/HPTN 085				
US/Switzerland	9 (14.1%)	7 (13.0%)	6 (13.6%)	22 (13.6%)
South America	26 (40.6%)	21 (38.9%)	21 (47.7%)	68 (42.0%)
**Synthesized isolates**				
1	38 (59.4%)	36 (66.7%)	24 (54.5%)	98 (60.5%)
2	18 (28.1%)	12 (22.2%)	14 (31.8%)	44 (27.2%)
3	6 (9.4%)	4 (7.4%)	5 (11.4%)	15 (9.3%)
4	2 (3.1%)	2 (3.7%)	1 (2.3%)	5 (3.1%)
**Sensitivity (IC80^)**				
< 1 μg/mL (sensitive)¶	19 (29.7%)	4 (7.4%)	5 (11.4%)	28 (17.3%)
≥ 1 μg/mL (resistant)¶	45 (70.3%)	50 (92.6%)	39 (88.6%)	134 (82.7%)
**Longitudinal VL observations#**				
1	2 (3.1%)	1 (1.9%)	1 (2.3%)	4 (2.5%)
2	17 (26.6%)	10 (18.5%)	14 (31.8%)	41 (25.3%)
3	13 (20.3%)	18 (33.3%)	5 (11.4%)	36 (22.2%)
4	11 (17.2%)	10 (18.5%)	8 (18.2%)	29 (17.9%)
> 4	21 (32.8%)	15 (27.8%)	16 (36.4%)	52 (32.1%)

ART, antiretroviral therapy; IC80, 80% inhibitory concentration; VL, viral load.

*There were 174 primary HIV-1 endpoints by Week 80 in the AMP trials. Of these 174, 12 were missing IC80 & IC50 data: 8 in HVTN 704/HPTN 085 and 4 in HVTN 703/HPTN 081. This table restricts to the 162 endpoints for which IC80/IC50 data were available.
^Sensitivity is reported for VRC01 against the least sensitive HIV-1 isolate if more than one isolate was synthesized.
¶ We use a predefined threshold from the Statistical Analysis Plan of the AMP trials to define sensitive vs resistant.
#VL observations are defined as HIV-1 RNA PCR > 20 copies/ml. All measurements occur prior to ART initiation.

Supplementary Table 2. AMP participants initiating ART after acquisition.

Numbers and percentages of participants in each treatment assignment group, pooled across both trials, who initiated ART at any time of the study (viral load measurements made after this time were not used in the analysis).

**Any ART initiation during study**	**Placebo (saline) (N=64)**	**VRC01 (10mg/kg) (N=54)**	**VRC01 (30mg/kg) (N=44)**	**Overall (N=162)**
No	8 (12.5%)	11 (20.4%)	8 (18.2%)	27 (16.7%)
Yes	56 (87.5%)	43 (79.6%)	36 (81.8%)	135 (83.3%)

Supplementary Table 3. Infusion timing vs diagnosis.
Numbers of participants receiving infusions relative to diagnosis and first positive detection. Participants in the placebo group received infusions of saline.

**Study Group**		**Overall**	**Infusion at/after diagnosis**	**Infusion at/after first positive**
Placebo	IC80 < 1	19	11	12
	IC80 ≥ 1	45	18	21
VRC01 (Pooled)	IC80 < 1	9	3	4
	IC80 ≥ 1	89	49	56*
Total	-	162	81	93

* One participant received two infusions following at/after first positive visit.

Supplementary Table 4. Population pharmacokinetic parameter estimates for AMP study participant VRC01 levels.

Abbreviations: %RSE: relative standard error of the fixed effect estimate, calculated as 100* the ratio of the standard error to the estimated value; CV: coefficient of variation for random effect estimates, calculated as 100x the variance (stddev^2) of the random effects distribution; R: Correlation coefficient between random effect estimates.

	Parameter	Estimate	%RSE	95% CI LBOUND	95% CI UBOUND	%CV
**Fixed effects**	CL (L/day): clearance from the central compartment	0.575	2.06	0.552	0.598	–
	Body weight influence on CL	0.331	21.39	0.193	0.470	–
	Study influence on CL	0.111	24.99	0.057	0.165	–
	Vc (L): volume of the central compartment	3.532	4.46	3.223	3.840	–
	Q (L/day): inter-compartmental distribution clearance	0.676	1.93	0.650	0.701	–
	Vp (L): volume of the peripheral compartment	4.749	2.12	4.552	4.946	–
	Study influence on Vp	0.165	16.41	0.112	0.218	–
**Standard Deviation of the Random Effects**	CL (L/day): clearance from the central compartment	0.172	5.62	0.153	0.191	17.19%
	Vc (L): volume of the central compartment	0.248	14.80	0.176	0.320	24.77%
	Q (L/day): inter-compartmental distribution clearance	0.064	19.56	0.039	0.088	6.38%
	Vp (L): volume of the peripheral compartment	0.145	7.97	0.123	0.168	14.55%
**Error Model Parameters**	Residual error (proportional)	0.188	1.48	0.183	0.194	18.81%

Supplementary Table 5. Estimated viral load model parameters.
For four models, we provide the value of each estimated model parameter θ, the standard deviation on that parameter (ω_θ), and correlations between parameters $r(\theta_1, \theta_2)$. Relative standard errors of model estimates are provided in parentheses (RSE %). Values determined to be optimal are shown in red. These values are carried forward left to right for the following models and in some cases, values are refit (red in later columns). "Previous RV217" indicates our previously published mathematical modeling reported in Reeves et al. J Roy Soc Interface 2021. "Present RV217" indicates refitting that model with additional correlation parameters which adjusted some timing estimates (t_0, defined relative to first positive) slightly. "Indirect effects" indicates fitting the Present RV217 model to placebo group data from the AMP study including a covariate (b) between viral burst and sensitivity. Note throughout β and π were Log10-transformed and normally distributed on the log scale.

Definitions and units for estimated mechanistic model [equation (5)] parameters are as follows: Estimated day of infection relative to first positive t_0 [days], viral clearance rate γ [day^{-1}], susceptible cell death rate δ_S [day^{-1}], viral infectivity β [μL virus^{-1} day^{-1}], viral production rate π [virus cell^{-1} day^{-}], infected cell removal rate κ [(cell/μL)$^{-n}$ day^{-1}] – note the unit depends on the value of the power law term, Holte-Cardozo power law density dependent infected cell death exponent n [unitless]. For the population nonlinear mixed effects model, the data noise was also estimated as additive to log10 viral load such that σ_V is the standard deviation of the data with units of log10 copies/mL. The indirect fitness cost term b_π adjusts the population estimated viral production rate based on the acquired virus IC80 centered on the median value [equation (6)] so it carries the same units as π [virus cell^{-1} day^{-}]. Each value for standard deviation (subscripted ω_θ) carries the same units as its original parameter. Each value of correlation between parameters $r(x, y)$ is unitless.

Models:	Previous RV217	Present RV217	Indirect effects	Indirect + direct effects + potency reduction
**Parameter (see legend for definitions and units)**	**Estimated value**	**(% RSE of estimate)**		
t_0	16.58	16.46 (14.10)		
α_S	38.26			
γ	23			
$\delta_S \times 10^4$	6.77			
$\log_{10}\beta$	-4.33			
$\log_{10}\pi$	1.32			
κ	0.43			
n	0.14			
ω_{t_0}	0.31	0.38 (28.72)		
ω_{δ_S}	1.37			
ω_β	0.61			
ω_κ	0.39			
ω_n	0.26			

$r(\delta_S, \delta_I)$	-1	-0.77 (18.92)		
$r(\log_{10}\beta, \kappa)$	-0.86	-0.71 (29.50)		
$r(\log_{10}\pi, \kappa)$	-0.82	0.18 (539.78)		
$r(\log_{10}\beta, \delta_S)$	0.89	0.91 (21.11)		
$r(\log_{10}\pi, \delta_S)$	0.86	-0.22 (395.19)		
$r(\log_{10}\pi, \log_{10}\beta)$	0.83	-0.41 (61.05)		
σ_V	1.16			
b_π			-0.07 (40.54)	
ω_π	0.26		0.14 (17.79)	
$\log_{10}\rho$				2.80 (5.52)

Supplementary Table 6. Model selection analysis results.
BICc = Corrected Bayesian Information Criterion; LogLik = Log-likelihood. Optimal models (lowest BICc) for placebo and VRC01-pooled participants are denoted by red.

Cohort	Virus-adjusted	Model Description	Analysis Model	BICc	LogLik
Placebo	Fixed RV217	(Reeves et al., RSIF 2021)	TRUE	6073	6063
Placebo	Unadjusted	Refit RV217 Variance	FALSE	6065	6034
Placebo	Unadjusted	Refit RV217 Correlation	TRUE	6012	5977
Placebo	Unadjusted	Refit RV217 Variance/Correlation	FALSE	6008	5965
Placebo	Adjusted	Cat. IC80-adjusted Holte power	FALSE	5989	5975
Placebo	Adjusted	IC80-adjusted infected cell death	FALSE	5987	5979
Placebo	Adjusted	Cat. IC80-adjusted infectivity	FALSE	5986	5972
Placebo	Adjusted	Cat. IC80-adjusted viral burst	FALSE	5986	5972
Placebo	Adjusted	IC80-adjusted Holte power	FALSE	5985	5977
Placebo	Adjusted	Cat. IC80-adjusted infected cell death	FALSE	5985	5971
Placebo	Adjusted	IC80-adjusted infectivity	FALSE	5976	5968
Placebo	Adjusted	IC80-adjusted viral burst	TRUE	5975	5966
VRC01	PKPD	Unadjusted (RV217 refit correlation)	FALSE	9368	9368
VRC01	PKPD	Indirect only (IC80-adjusted viral burst)	FALSE	9367	9367
VRC01	PKPD	PKPD VRC01-adjusted only (w/ potency reduction variance)	FALSE	9284	9273
VRC01	PKPD	PKPD VRC01-adjusted only	FALSE	9276	9270
VRC01	PKPD	Indirect + PKPD VRC01-adjusted (no potency reduction, ρ = 1)	FALSE	12533	12533
VRC01	PKPD	Indirect + PKPD VRC01-adjusted (w/ potency reduction variance)	FALSE	9276	9265
VRC01	PKPD	Indirect + PKPD VRC01-adjusted	TRUE	9267	9261

Supplementary Table 7. Numbers of observed viral load measurements.

The number of post-acquisition, pre-ART, viral load measurements among the 162 participants who acquired HIV by Week 80 after the first AMP study infusion and for whom IC80/IC50 data were available for VRC01 potency against the acquired isolates. Numbers are given overall as well as for each study arm and for sensitive and resistant viruses. Days indicate the range relative to diagnosis where [is inclusive.

	Placebo		VRC01 (10mg/kg)		VRC01 (30mg/kg)		Overall	
	**IC80 < 1 μg/mL (n=65)**	**IC80 ≥ 1 μg/mL (n=172)**	**IC80 < 1 μg/mL (n=15)**	**IC80 ≥ 1 μg/mL (n=185)**	**IC80 < 1 μg/mL (n=12)**	**IC80 ≥ 1 μg/mL (n=159)**	**IC80 < 1 μg/mL (n=92)**	**IC80 ≥ 1 μg/mL (n=516)**
**Days**								
[-125,0)	2	7	1	8	2	12	5	27
[0,1)	18	45	4	50	5	38	27	133
[1,22)	14	44	3	44	5	38	22	126
[22,38)	11	26	2	30	0	28	13	84
[38,52)	10	22	1	19	0	15	11	56
[52,70)	7	16	2	16	0	16	9	48
[70,140)	2	9	2	13	0	9	4	31
[140,197)	1	3	0	5	0	3	1	11
[197,400]	0	0	0	0	0	0	0	0

Supplementary Table 8. Post-acquisition positive viral load measurements.
Viral load measurements by study visit (v.x and v.y encode first positive viral load and confirmatory positive viral load measurements).

Visit Number	Total Positive*	Total	Scheduled Day	Days Relative to Diagnosis mean (range)	Days Relative to First positive mean (range)
Retrospective^	31	32	NA	-34.8 (-125, -5)	0.9 (0, 29)
v.x	160	160	Diagnosis	0 (0, 0)	6.1 (0, 125)
v.y	145	148	D14	13.2 (2, 63)	18.9 (2, 140)
31	67	67	D28	27.9 (24, 32)	34.4 (24, 84)
32	63	63	D42	42.2 (38, 46)	50.9 (38, 143)
33	47	47	D56	56 (52, 60)	67.2 (52, 181)
34	29	29	D84	85.6 (77, 98)	90.4 (77, 119)
35	11	11	D168	167.4 (140, 180)	176.2 (166, 203)
Off-schedule	37	51		44.5 (4, 175)	54.2 (5, 175)
Total	590	608	-	-	-

* Positive VL measurement defined as HIV-1 RNA PCR above the lower limit of quantification.
^ Measurements were retrospectively made on samples collected prior to first positive, some were indeed already positive.
