# Supplementary material for: High monoclonal neutralization titers reduced breakthrough HIV-1 viral loads in the Antibody Mediated Prevention trials
Source: Nat Commun. 2023 Dec 14;14:8299. doi: 10.1038/s41467-023-43384-y (PMC10721814; doi:10.1038/s41467-023-43384-y)
Supplement: Supplementary file 3 — Description of Additional Supplementary Files [file 41467_2023_43384_MOESM3_ESM.pdf]

Description of Additional Supplementary Files

File Name: Supplementary Data 1
Description: **All individual data and model fits for PK and viral loads in the VRC01 arms.** IC80(ls) at the top of each plot denotes the VRC01 IC80 for the least sensitive acquired isolate for that participant. Dots represent observed concentrations and viral loads, solid lines represent model output, and dashed lines connect observed values. PK curve colors indicate treatment arm (light blue: 10 mg/kg; green: 30 mg/kg). Viral load curve colors indicate acquired virus sensitivity to VRC01 (blue: VRC01 sensitive; orange: VRC01 resistant).

File Name: Supplementary Data 2
Description: **All individual data and model fits for PK and viral loads in the placebo arm.** Viral load curve colors indicate acquired virus sensitivity to VRC01 (blue: VRC01 sensitive; orange: VRC01 resistant). Dots represent observed viral loads, solid lines represent model output, and dashed lines connect observed values.

File Name: Supplementary Data 3
Description: **Complete model values for PK modeling to project VRC01 concentration and viral dynamic model parameters for each individual from the final viral load model.** Data shown include average and peak viral load for each participant, as well as viral load set point. Model parameters in column headings are named by their usage in the Monolix code, all are "mode" of posterior estimates, initT_mode is acquisition time relative to first positive in days, dS_mode is death rate of susceptible cells (per day), lBt0_mode is viral infectivity β on a log10 scale, lp_mode is viral production rate π on a log10 scale, dI_mode is the rate multiplying the non-linear killing term, and n_mode is the exponent of the non-linear killing term
