# Supplementary material for: High monoclonal neutralization titers reduced breakthrough HIV-1 viral loads in the Antibody Mediated Prevention trials
Source: Nat Commun. 2023 Dec 14;14:8299. doi: 10.1038/s41467-023-43384-y (PMC10721814; doi:10.1038/s41467-023-43384-y)
Supplement: Supplementary file 4 — Supplementary Data 1 [file 41467_2023_43384_MOESM4_ESM.pdf]

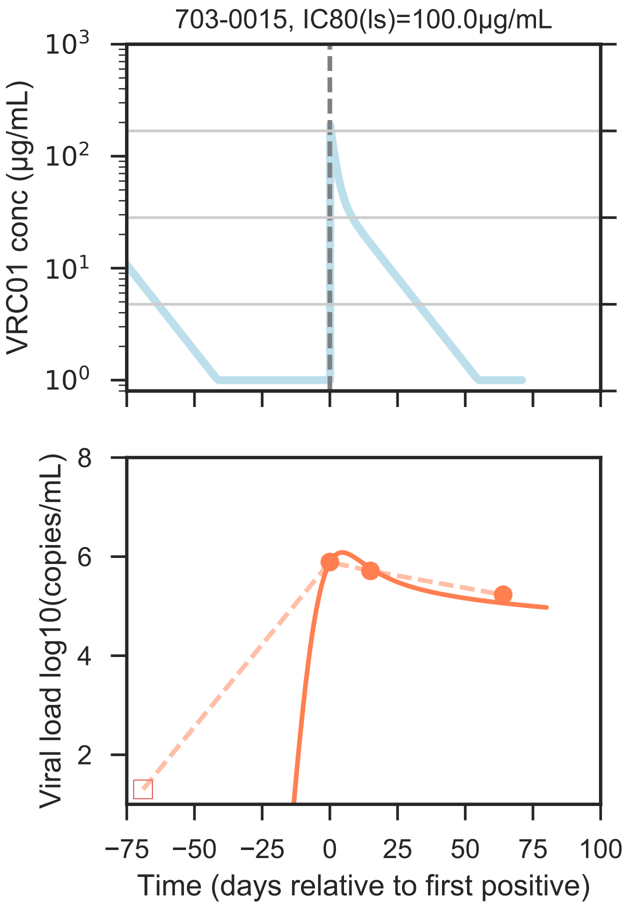

703-0015, IC80(ls)=100.0μg/mL
VRC01 conc (μg/mL)
log10 Titer
Viral load log10(copies/mL)
Time (days relative to first positive)

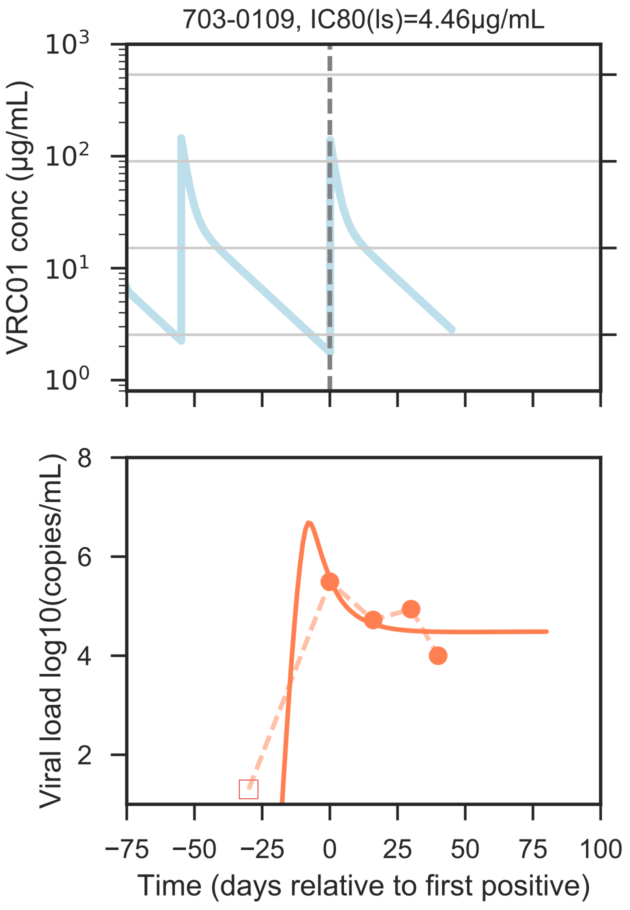

703-0109, IC80(ls)=4.46μg/mL
VRC01 conc (μg/mL)
log10 Titer
Viral load log10(copies/mL)
Time (days relative to first positive)

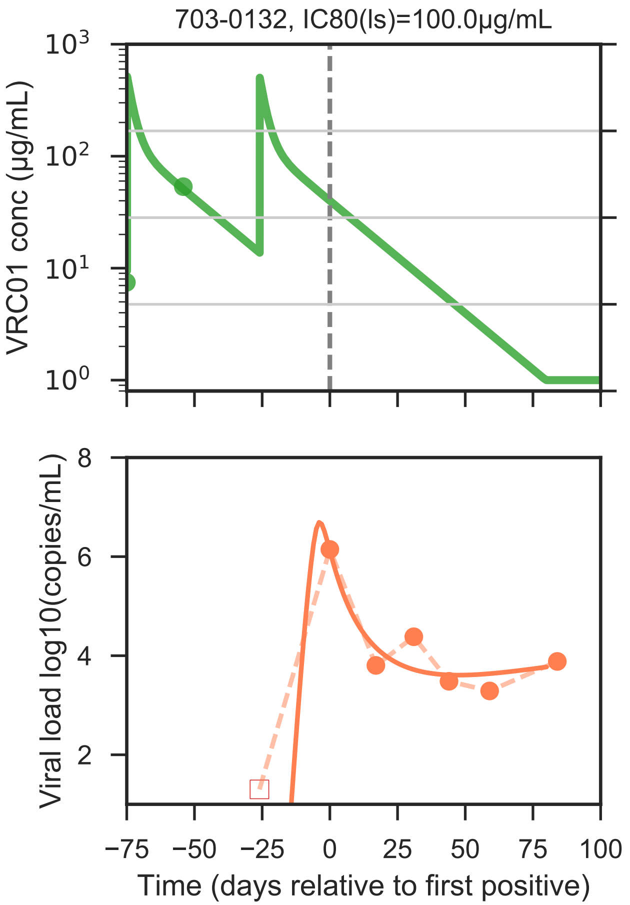

703-0132, IC80(ls)=100.0μg/mL
VRC01 conc (μg/mL)
log10 Titer
Viral load log10(copies/mL)
Time (days relative to first positive)

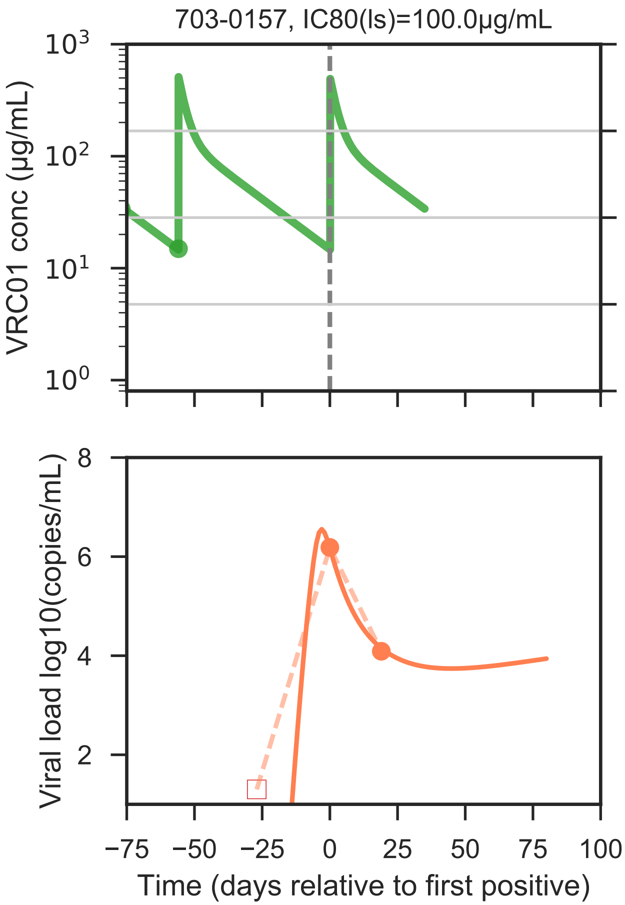

703-0157, IC80(ls)=100.0μg/mL
VRC01 conc (μg/mL)
log10 Titer
10^3
10^2
10^1
10^0
1
0
−1
−2
−3
Viral load log10(copies/mL)
8
6
4
2
−75
−50
−25
0
25
50
75
100
Time (days relative to first positive)

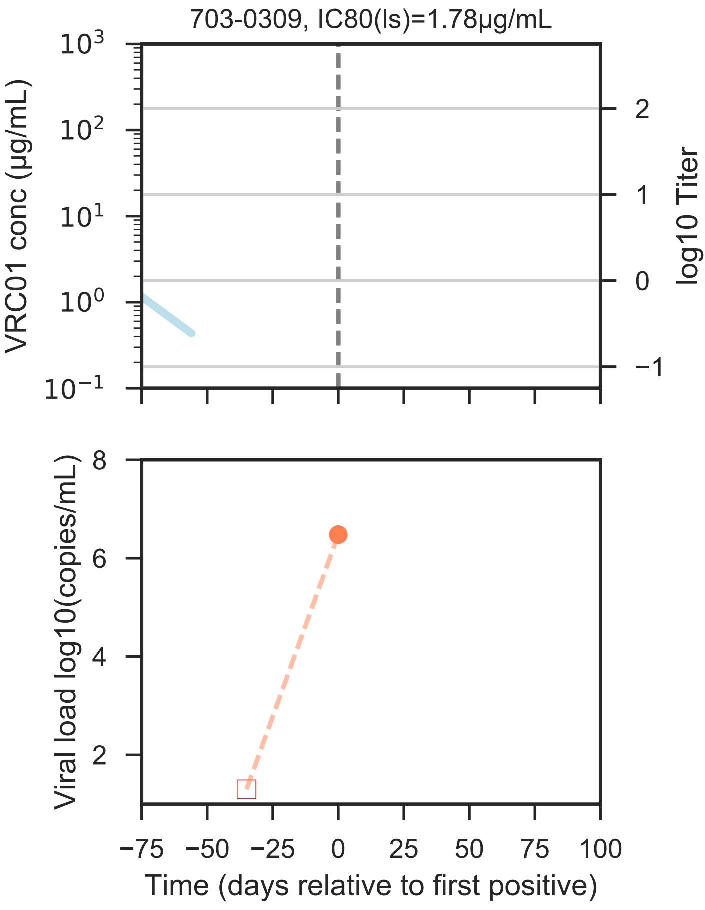

703-0309, IC80(ls)=1.78μg/mL
VRC01 conc (μg/mL)
log10 Titer
Viral load log10(copies/mL)
Time (days relative to first positive)

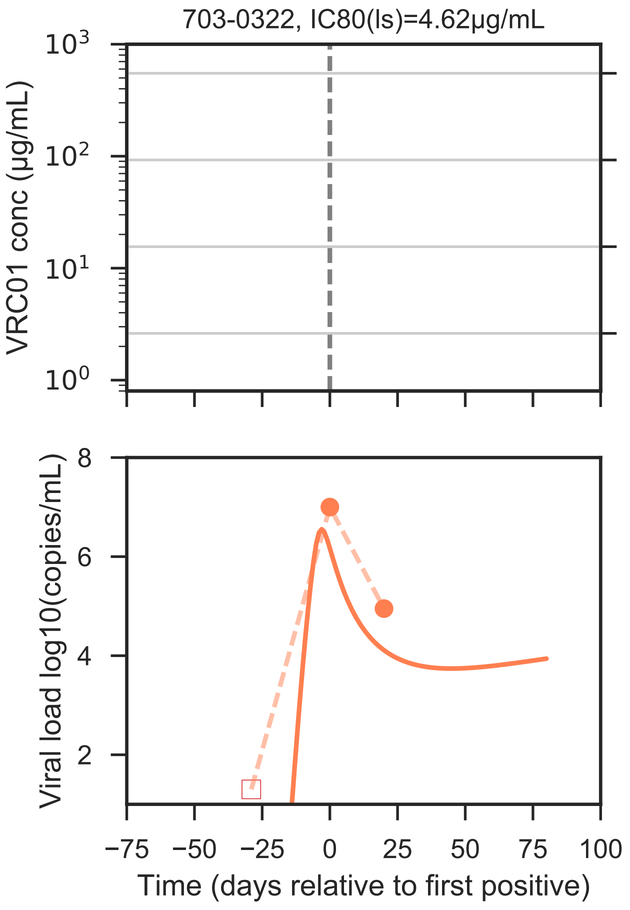

703-0322, IC80(ls)=4.62μg/mL
VRC01 conc (μg/mL)
log10 Titer
10^3
10^2
10^1
10^0
2
1
0
−1
Viral load log10(copies/mL)
8
6
4
2
−75
−50
−25
0
25
50
75
100
Time (days relative to first positive)

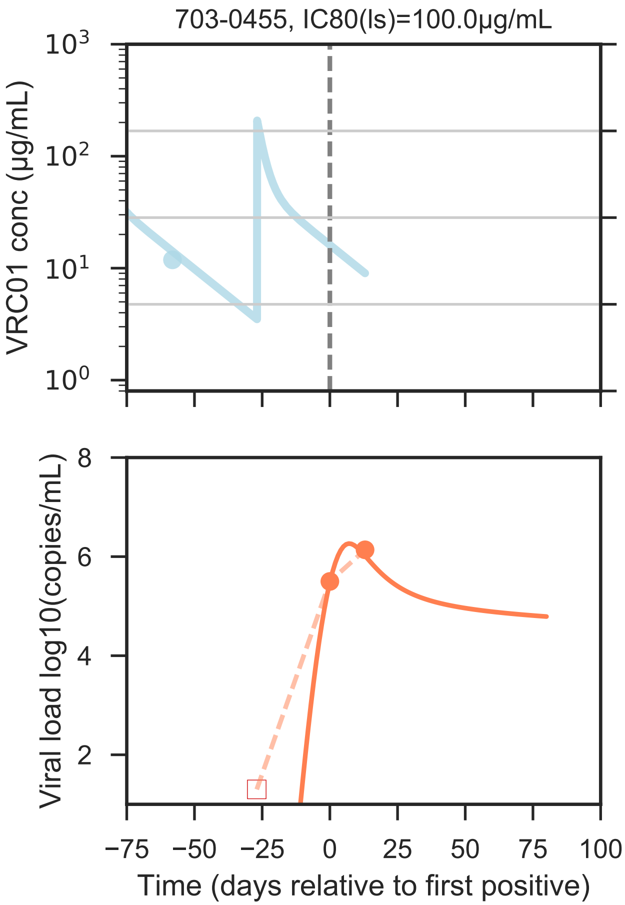

703-0455, IC80(ls)=100.0μg/mL
VRC01 conc (μg/mL)
log10 Titer
Viral load log10(copies/mL)
Time (days relative to first positive)

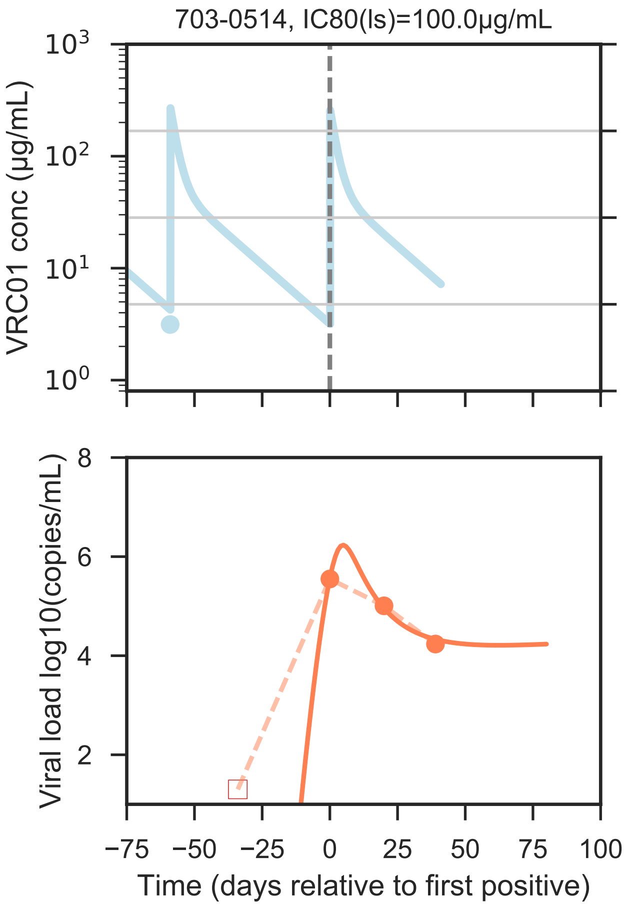

703-0514, IC80(ls)=100.0μg/mL
VRC01 conc (μg/mL)
log10 Titer
Viral load log10(copies/mL)
Time (days relative to first positive)

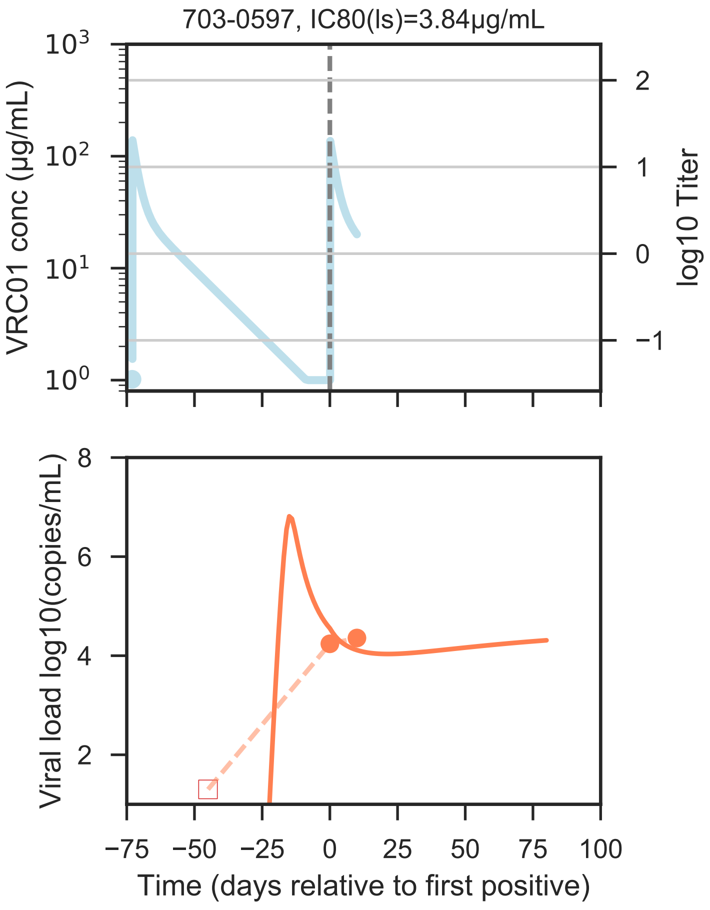

703-0597, IC80(ls)=3.84μg/mL
VRC01 conc (μg/mL)
log10 Titer
Viral load log10(copies/mL)
Time (days relative to first positive)

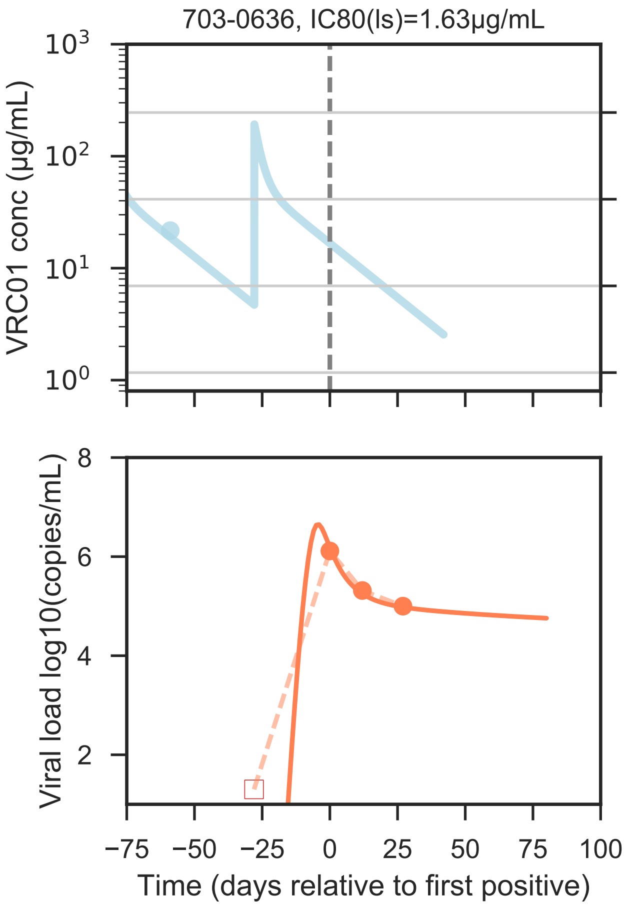

703-0636, IC80(ls)=1.63μg/mL
VRC01 conc (μg/mL)
log10 Titer
Viral load log10(copies/mL)
Time (days relative to first positive)

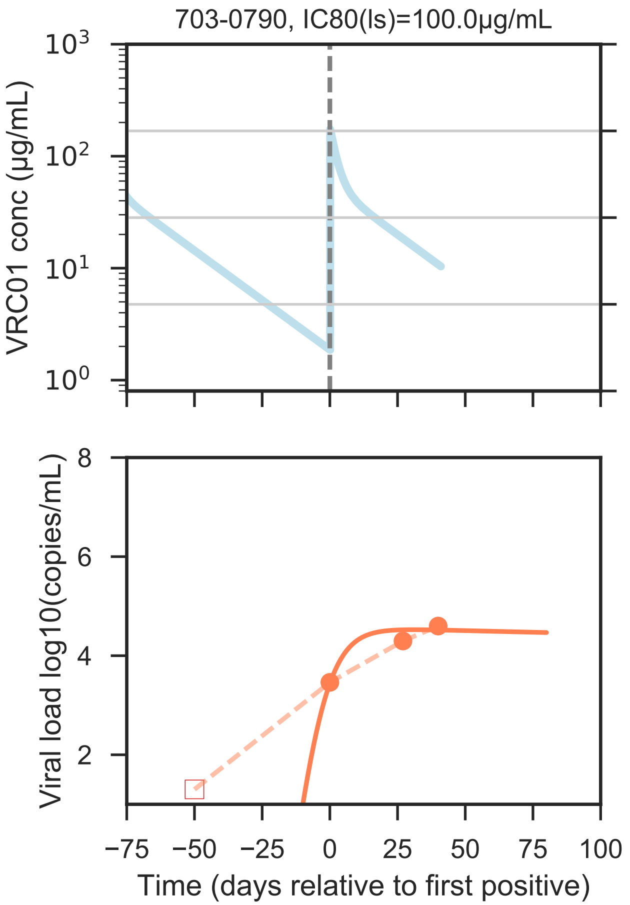

703-0790, IC80(ls)=100.0μg/mL
VRC01 conc (μg/mL)
log10 Titer
10^3
10^2
10^1
10^0
1
0
−1
−2
−3
Viral load log10(copies/mL)
8
6
4
2
−75
−50
−25
0
25
50
75
100
Time (days relative to first positive)

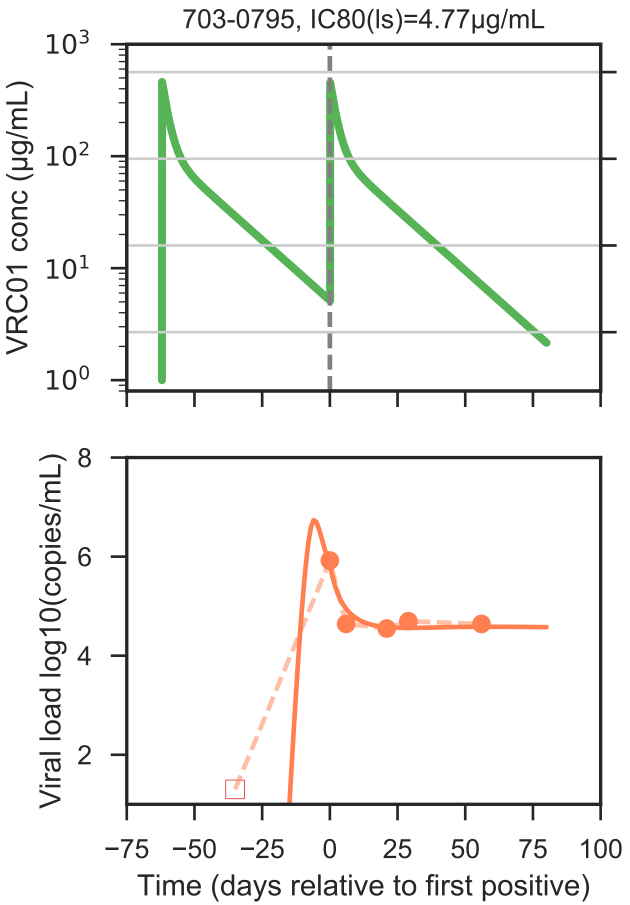
703-0795, IC80(ls)=4.77μg/mL
VRC01 conc (μg/mL)
log10 Titer
10^0
10^1
10^2
10^3
2
1
0
−1
Viral load log10(copies/mL)
8
6
4
2
−75
−50
−25
0
25
50
75
100
Time (days relative to first positive)

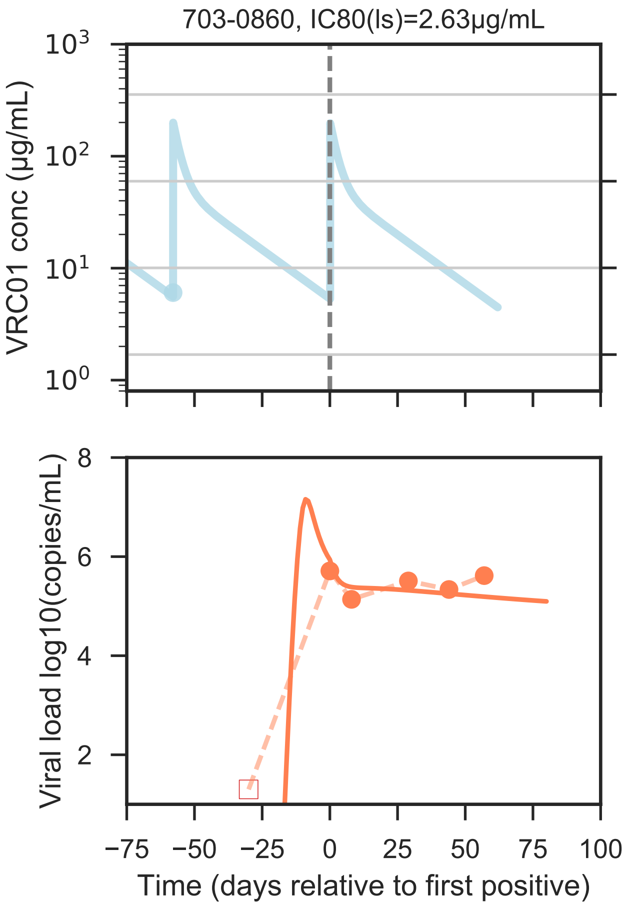

703-0860, IC80(ls)=2.63μg/mL
VRC01 conc (μg/mL)
log10 Titer
Viral load log10(copies/mL)
Time (days relative to first positive)

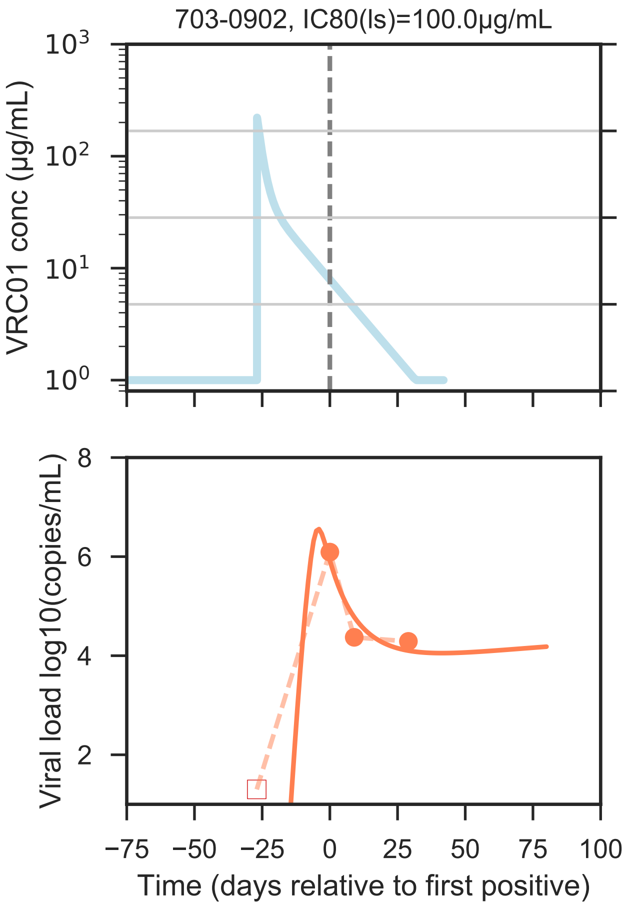

703-0902, IC80(ls)=100.0µg/mL
VRC01 conc (µg/mL)
log10 Titer
Viral load log10(copies/mL)
Time (days relative to first positive)

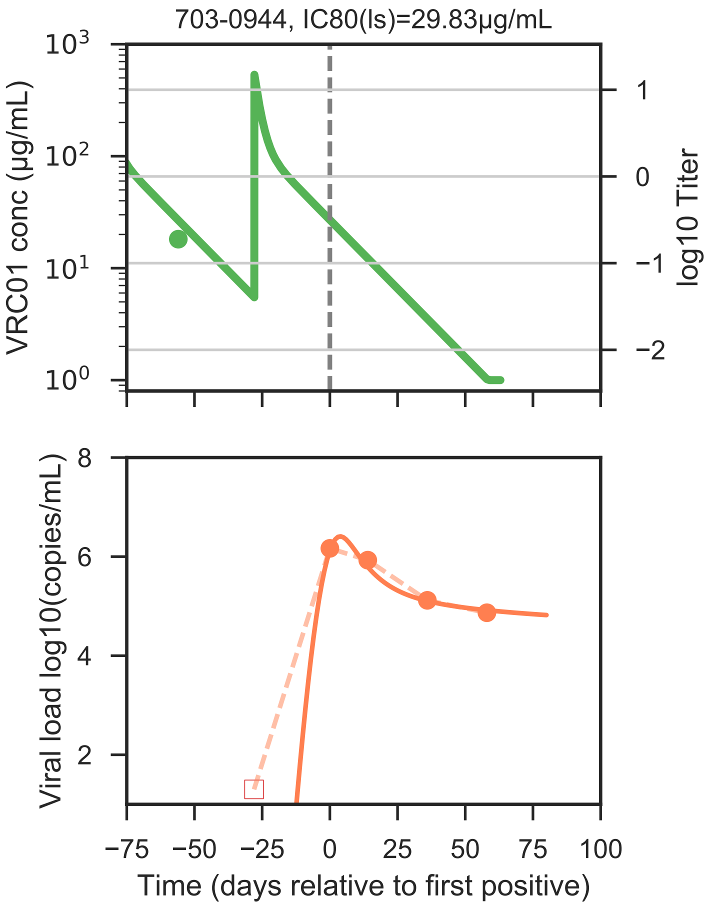

703-0944, IC80(ls)=29.83μg/mL
VRC01 conc (μg/mL)
log10 Titer
Viral load log10(copies/mL)
Time (days relative to first positive)

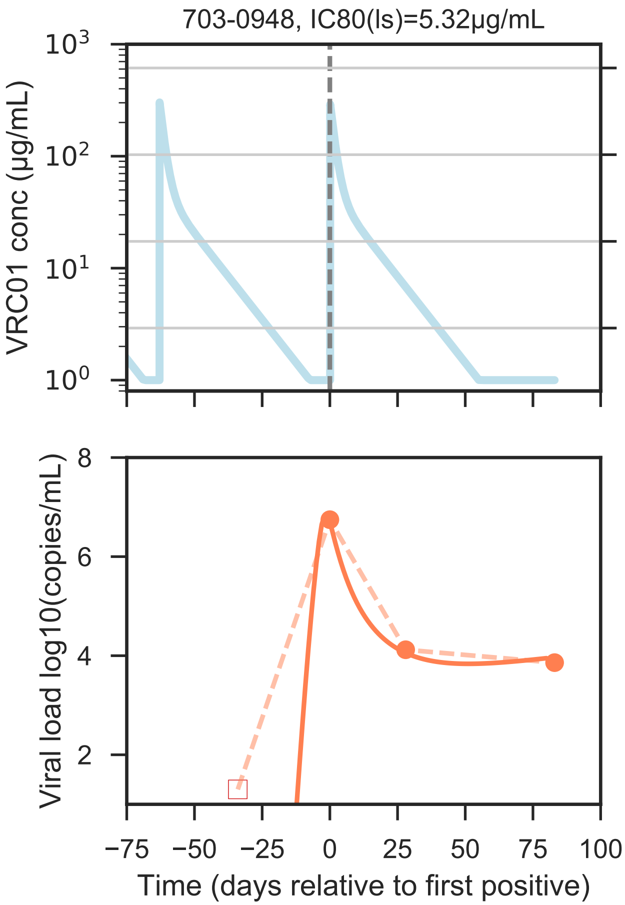

703-0948, IC80(ls)=5.32μg/mL
VRC01 conc (μg/mL)
log10 Titer
Viral load log10(copies/mL)
Time (days relative to first positive)

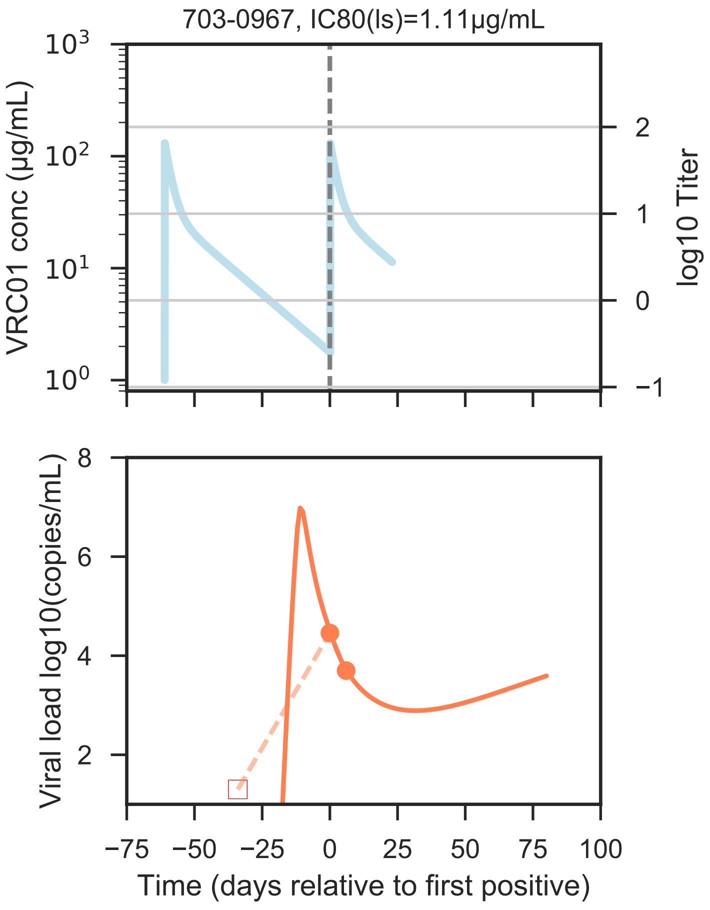

703-0967, IC80(ls)=1.11μg/mL
VRC01 conc (μg/mL)
log10 Titer
Viral load log10(copies/mL)
Time (days relative to first positive)

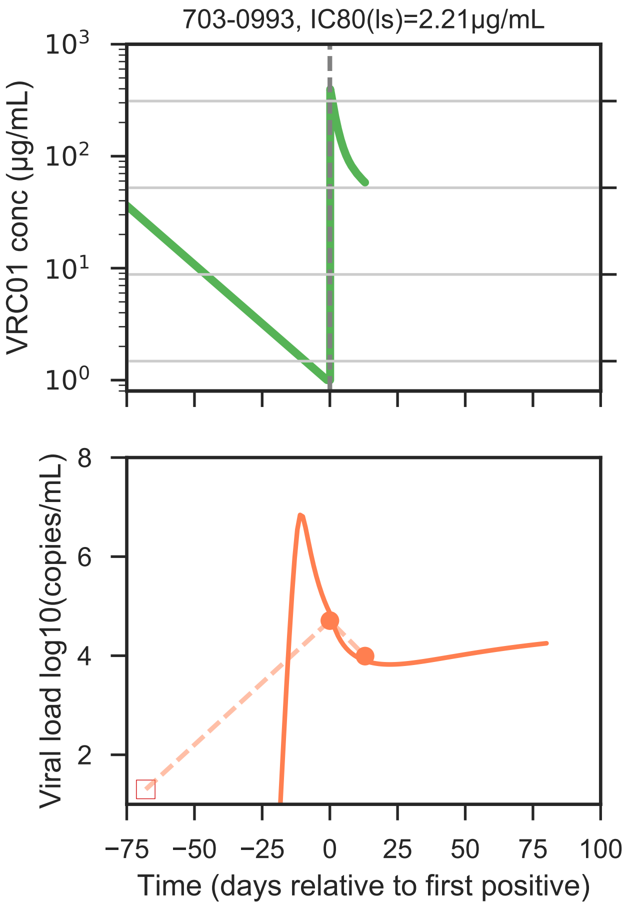

703-0993, IC80(ls)=2.21μg/mL
VRC01 conc (μg/mL)
log10 Titer
Viral load log10(copies/mL)
Time (days relative to first positive)

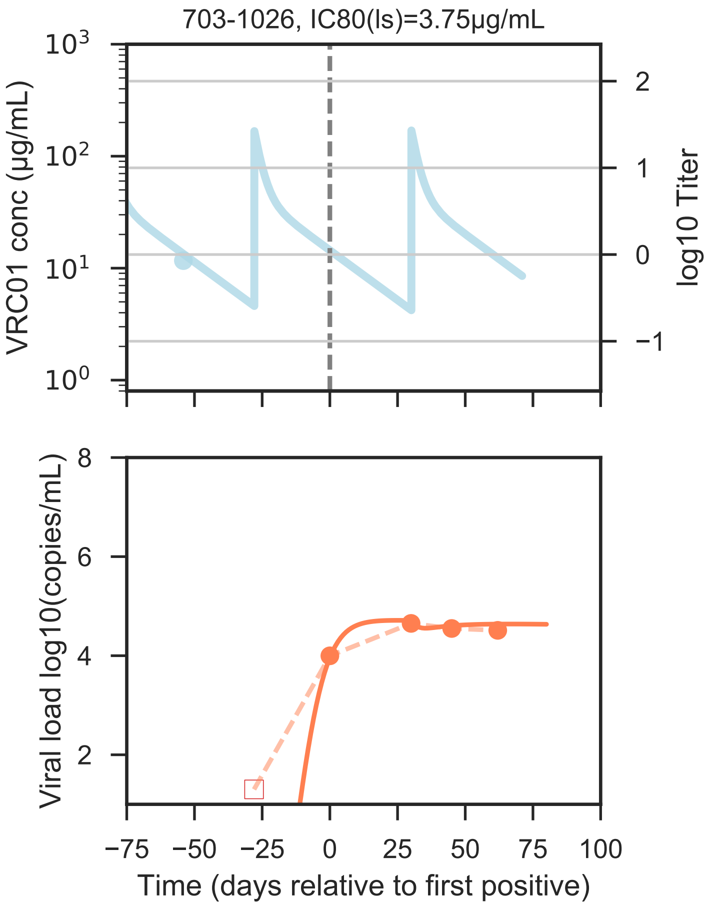

703-1026, IC80(ls)=3.75μg/mL
VRC01 conc (μg/mL)
log10 Titer
Viral load log10(copies/mL)
Time (days relative to first positive)

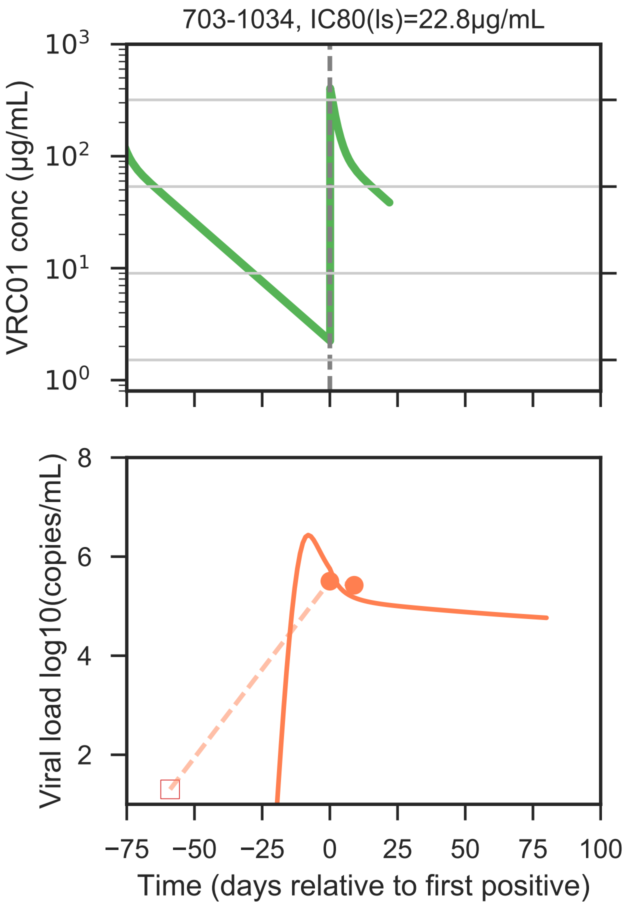

703-1034, IC80(ls)=22.8μg/mL
VRC01 conc (μg/mL)
log10 Titer
10^3
10^2
10^1
10^0
1
0
−1
−2
Viral load log10(copies/mL)
8
6
4
2
−75
−50
−25
0
25
50
75
100
Time (days relative to first positive)

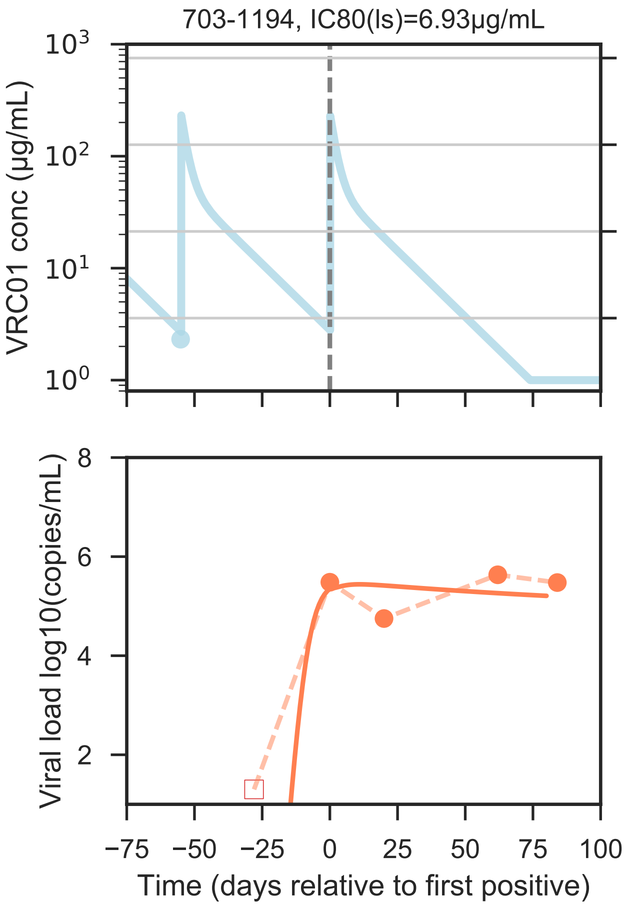

703-1194, IC80(ls)=6.93μg/mL
VRC01 conc (μg/mL)
log10 Titer
Viral load log10(copies/mL)
Time (days relative to first positive)

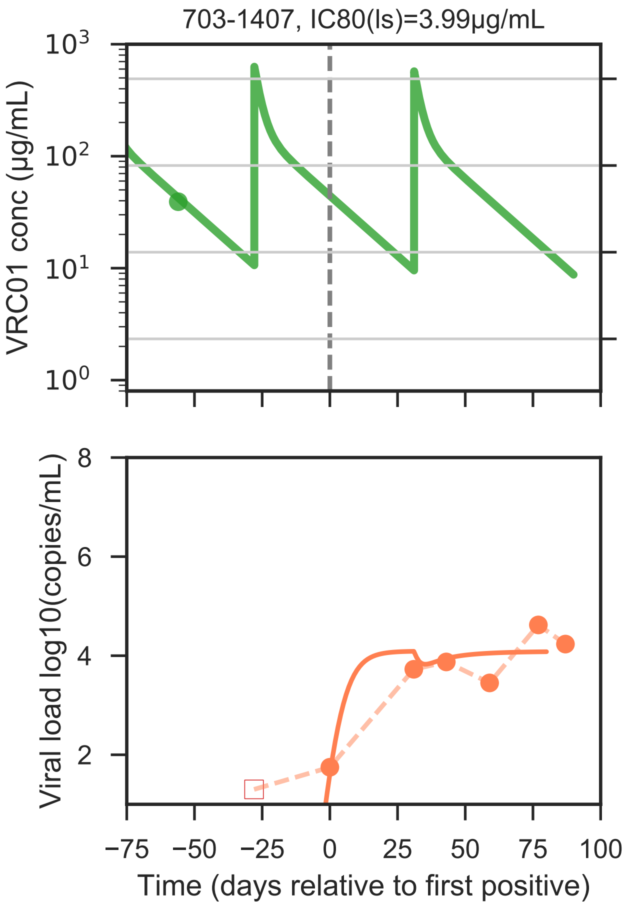

703-1407, IC80(ls)=3.99μg/mL
VRC01 conc (μg/mL)
log10 Titer
Viral load log10(copies/mL)
Time (days relative to first positive)

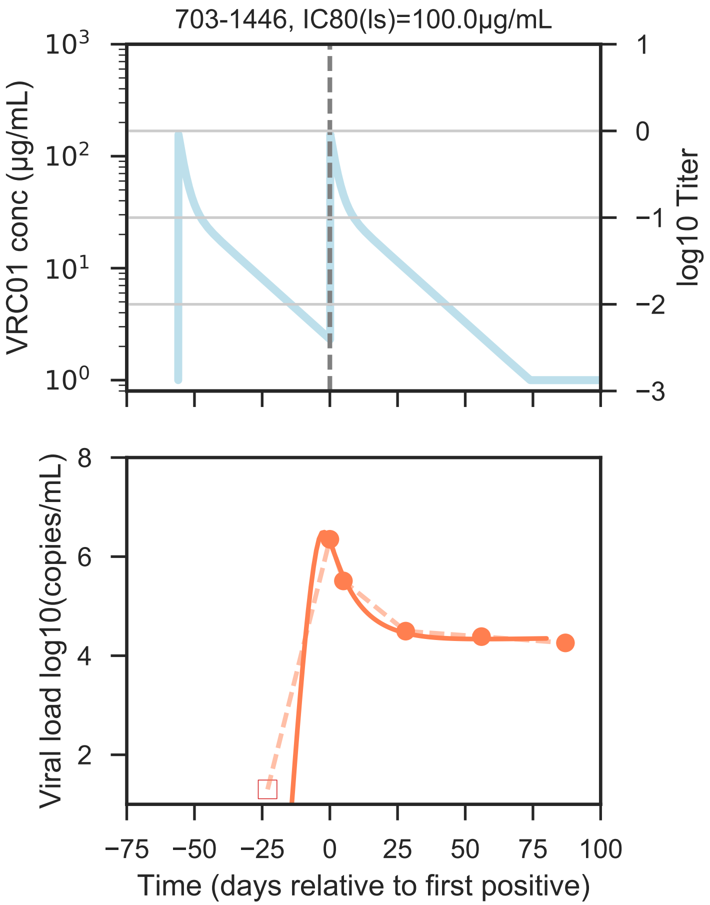

703-1446, IC80(ls)=100.0μg/mL
VRC01 conc (μg/mL)
log10 Titer
Viral load log10(copies/mL)
Time (days relative to first positive)

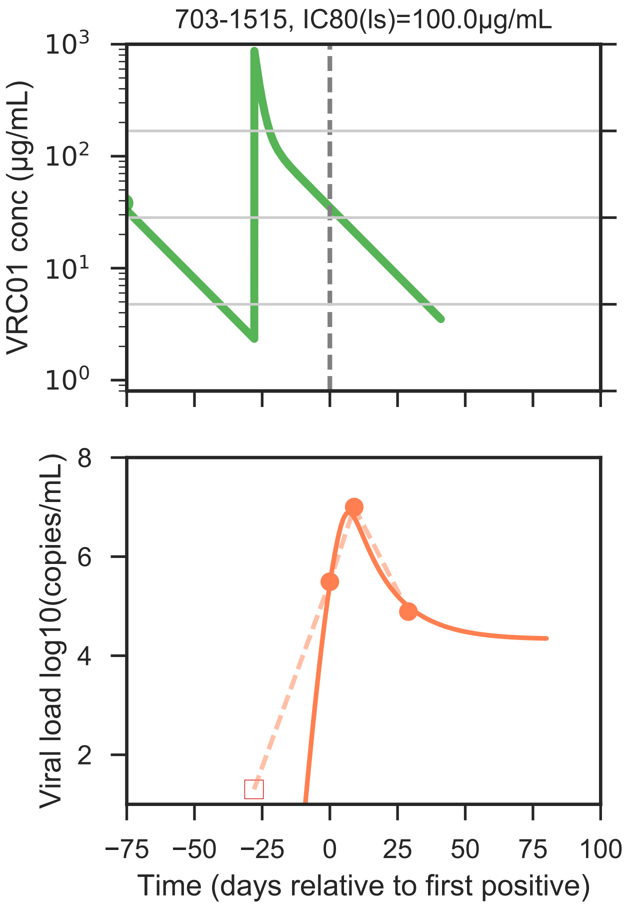

703-1515, IC80(ls)=100.0μg/mL
VRC01 conc (μg/mL)
log10 Titer
Viral load log10(copies/mL)
Time (days relative to first positive)

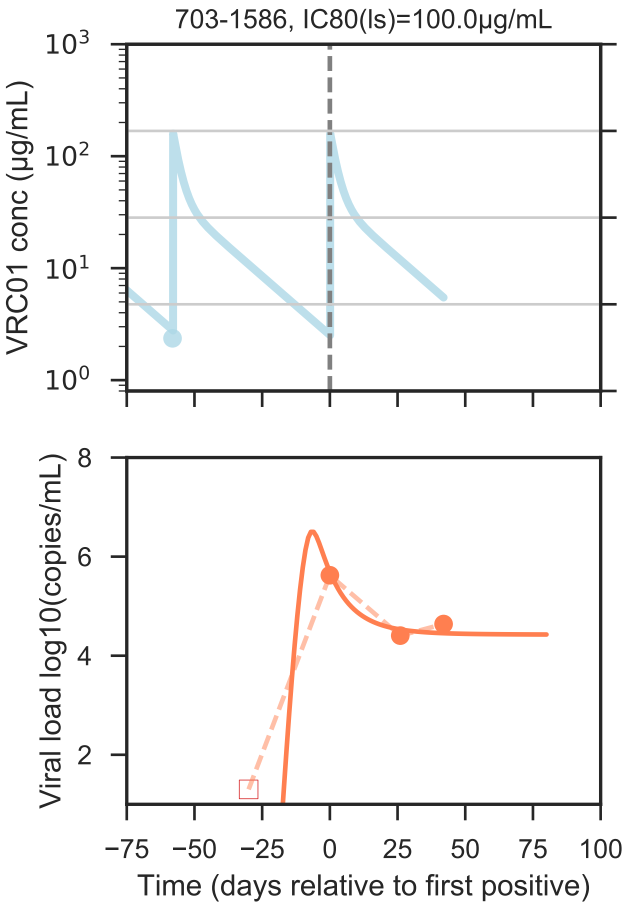

703-1586, IC80(ls)=100.0μg/mL
VRC01 conc (μg/mL)
10^3
10^2
10^1
10^0
log10 Titer
1
0
−1
−2
−3
Viral load log10(copies/mL)
8
6
4
2
−75
−50
−25
0
25
50
75
100
Time (days relative to first positive)

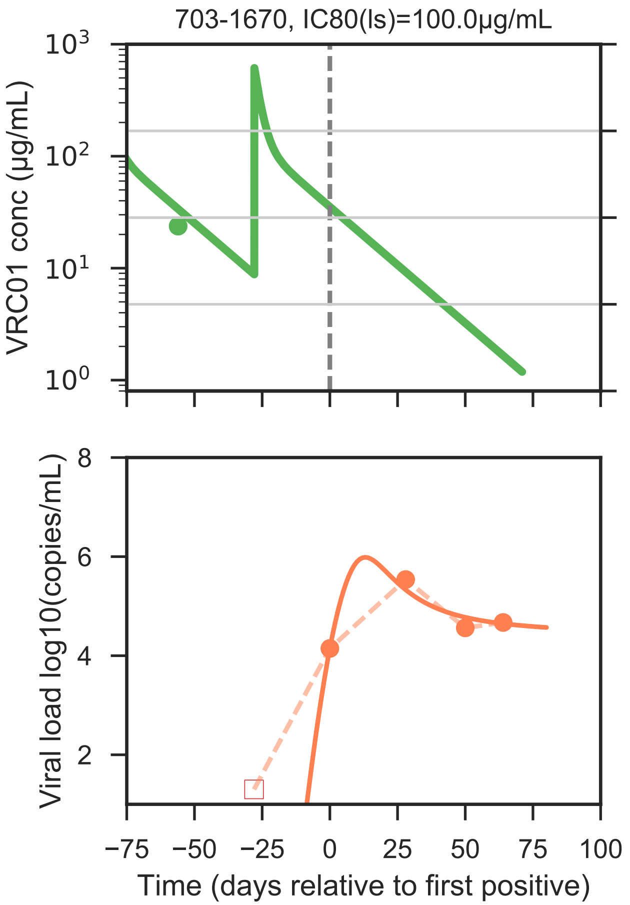

703-1670, IC80(ls)=100.0µg/mL
VRC01 conc (µg/mL)
log10 Titer
Viral load log10(copies/mL)
Time (days relative to first positive)

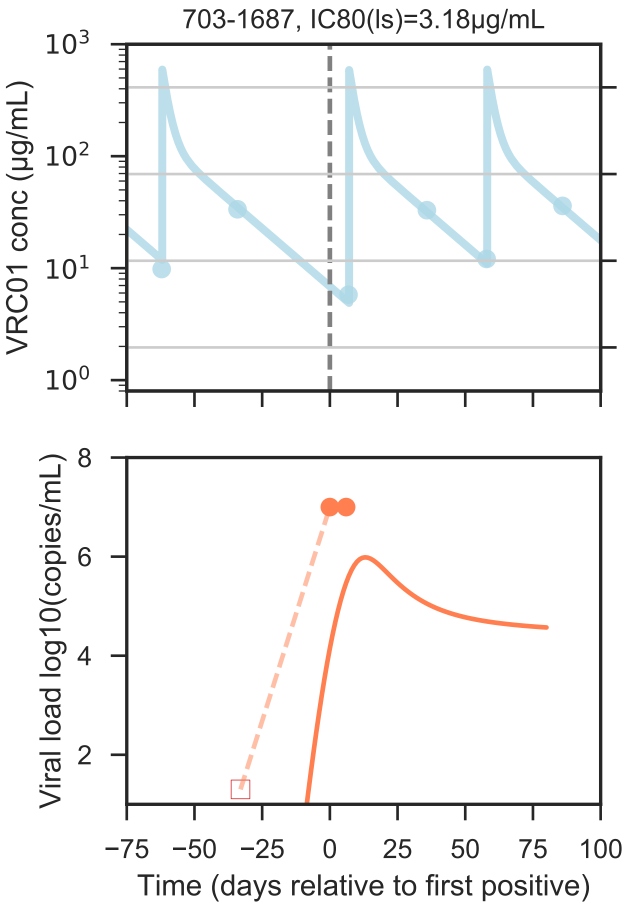

703-1687, IC80(ls)=3.18μg/mL
VRC01 conc (μg/mL)
log10 Titer
10^3
10^2
10^1
10^0
2
1
0
−1
Viral load log10(copies/mL)
8
6
4
2
−75
−50
−25
0
25
50
75
100
Time (days relative to first positive)

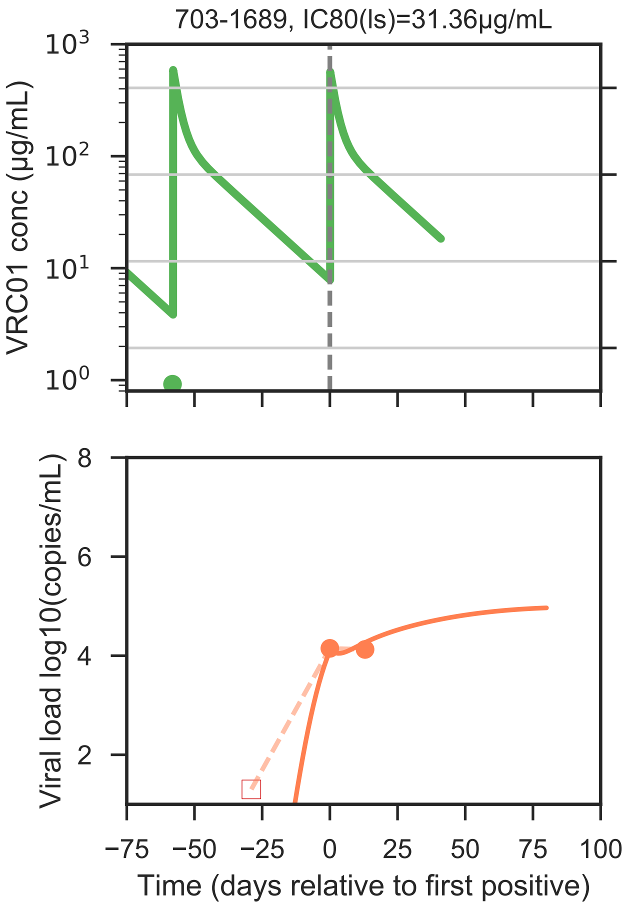

703-1689, IC80(ls)=31.36μg/mL
VRC01 conc (μg/mL)
log10 Titer
Viral load log10(copies/mL)
Time (days relative to first positive)

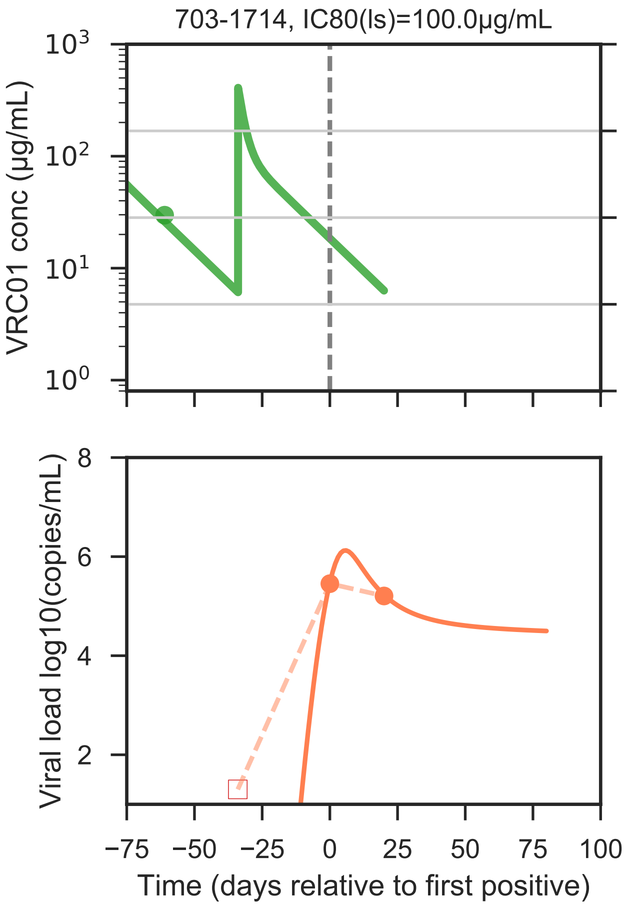

703-1714, IC80(ls)=100.0μg/mL
VRC01 conc (μg/mL)
log10 Titer
Viral load log10(copies/mL)
Time (days relative to first positive)

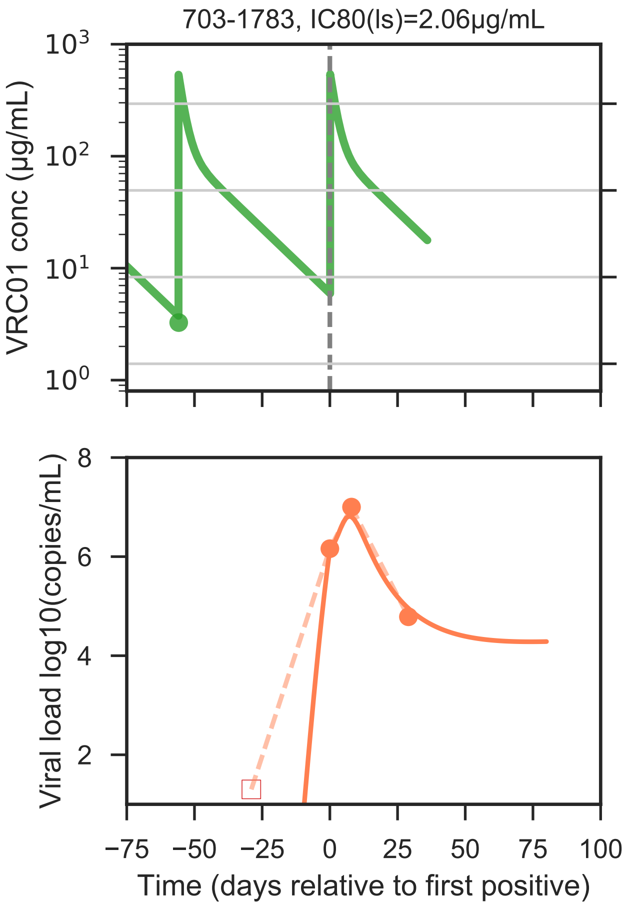

703-1783, IC80(ls)=2.06μg/mL
VRC01 conc (μg/mL)
log10 Titer
Viral load log10(copies/mL)
Time (days relative to first positive)

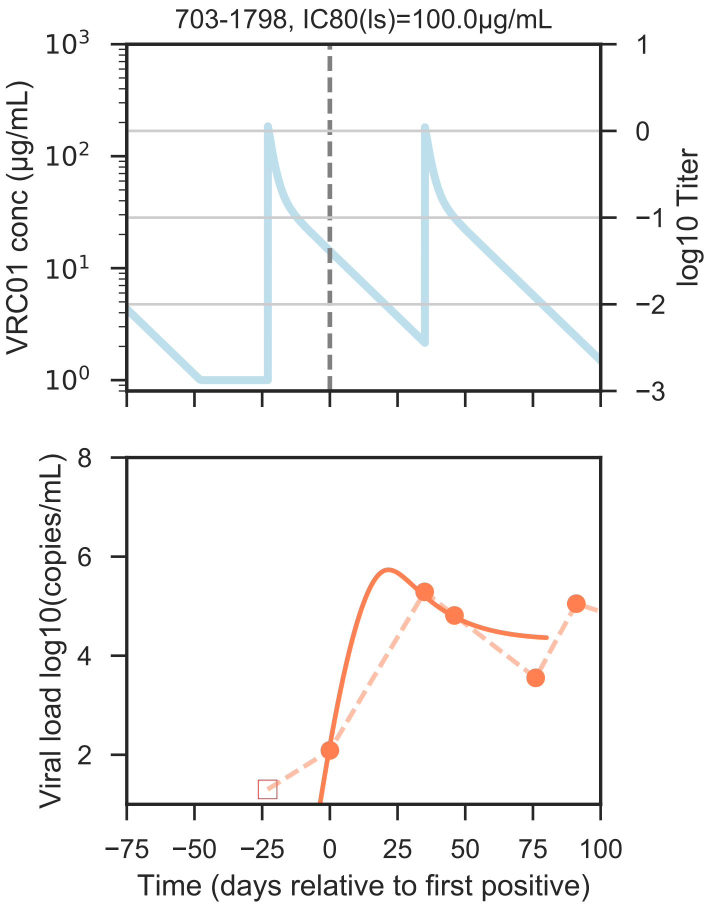
703-1798, IC80(ls)=100.0μg/mL
VRC01 conc (μg/mL)
log10 Titer
Viral load log10(copies/mL)
Time (days relative to first positive)

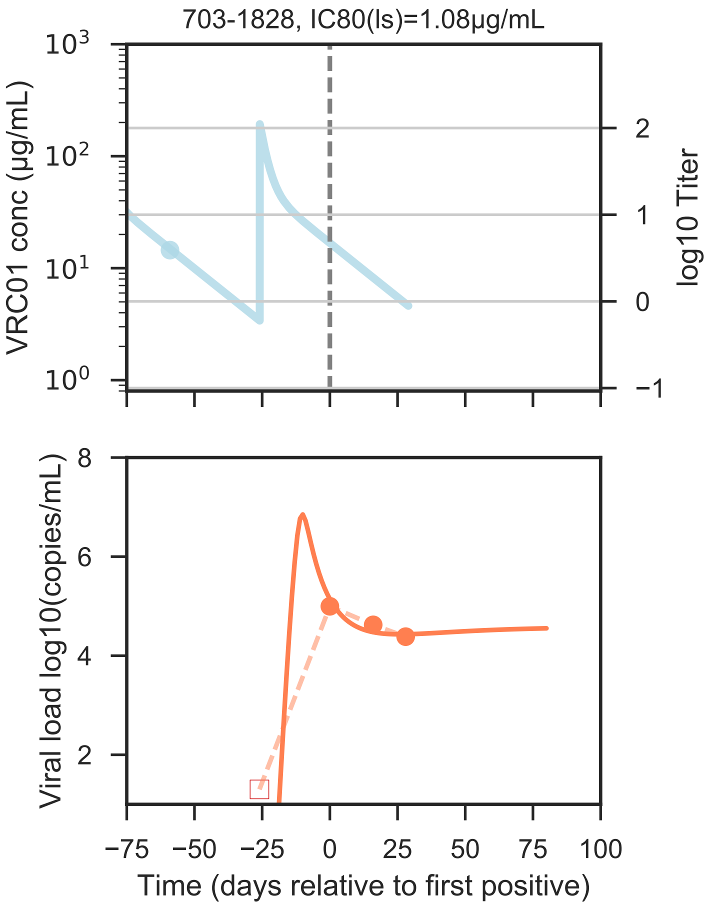

703-1828, IC80(ls)=1.08μg/mL
VRC01 conc (μg/mL)
log10 Titer
Viral load log10(copies/mL)
Time (days relative to first positive)

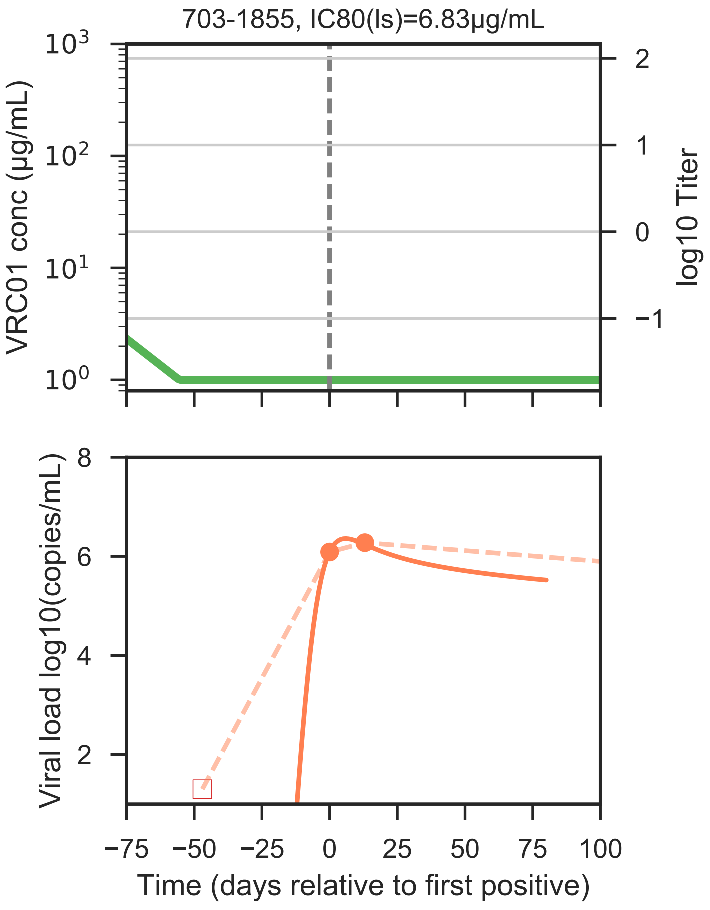

703-1855, IC80(ls)=6.83μg/mL
VRC01 conc (μg/mL)
log10 Titer
Viral load log10(copies/mL)
Time (days relative to first positive)

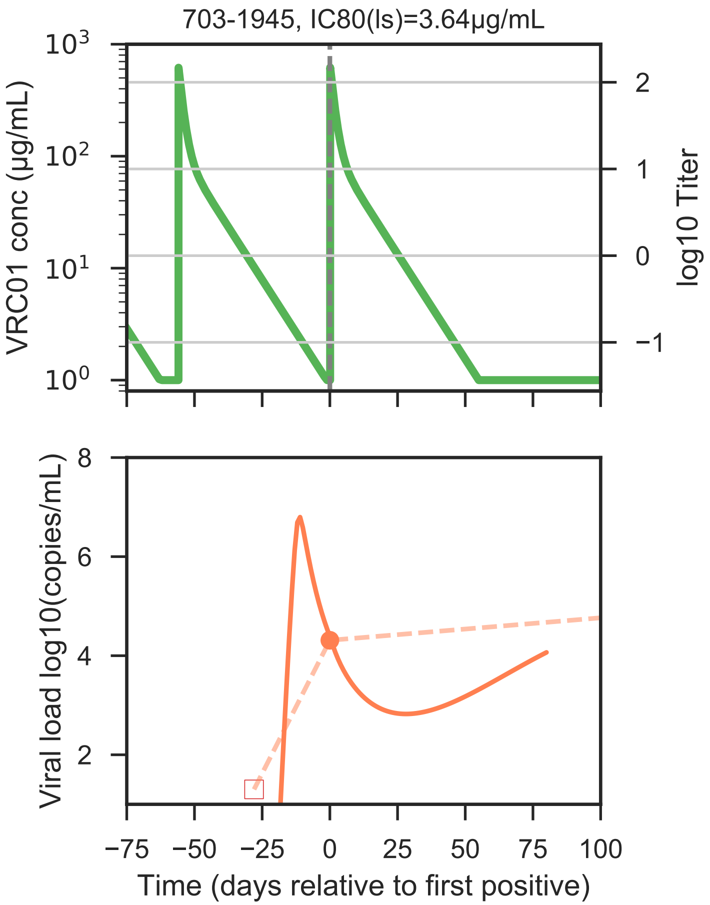
703-1945, IC80(ls)=3.64μg/mL
VRC01 conc (μg/mL)
log10 Titer
Viral load log10(copies/mL)
Time (days relative to first positive)

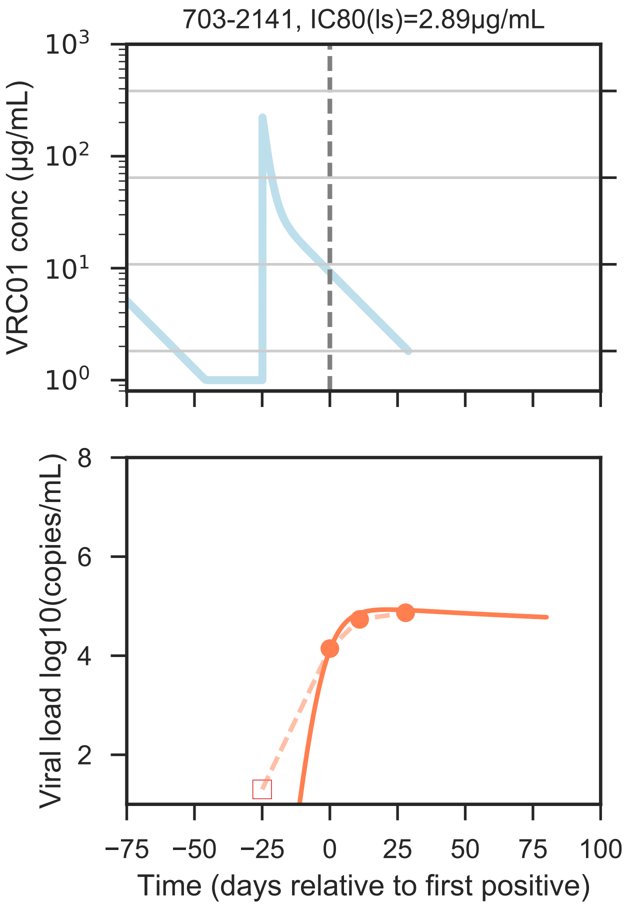

703-2141, IC80(ls)=2.89μg/mL
VRC01 conc (μg/mL)
log10 Titer
Viral load log10(copies/mL)
Time (days relative to first positive)

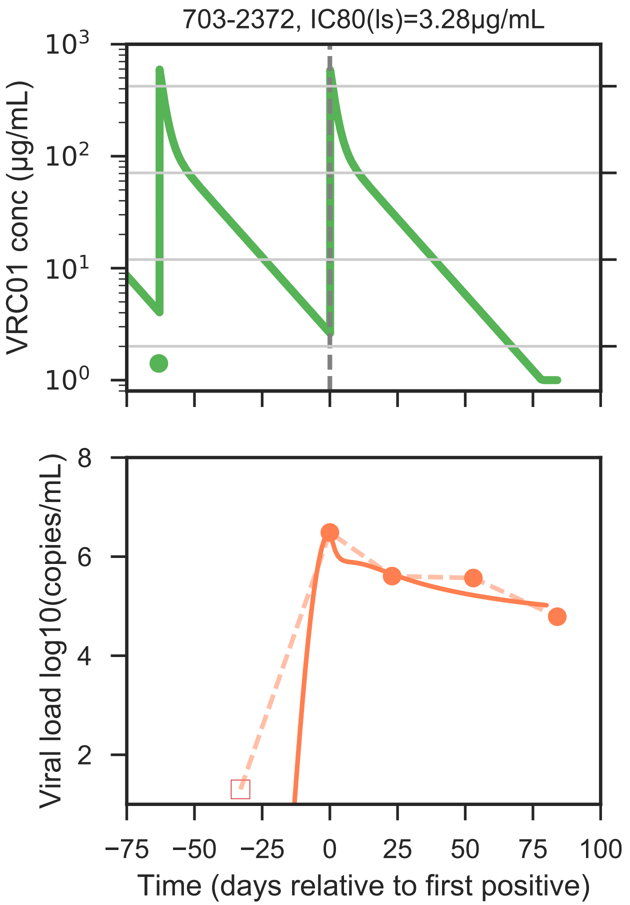

703-2372, IC80(ls)=3.28μg/mL
VRC01 conc (μg/mL)
log10 Titer
10^3
10^2
10^1
10^0
2
1
0
−1
Viral load log10(copies/mL)
8
6
4
2
−75
−50
−25
0
25
50
75
100
Time (days relative to first positive)

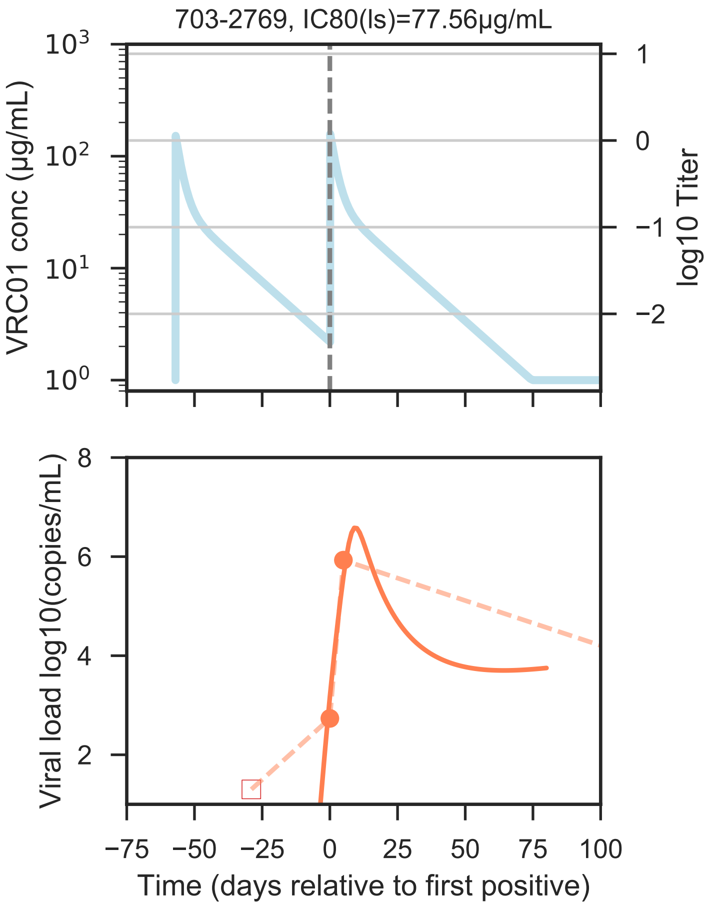

703-2769, IC80(ls)=77.56μg/mL
VRC01 conc (μg/mL)
log10 Titer
Viral load log10(copies/mL)
Time (days relative to first positive)

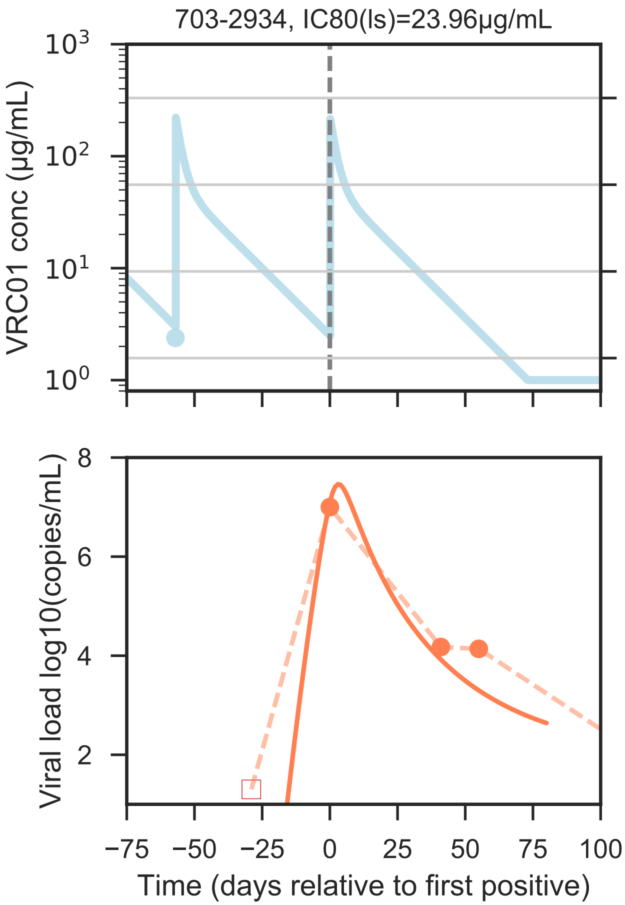

703-2934, IC80(ls)=23.96μg/mL
VRC01 conc (μg/mL)
log10 Titer
10^3
10^2
10^1
10^0
1
0
−1
−2
Viral load log10(copies/mL)
8
6
4
2
−75
−50
−25
0
25
50
75
100
Time (days relative to first positive)

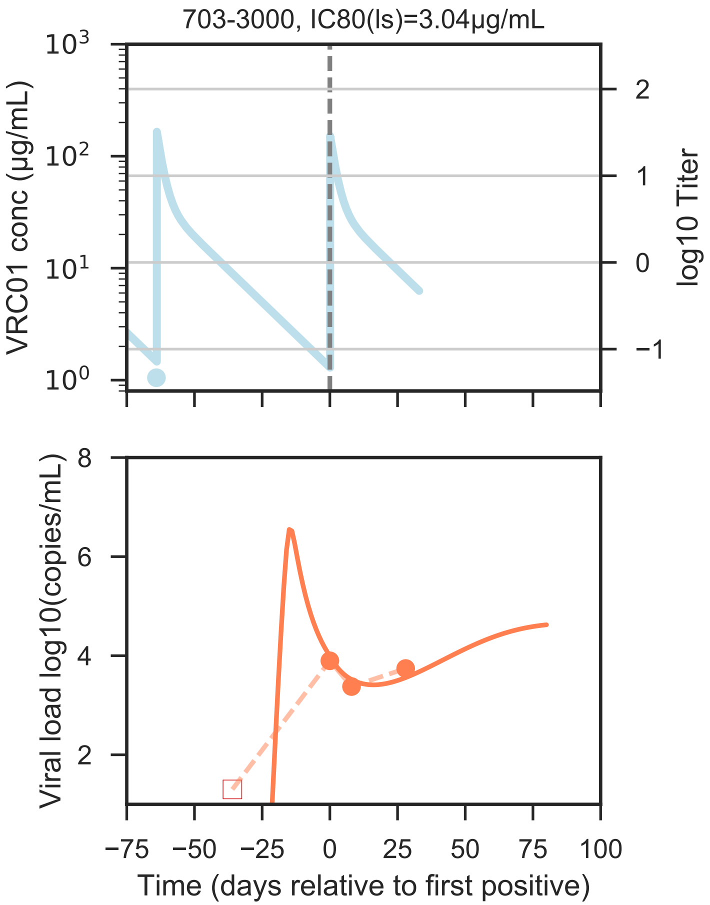

703-3000, IC80(ls)=3.04μg/mL
VRC01 conc (μg/mL)
log10 Titer
10^3
10^2
10^1
10^0
2
1
0
−1
Viral load log10(copies/mL)
8
6
4
2
−75
−50
−25
0
25
50
75
100
Time (days relative to first positive)

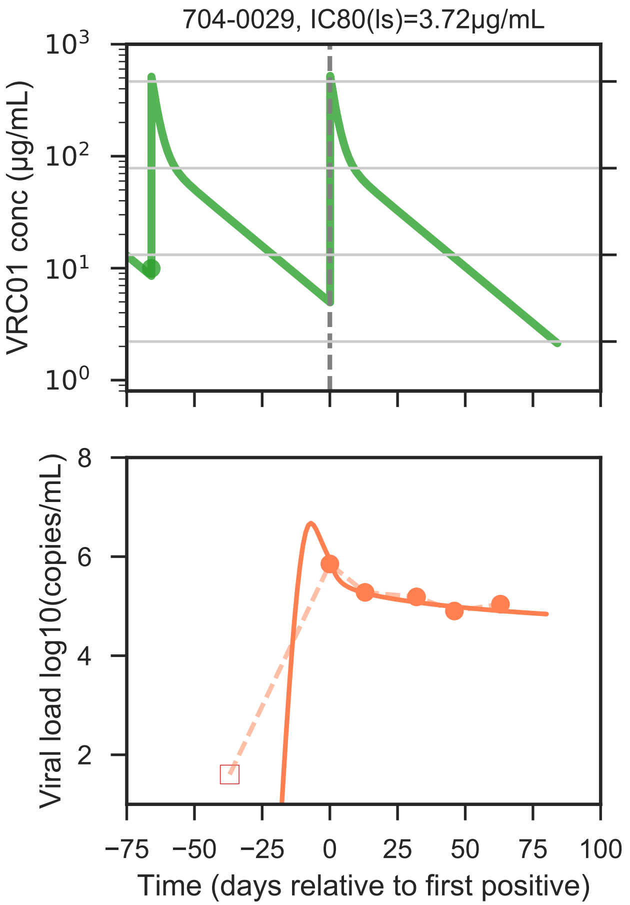

704-0029, IC80(ls)=3.72μg/mL
VRC01 conc (μg/mL)
log10 Titer
10^3
10^2
10^1
10^0
2
1
0
−1
Viral load log10(copies/mL)
8
6
4
2
−75
−50
−25
0
25
50
75
100
Time (days relative to first positive)

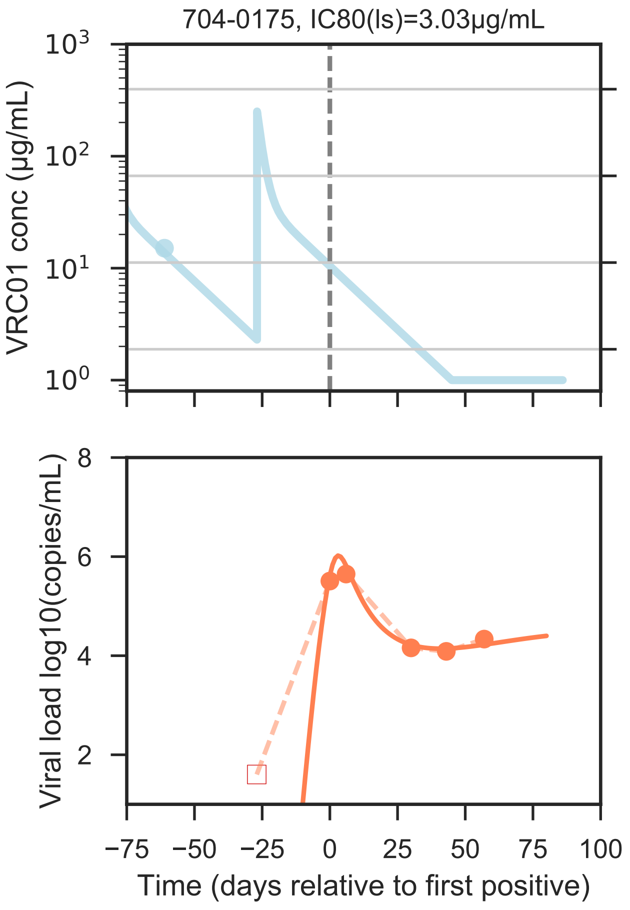

704-0175, IC80(ls)=3.03μg/mL
VRC01 conc (μg/mL)
log10 Titer
Viral load log10(copies/mL)
Time (days relative to first positive)

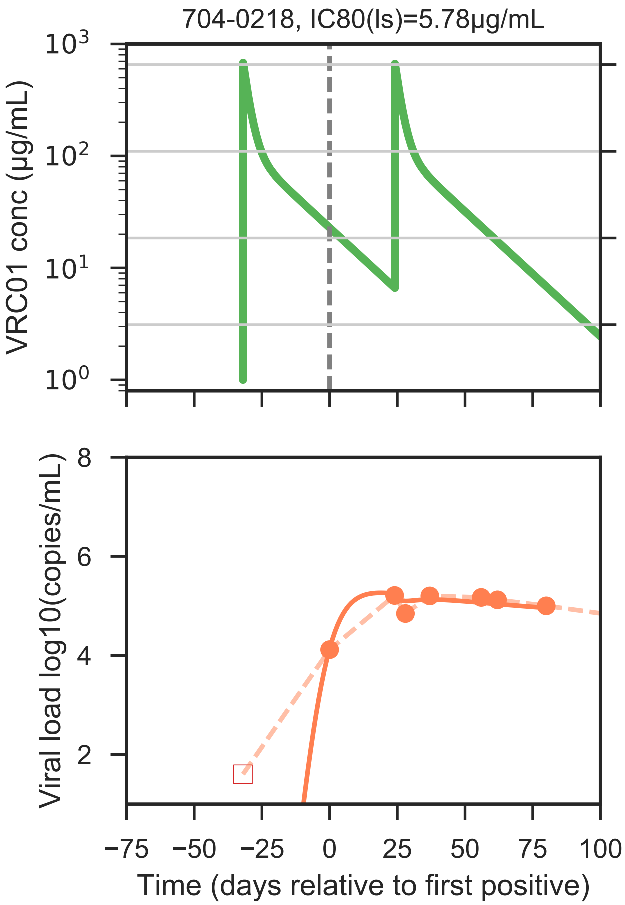

704-0218, IC80(ls)=5.78μg/mL
VRC01 conc (μg/mL)
log10 Titer
Viral load log10(copies/mL)
Time (days relative to first positive)

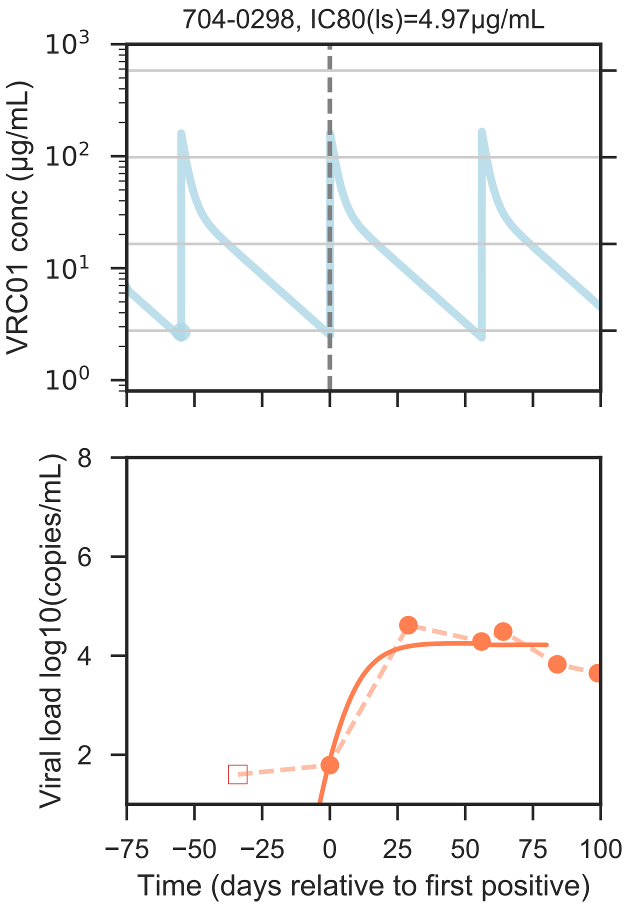
704-0298, IC80(ls)=4.97μg/mL
VRC01 conc (μg/mL)
log10 Titer
Viral load log10(copies/mL)
Time (days relative to first positive)

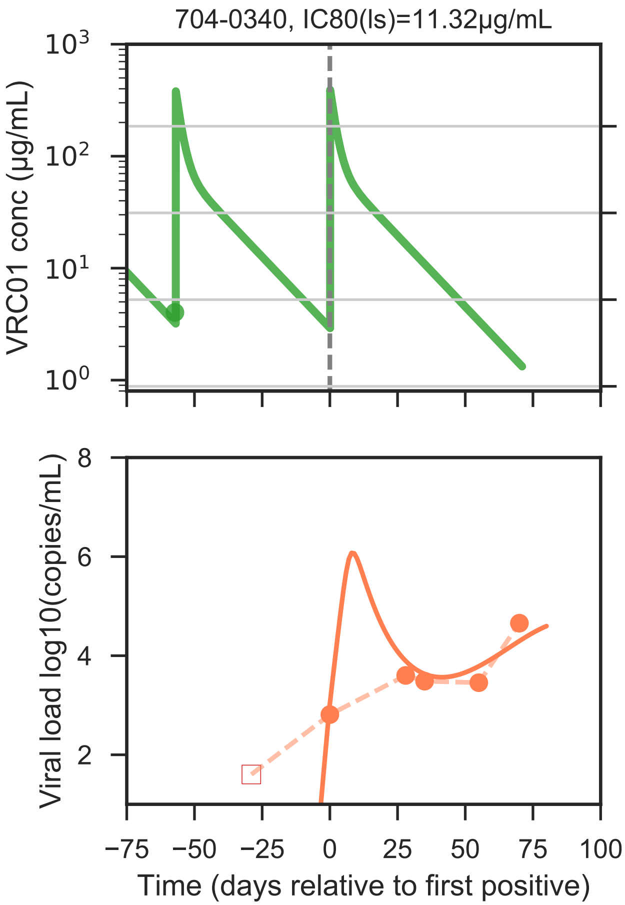

704-0340, IC80(ls)=11.32μg/mL
VRC01 conc (μg/mL)
log10 Titer
Viral load log10(copies/mL)
Time (days relative to first positive)

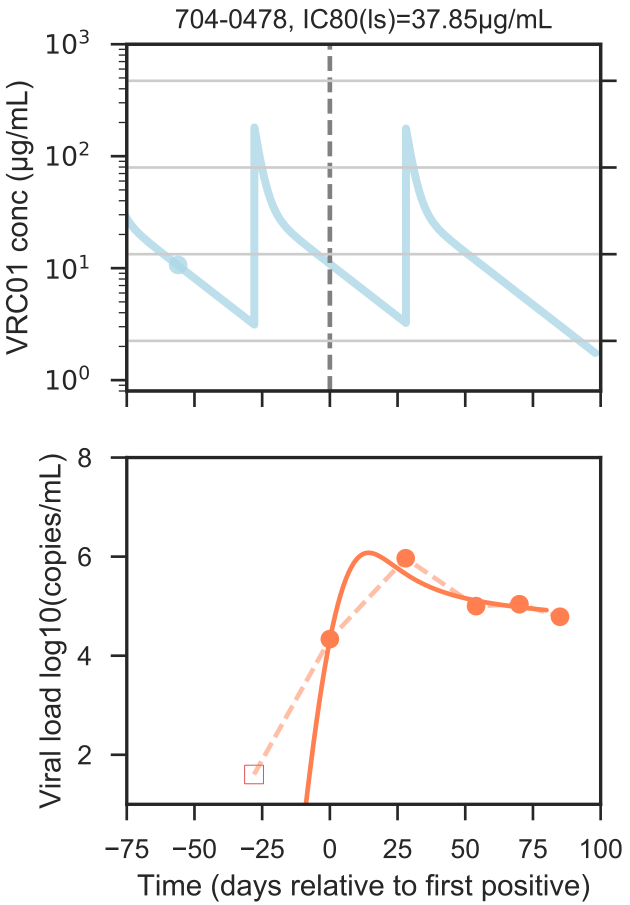

704-0478, IC80(ls)=37.85μg/mL
VRC01 conc (μg/mL)
10^3
10^2
10^1
10^0
log10 Titer
1
0
−1
−2
Viral load log10(copies/mL)
8
6
4
2
−75
−50
−25
0
25
50
75
100
Time (days relative to first positive)

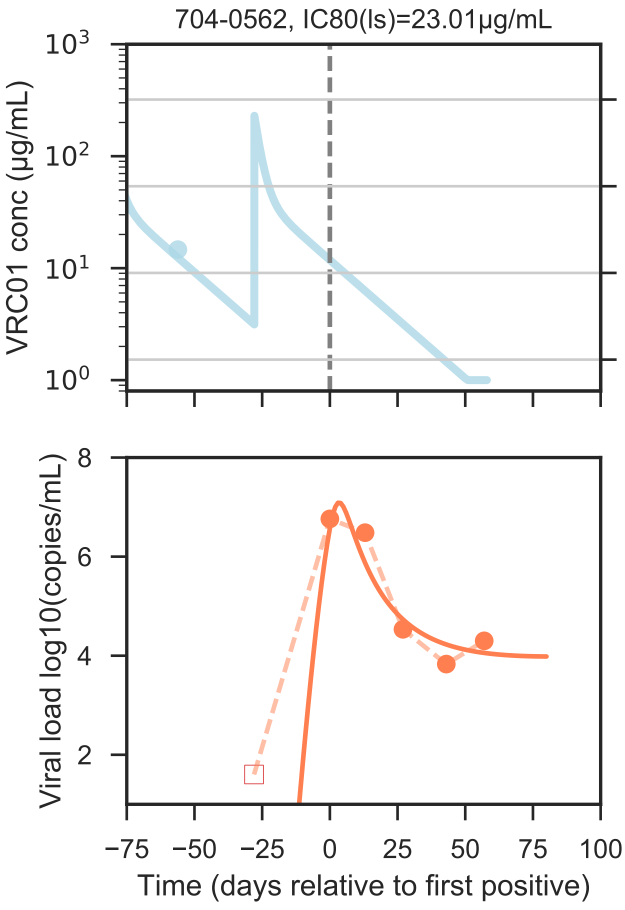

704-0562, IC80(ls)=23.01μg/mL
VRC01 conc (μg/mL)
log10 Titer
Viral load log10(copies/mL)
Time (days relative to first positive)

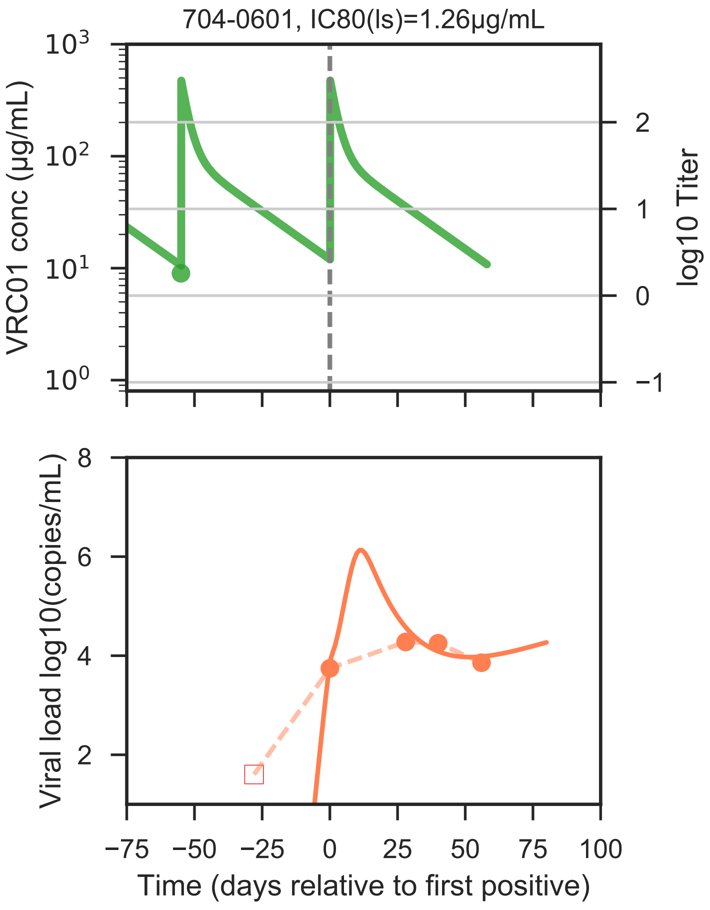
704-0601, IC80(ls)=1.26μg/mL
VRC01 conc (μg/mL)
10^3
10^2
10^1
10^0
log10 Titer
2
1
0
−1
Viral load log10(copies/mL)
8
6
4
2
−75
−50
−25
0
25
50
75
100
Time (days relative to first positive)

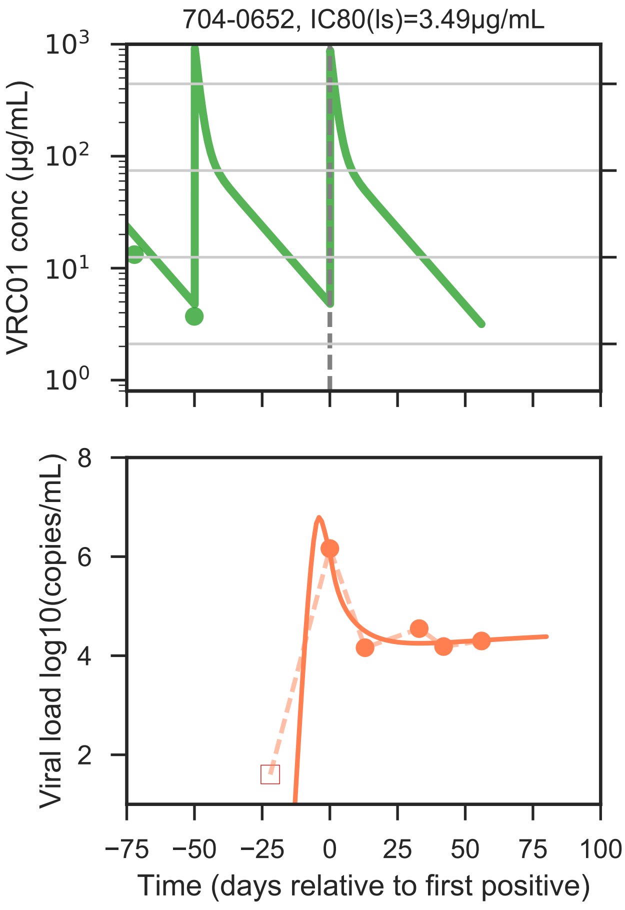
704-0652, IC80(ls)=3.49μg/mL
VRC01 conc (μg/mL)
log10 Titer
Viral load log10(copies/mL)
Time (days relative to first positive)

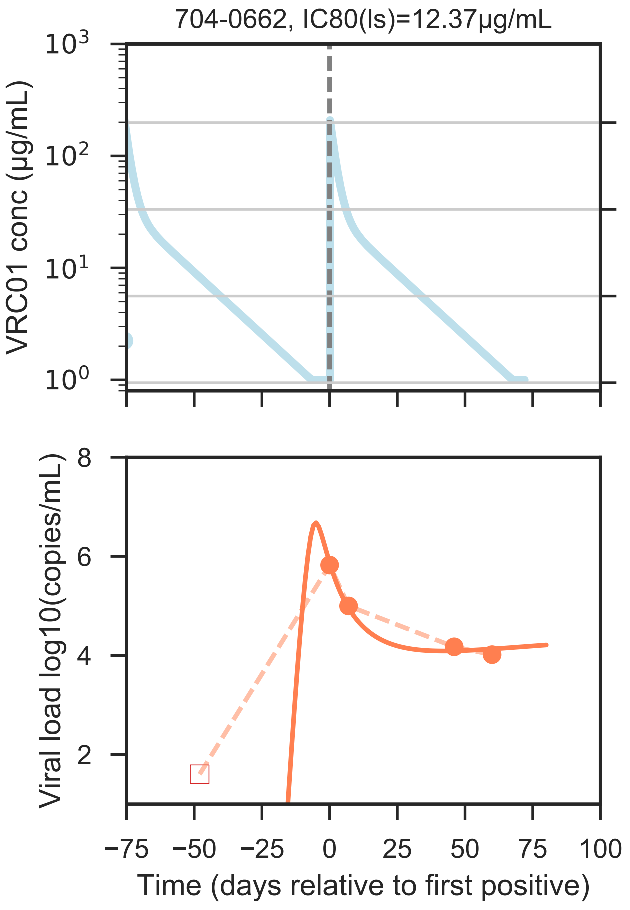
704-0662, IC80(ls)=12.37μg/mL
VRC01 conc (μg/mL)
log10 Titer
Viral load log10(copies/mL)
Time (days relative to first positive)

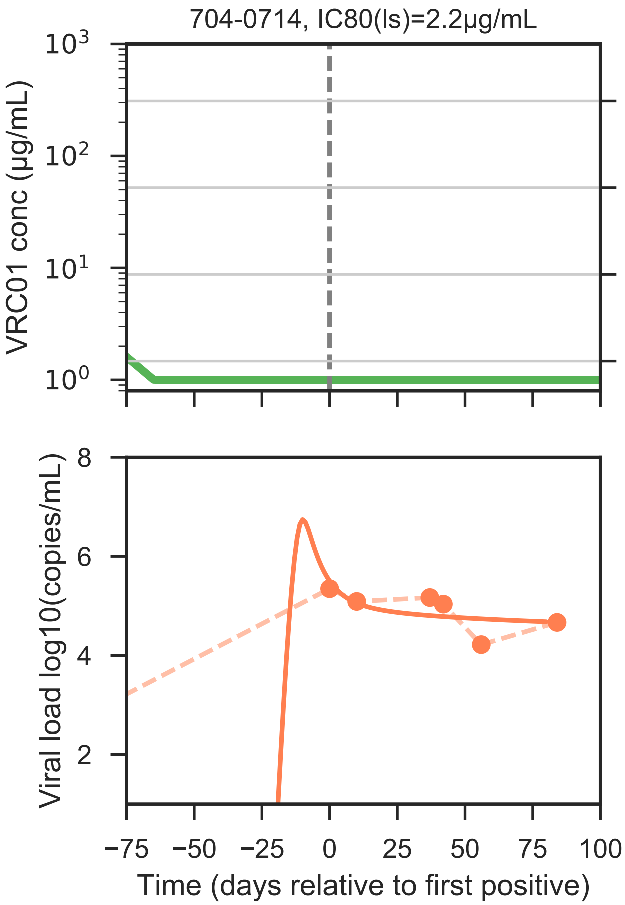
704-0714, IC80(ls)=2.2μg/mL
VRC01 conc (μg/mL)
log10 Titer
Viral load log10(copies/mL)
Time (days relative to first positive)

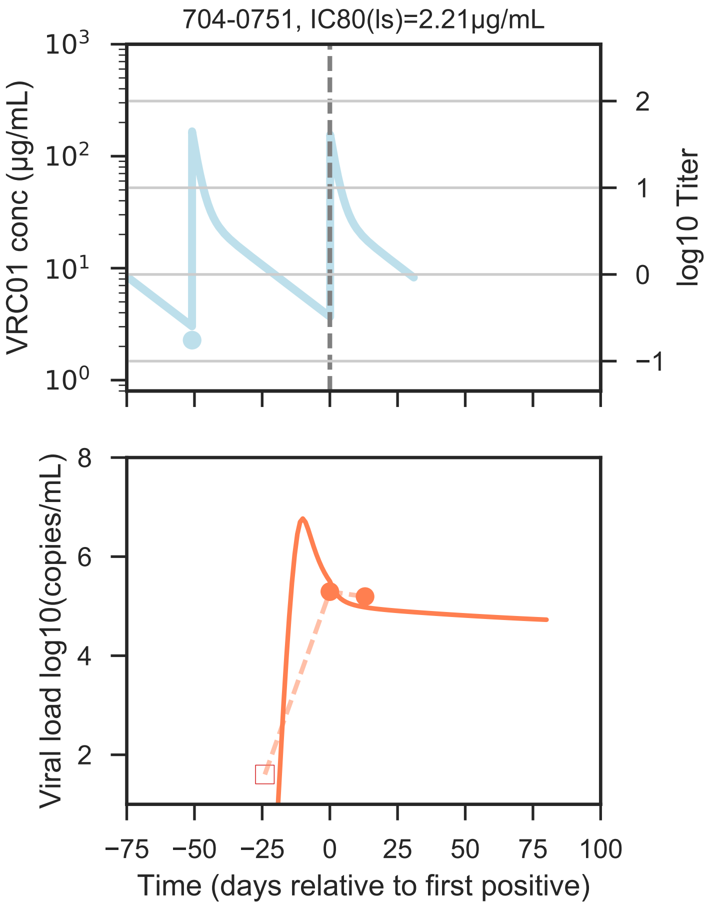

704-0751, IC80(ls)=2.21μg/mL
VRC01 conc (μg/mL)
log10 Titer
Viral load log10(copies/mL)
Time (days relative to first positive)

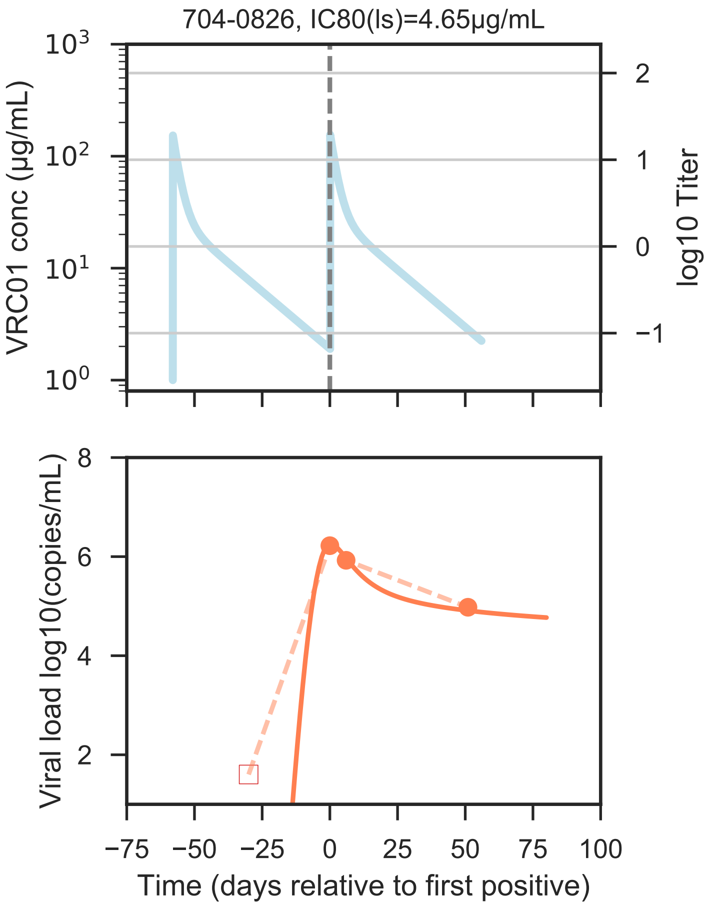

704-0826, IC80(ls)=4.65μg/mL
VRC01 conc (μg/mL)
log10 Titer
Viral load log10(copies/mL)
Time (days relative to first positive)

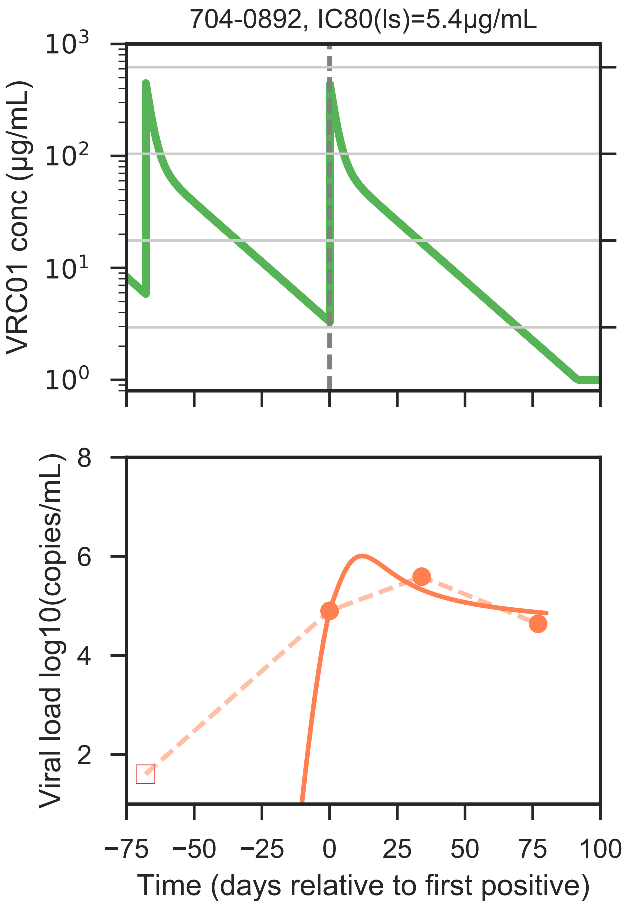

704-0892, IC80(ls)=5.4μg/mL
VRC01 conc (μg/mL)
log10 Titer
10^3
10^2
10^1
10^0
2
1
0
−1
Viral load log10(copies/mL)
8
6
4
2
−75
−50
−25
0
25
50
75
100
Time (days relative to first positive)

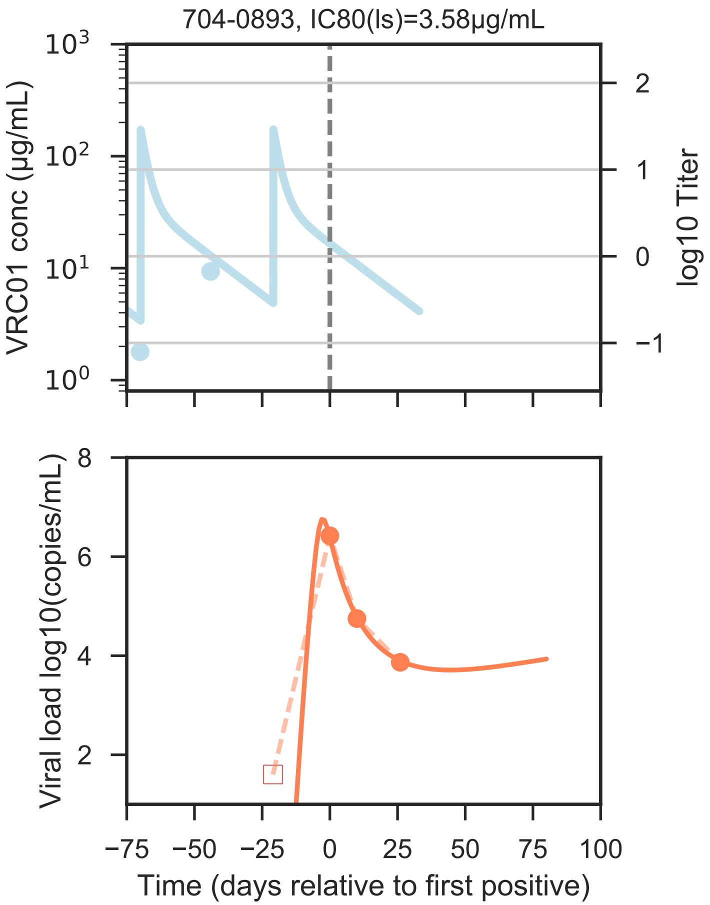

704-0893, IC80(ls)=3.58μg/mL
VRC01 conc (μg/mL)
log10 Titer
Viral load log10(copies/mL)
Time (days relative to first positive)

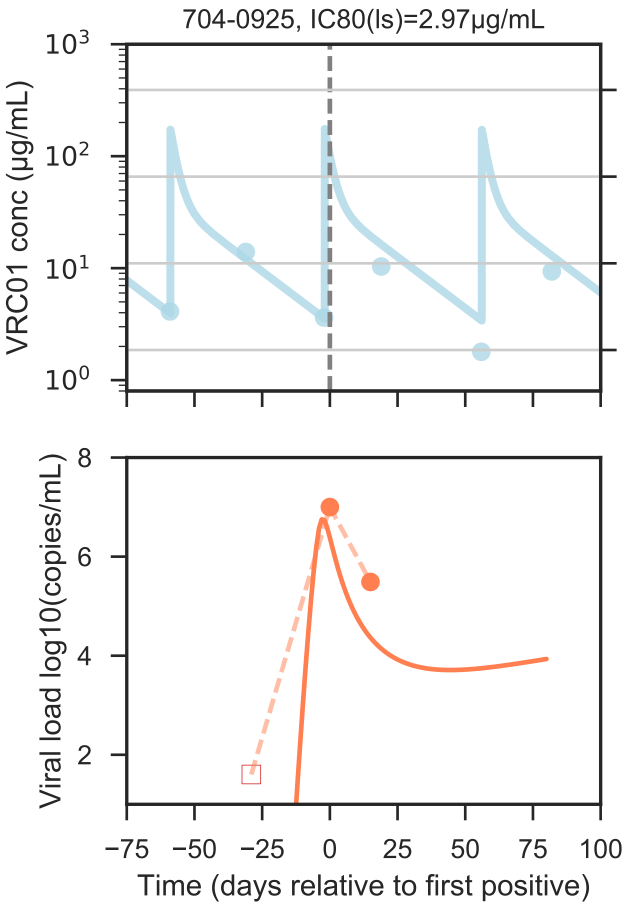
704-0925, IC80(ls)=2.97µg/mL
VRC01 conc (µg/mL)
log10 Titer
Viral load log10(copies/mL)
Time (days relative to first positive)

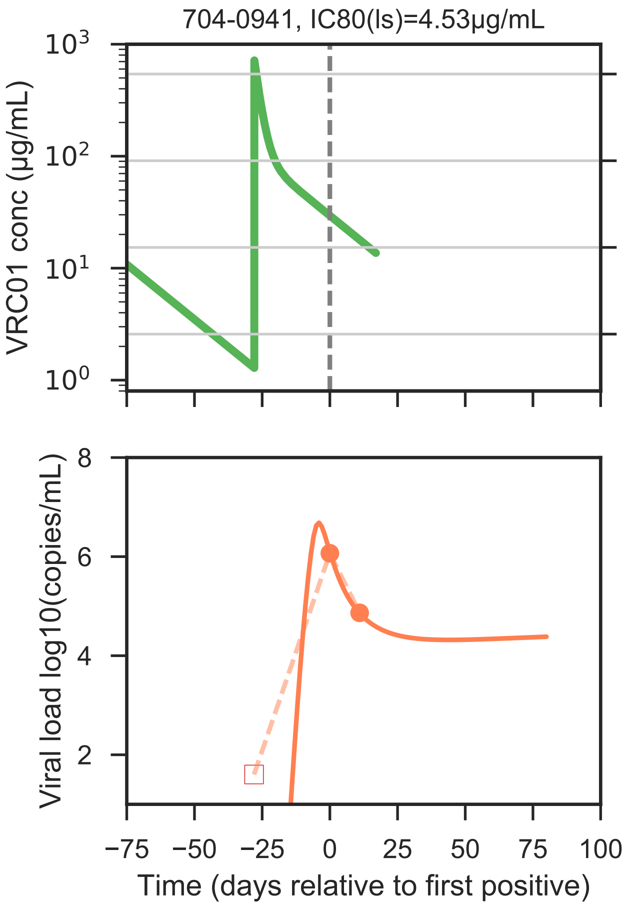

704-0941, IC80(ls)=4.53μg/mL
VRC01 conc (μg/mL)
log10 Titer
Viral load log10(copies/mL)
Time (days relative to first positive)
−75
−50
−25
0
25
50
75
100

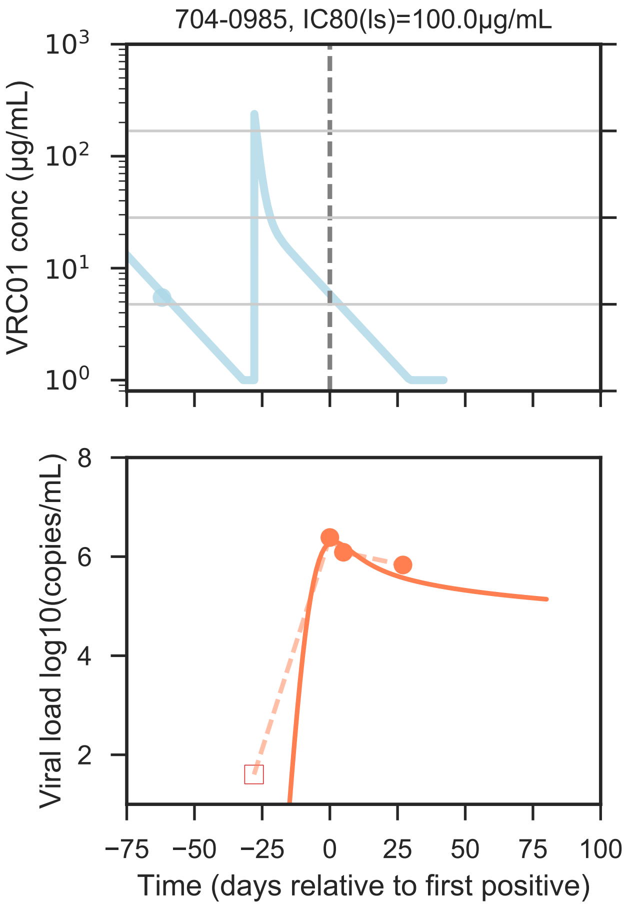
704-0985, IC80(ls)=100.0μg/mL
VRC01 conc (μg/mL)
log10 Titer
Viral load log10(copies/mL)
Time (days relative to first positive)
10^3
10^2
10^1
10^0
1
0
−1
−2
−3
8
6
4
2
−75
−50
−25
0
25
50
75
100

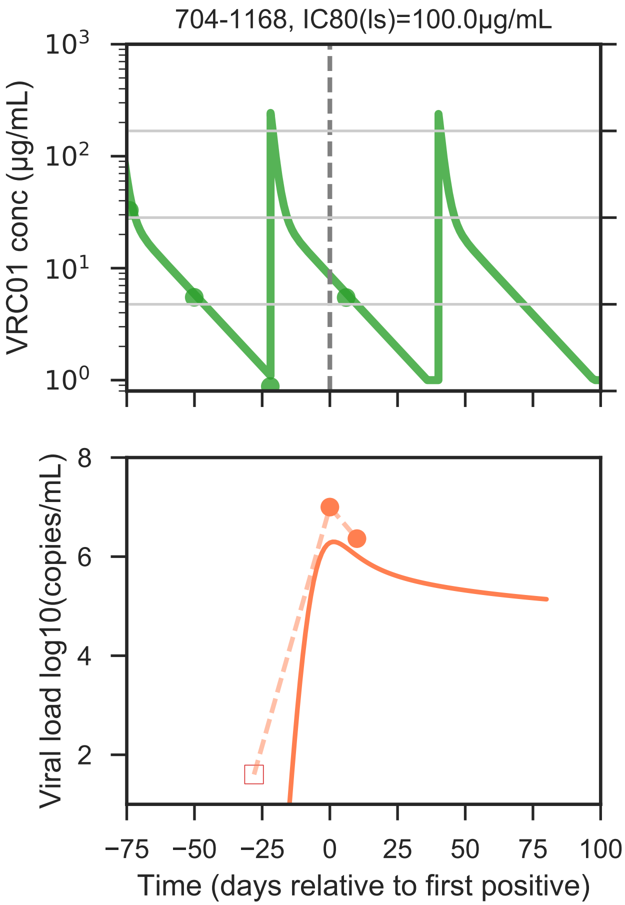

704-1168, IC80(ls)=100.0μg/mL
VRC01 conc (μg/mL)
log10 Titer
10^3
10^2
10^1
10^0
1
0
−1
−2
−3
Viral load log10(copies/mL)
8
6
4
2
−75
−50
−25
0
25
50
75
100
Time (days relative to first positive)

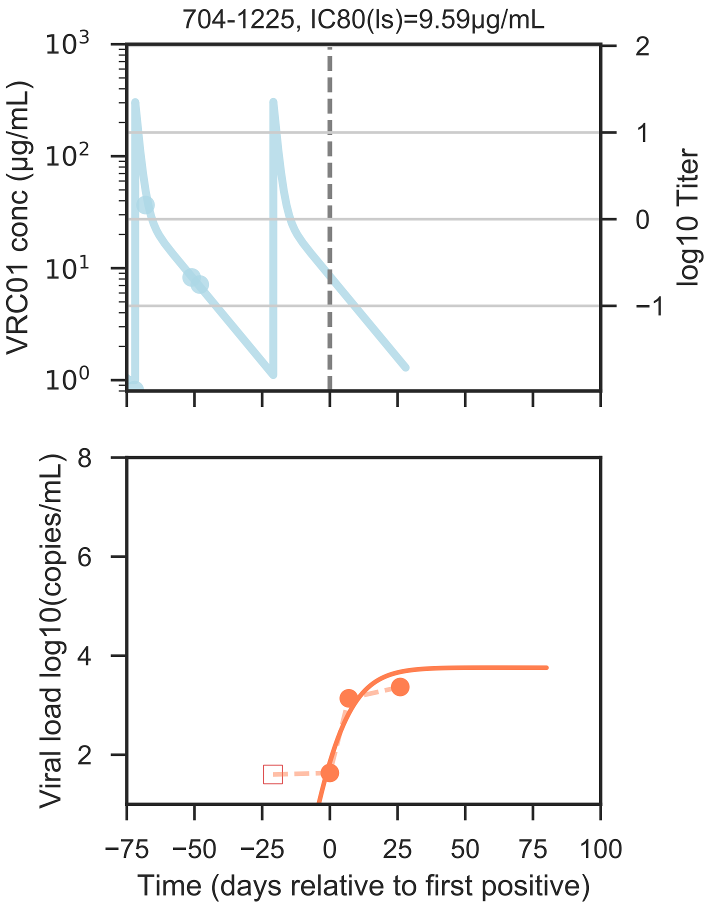

704-1225, IC80(ls)=9.59µg/mL
VRC01 conc (µg/mL)
10^3
10^2
10^1
10^0
log10 Titer
2
1
0
−1
Viral load log10(copies/mL)
8
6
4
2
−75
−50
−25
0
25
50
75
100
Time (days relative to first positive)

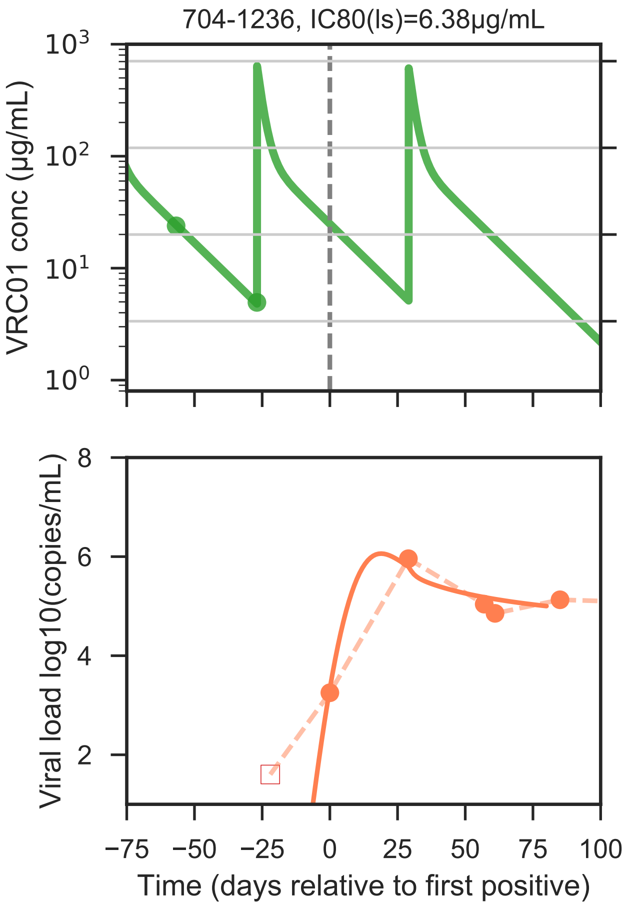
704-1236, IC80(ls)=6.38μg/mL
VRC01 conc (μg/mL)
log10 Titer
Viral load log10(copies/mL)
Time (days relative to first positive)
10^3
10^2
10^1
10^0
2
1
0
−1
8
6
4
2
−75
−50
−25
0
25
50
75
100

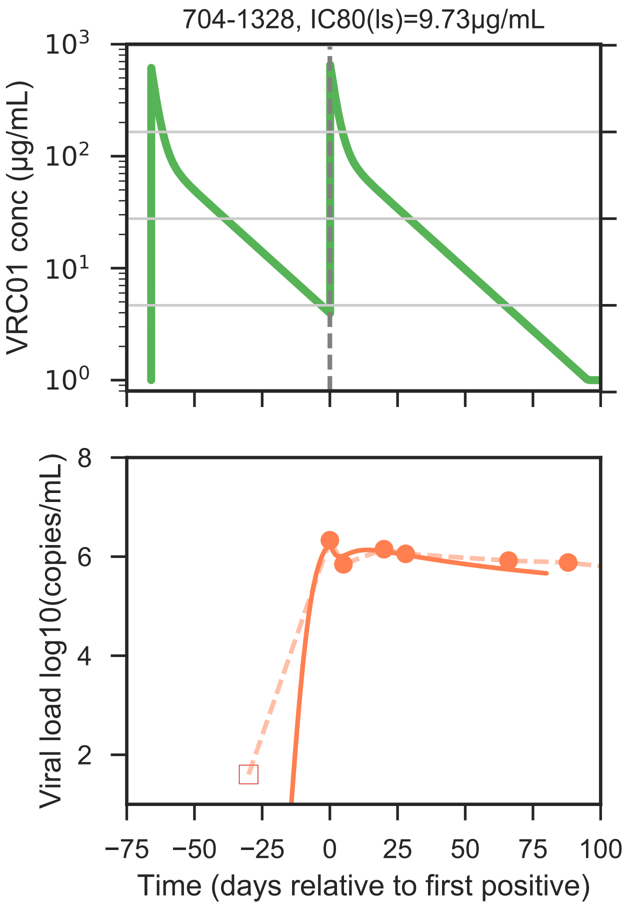

704-1328, IC80(ls)=9.73μg/mL
VRC01 conc (μg/mL)
log10 Titer
Viral load log10(copies/mL)
Time (days relative to first positive)

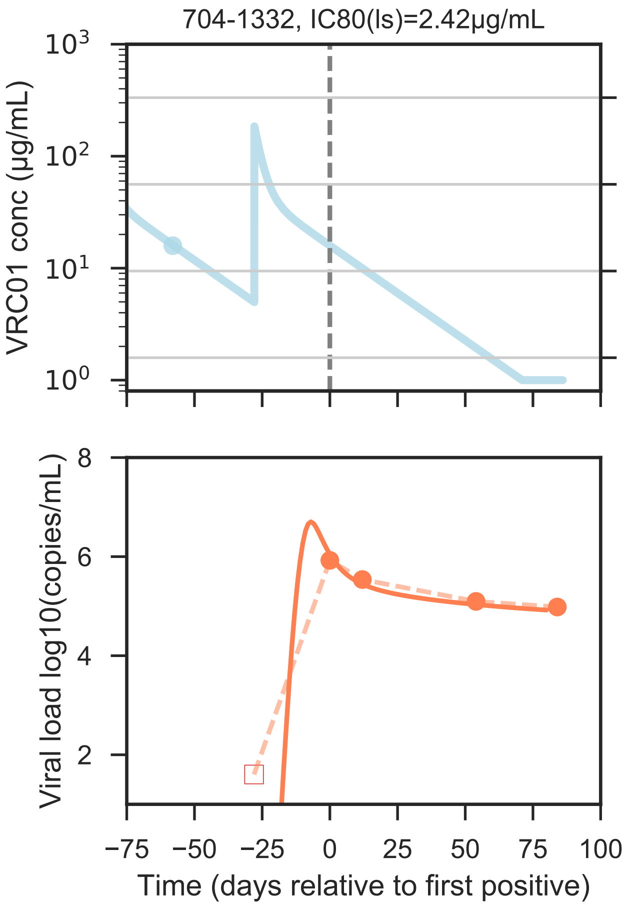

704-1332, IC80(ls)=2.42μg/mL
VRC01 conc (μg/mL)
log10 Titer
Viral load log10(copies/mL)
Time (days relative to first positive)

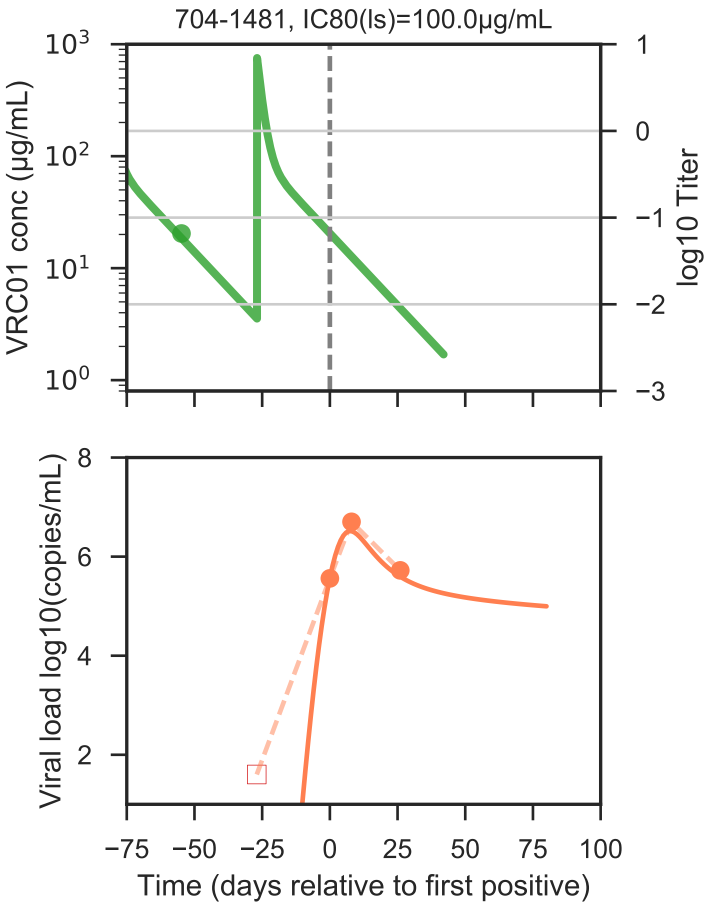

704-1481, IC80(ls)=100.0μg/mL
VRC01 conc (μg/mL)
log10 Titer
Viral load log10(copies/mL)
Time (days relative to first positive)

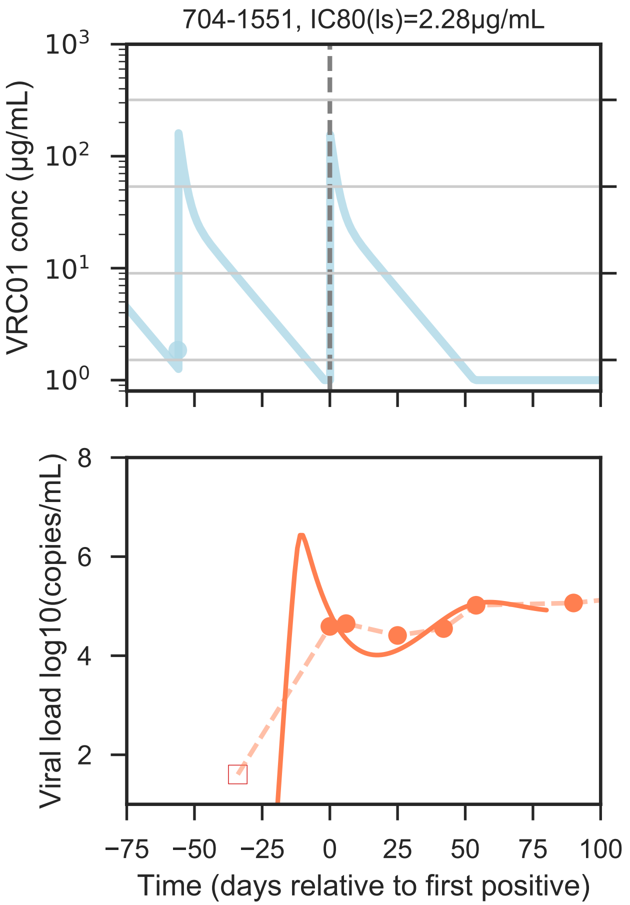

704-1551, IC80(ls)=2.28μg/mL
VRC01 conc (μg/mL)
log10 Titer
Viral load log10(copies/mL)
Time (days relative to first positive)

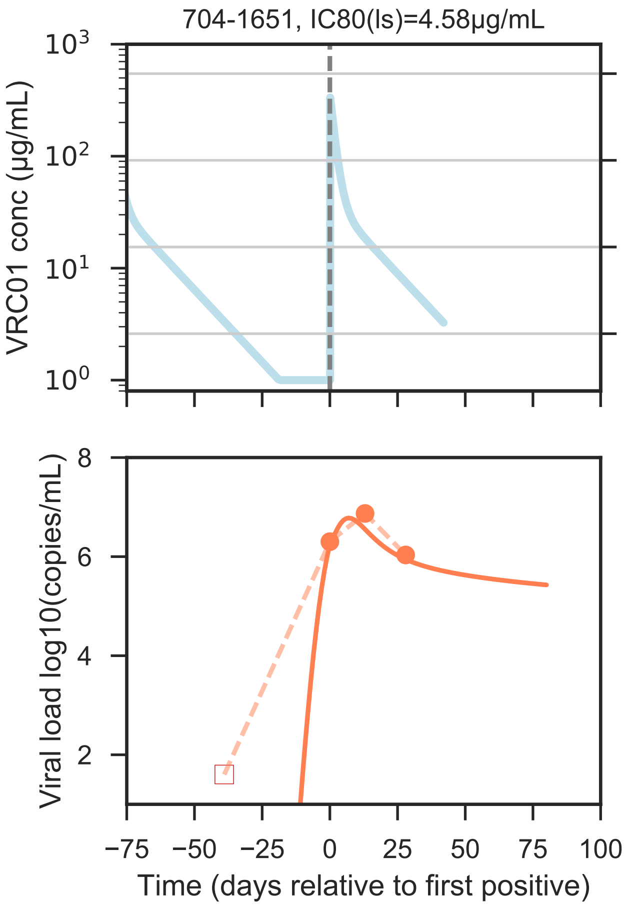
704-1651, IC80(ls)=4.58μg/mL
VRC01 conc (μg/mL)
log10 Titer
Viral load log10(copies/mL)
Time (days relative to first positive)
−75
−50
−25
0
25
50
75
100

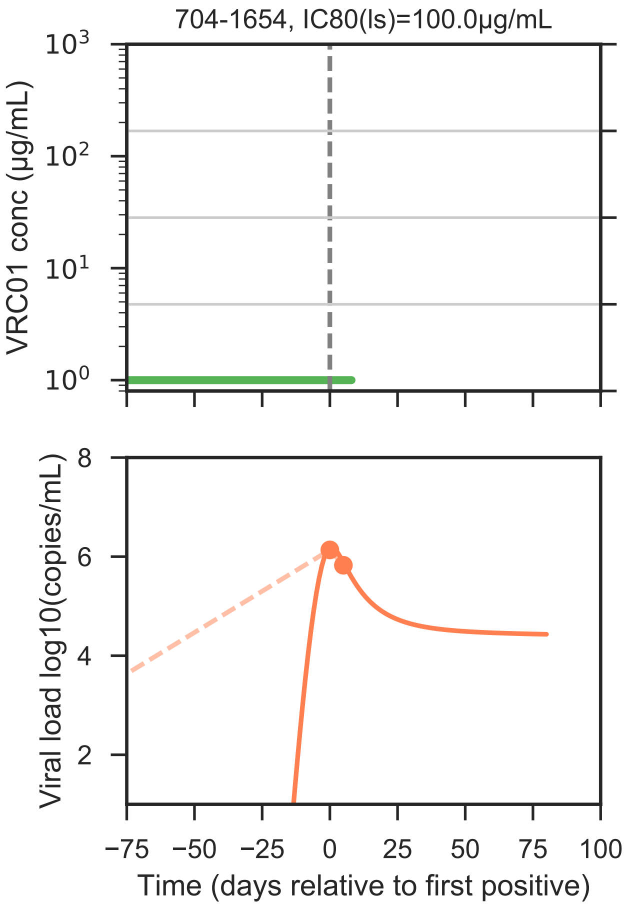

704-1654, IC80(ls)=100.0μg/mL
VRC01 conc (μg/mL)
log10 Titer
Viral load log10(copies/mL)
Time (days relative to first positive)

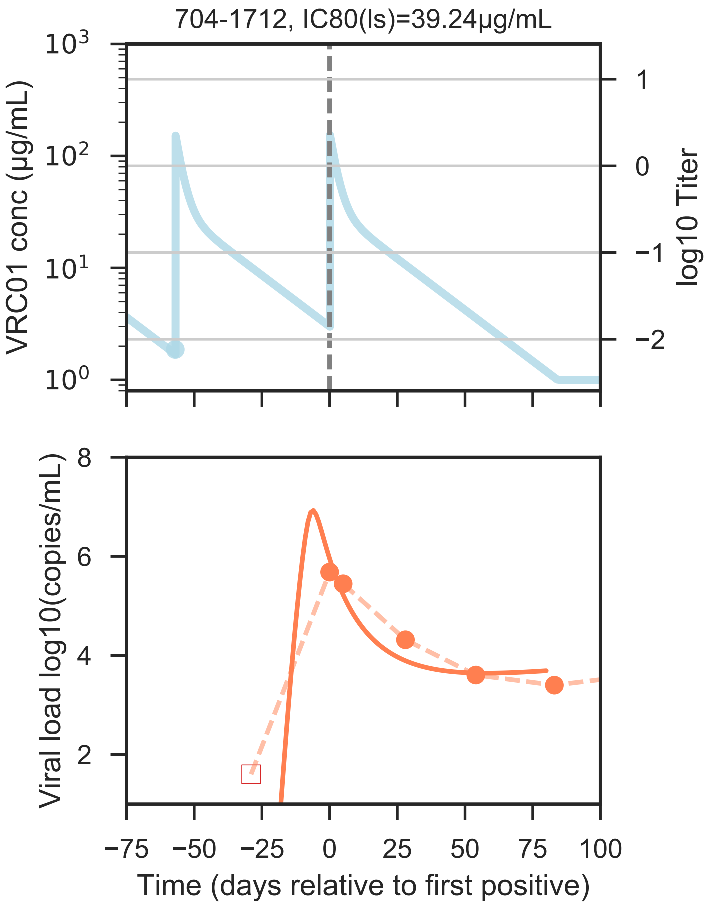
704-1712, IC80(ls)=39.24μg/mL
VRC01 conc (μg/mL)
log10 Titer
Viral load log10(copies/mL)
Time (days relative to first positive)
10^3
10^2
10^1
10^0
1
0
−1
−2
8
6
4
2
−75
−50
−25
0
25
50
75
100

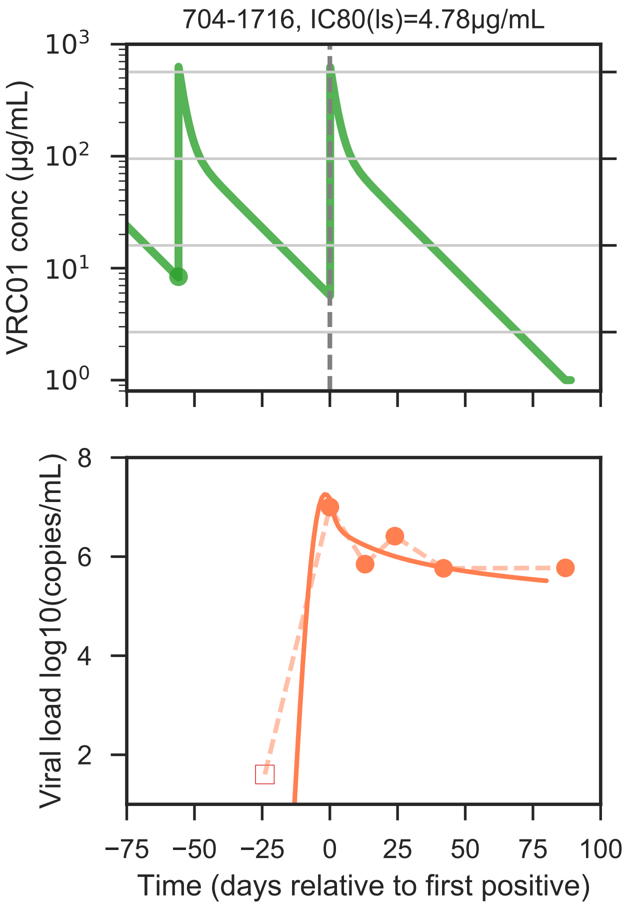

704-1716, IC80(ls)=4.78μg/mL
VRC01 conc (μg/mL)
log10 Titer
10^3
10^2
10^1
10^0
2
1
0
−1
Viral load log10(copies/mL)
8
6
4
2
−75
−50
−25
0
25
50
75
100
Time (days relative to first positive)

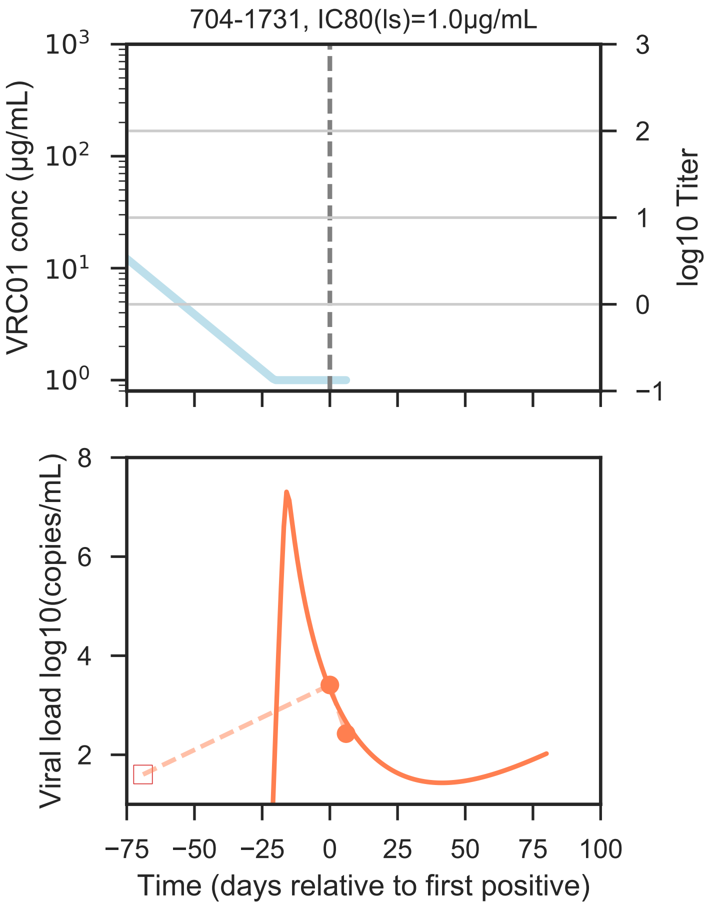
704-1731, IC80(ls)=1.0μg/mL
VRC01 conc (μg/mL)
log10 Titer
10^3
10^2
10^1
10^0
3
2
1
0
−1
Viral load log10(copies/mL)
8
6
4
2
−75
−50
−25
0
25
50
75
100
Time (days relative to first positive)

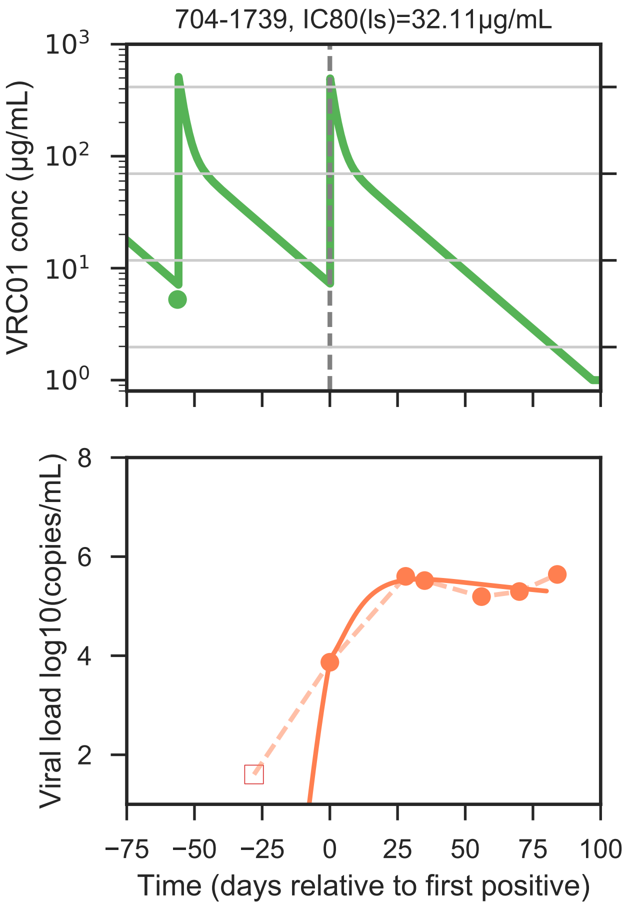

704-1739, IC80(ls)=32.11μg/mL
VRC01 conc (μg/mL)
log10 Titer
Viral load log10(copies/mL)
Time (days relative to first positive)

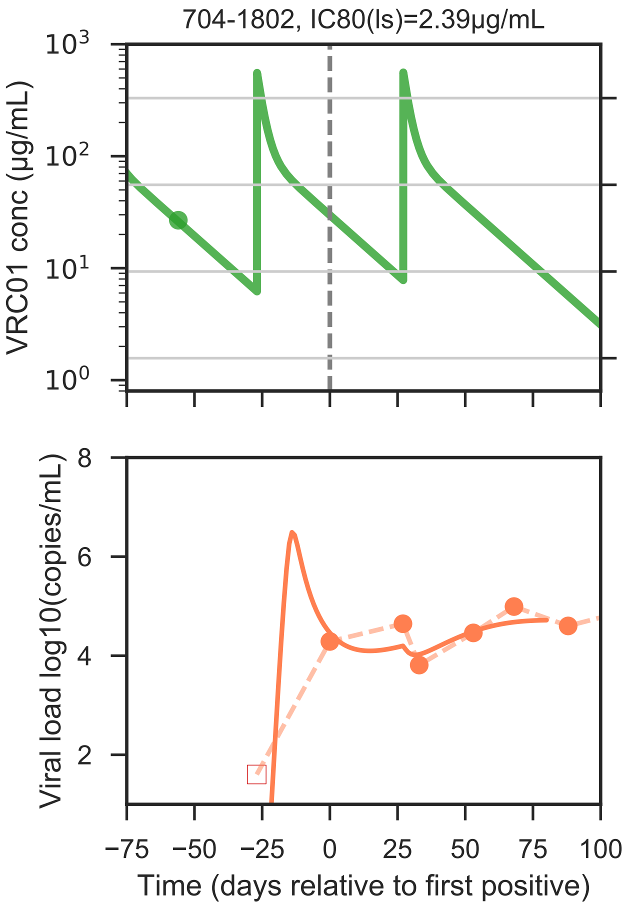

704-1802, IC80(ls)=2.39μg/mL
VRC01 conc (μg/mL)
log10 Titer
Viral load log10(copies/mL)
Time (days relative to first positive)

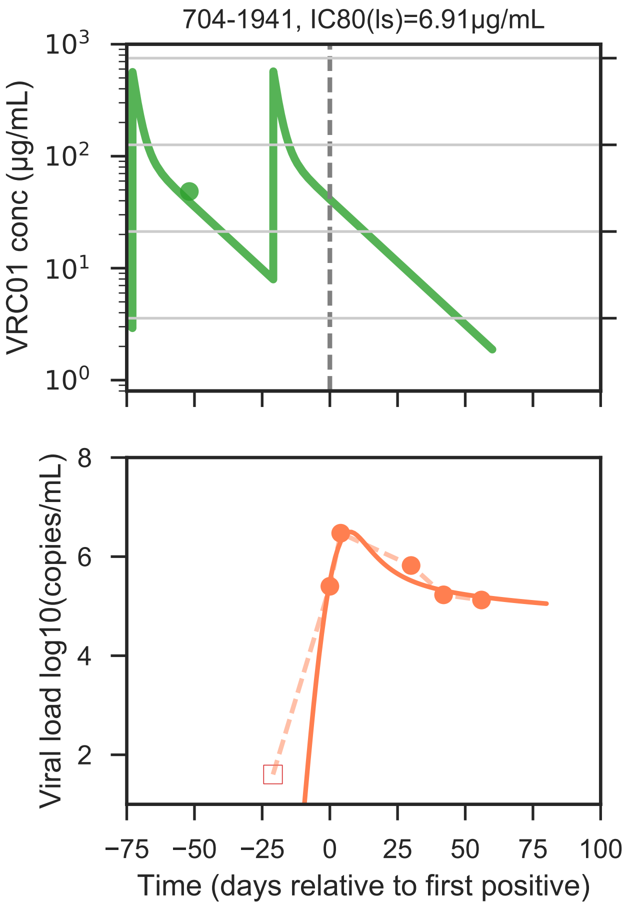

704-1941, IC80(ls)=6.91μg/mL
VRC01 conc (μg/mL)
log10 Titer
Viral load log10(copies/mL)
Time (days relative to first positive)

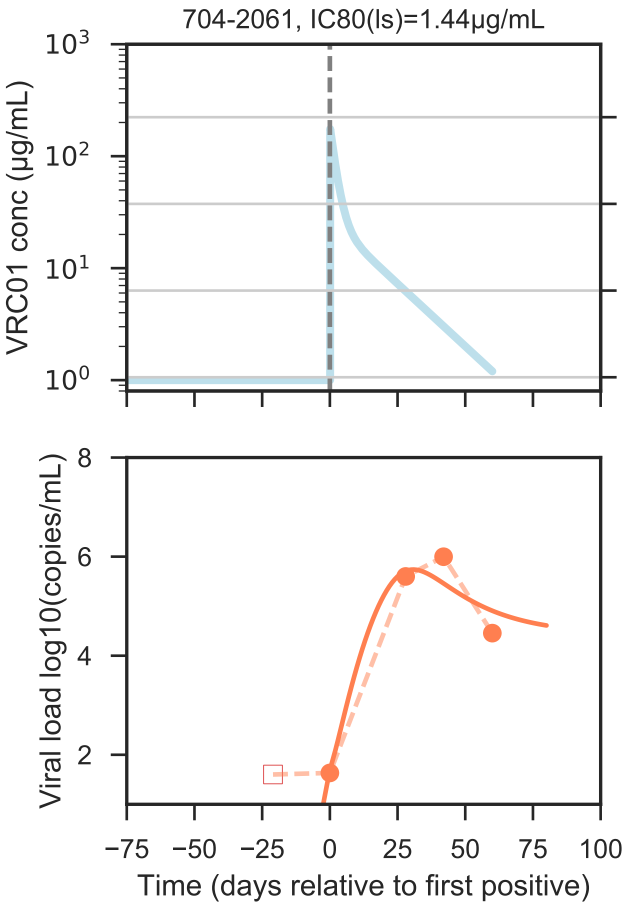

704-2061, IC80(ls)=1.44μg/mL
VRC01 conc (μg/mL)
log10 Titer
Viral load log10(copies/mL)
Time (days relative to first positive)

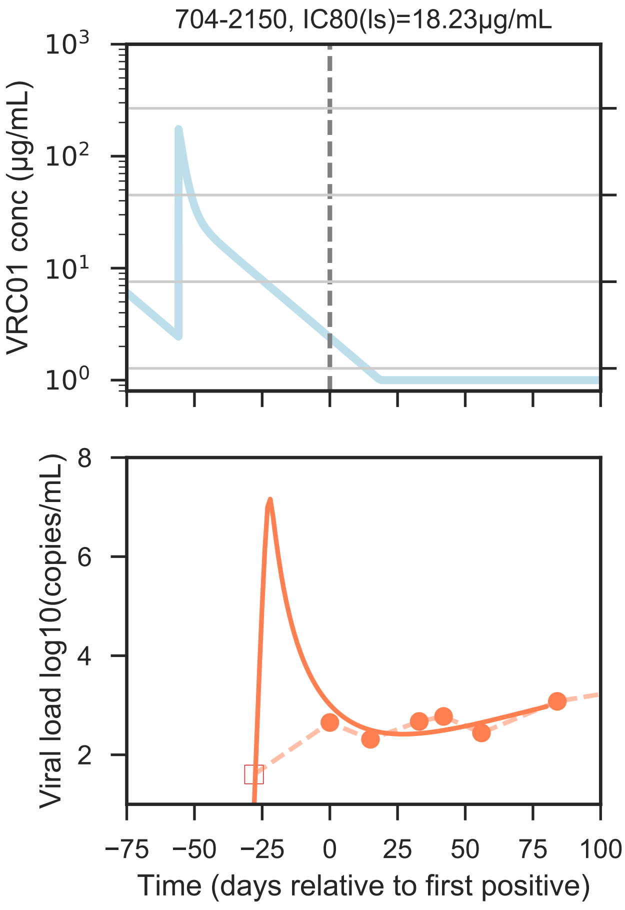
704-2150, IC80(ls)=18.23μg/mL
VRC01 conc (μg/mL)
log10 Titer
Viral load log10(copies/mL)
Time (days relative to first positive)

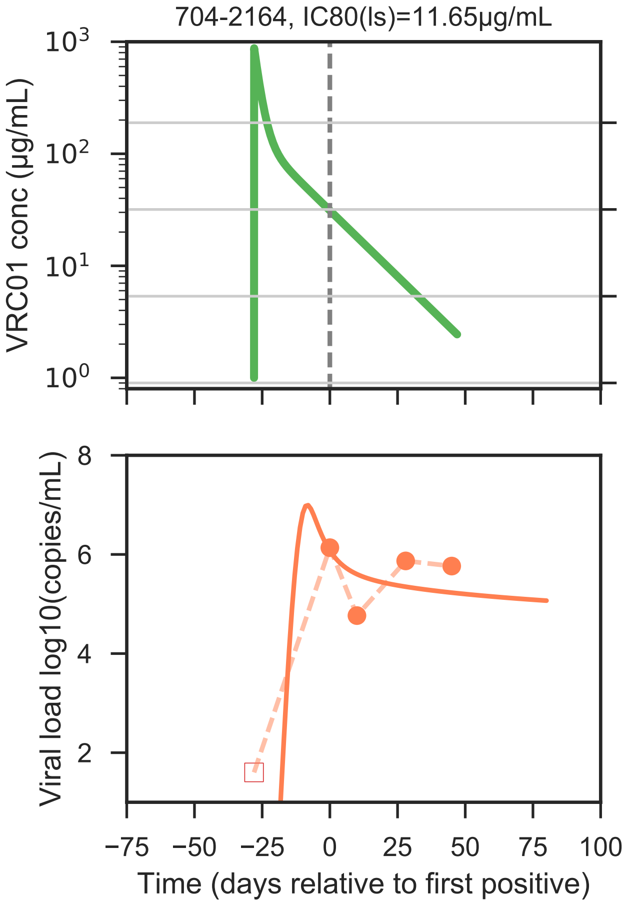

704-2164, IC80(ls)=11.65μg/mL
VRC01 conc (μg/mL)
log10 Titer
10^3
10^2
10^1
10^0
1
0
−1
−2
Viral load log10(copies/mL)
8
6
4
2
−75
−50
−25
0
25
50
75
100
Time (days relative to first positive)

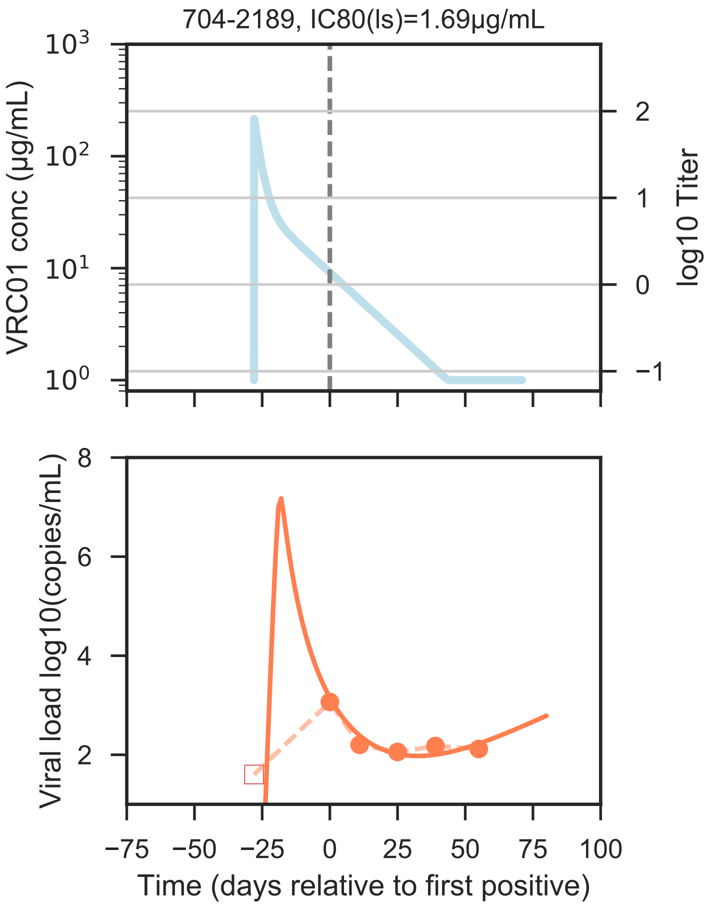

704-2189, IC80(ls)=1.69µg/mL
VRC01 conc (µg/mL)
log10 Titer
Viral load log10(copies/mL)
Time (days relative to first positive)

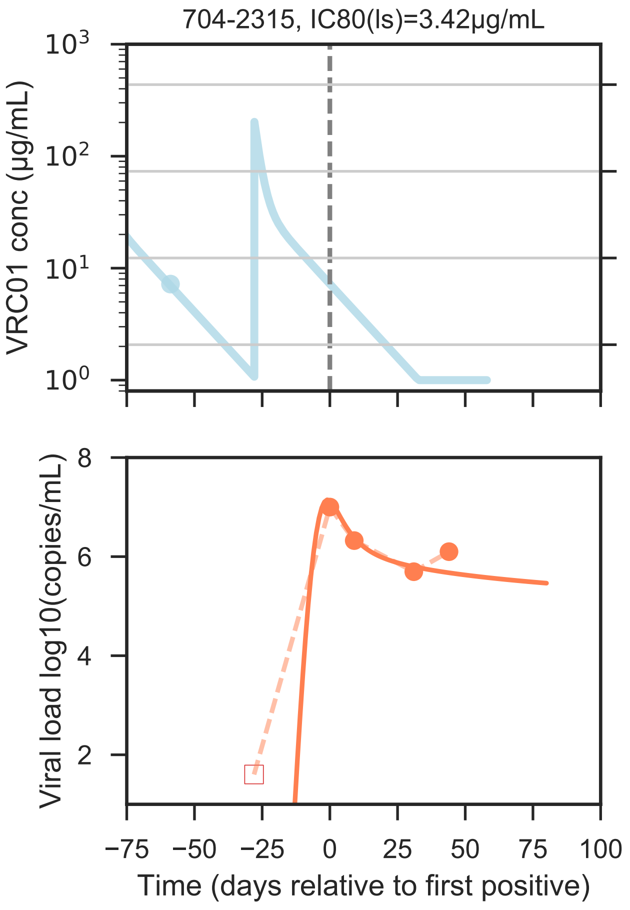

704-2315, IC80(ls)=3.42μg/mL
VRC01 conc (μg/mL)
log10 Titer
Viral load log10(copies/mL)
Time (days relative to first positive)

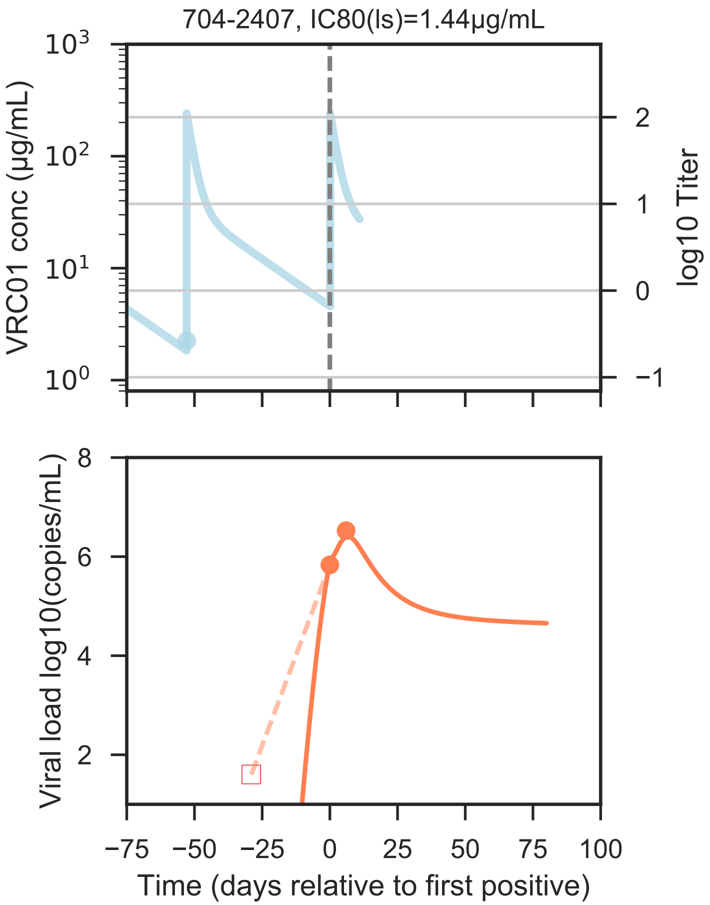

704-2407, IC80(ls)=1.44μg/mL
VRC01 conc (μg/mL)
log10 Titer
Viral load log10(copies/mL)
Time (days relative to first positive)

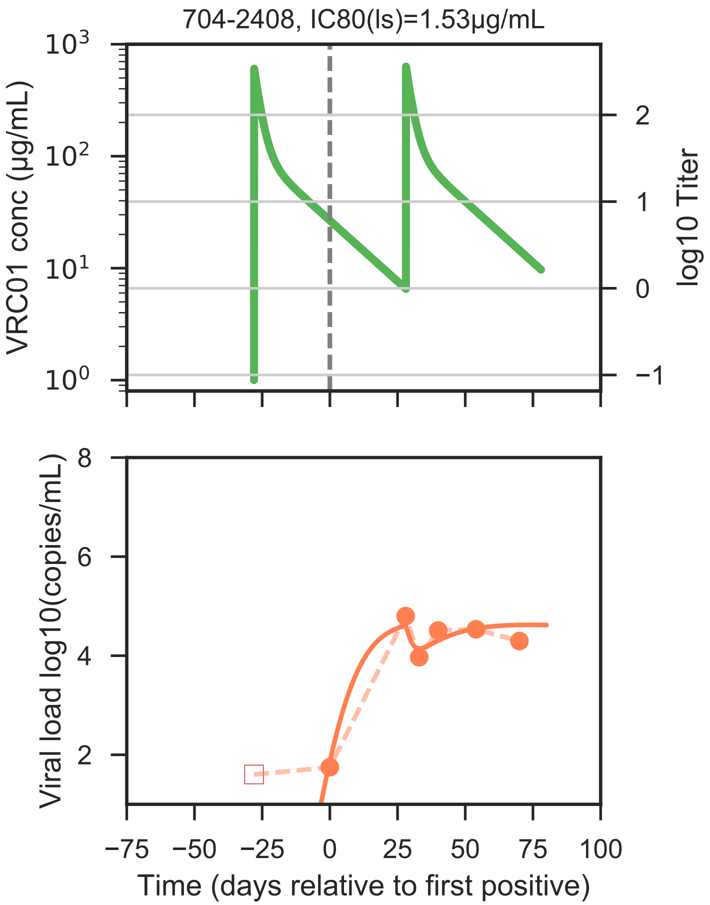

704-2408, IC80(ls)=1.53μg/mL
VRC01 conc (μg/mL)
10^3
10^2
10^1
10^0
log10 Titer
2
1
0
−1
Viral load log10(copies/mL)
8
6
4
2
−75
−50
−25
0
25
50
75
100
Time (days relative to first positive)

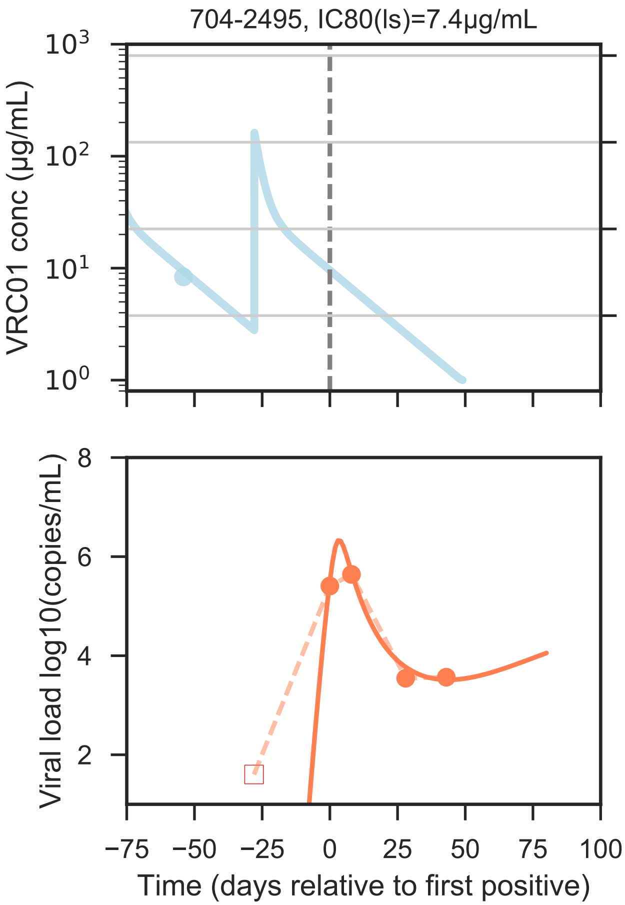
704-2495, IC80(ls)=7.4μg/mL
VRC01 conc (μg/mL)
log10 Titer
Viral load log10(copies/mL)
Time (days relative to first positive)

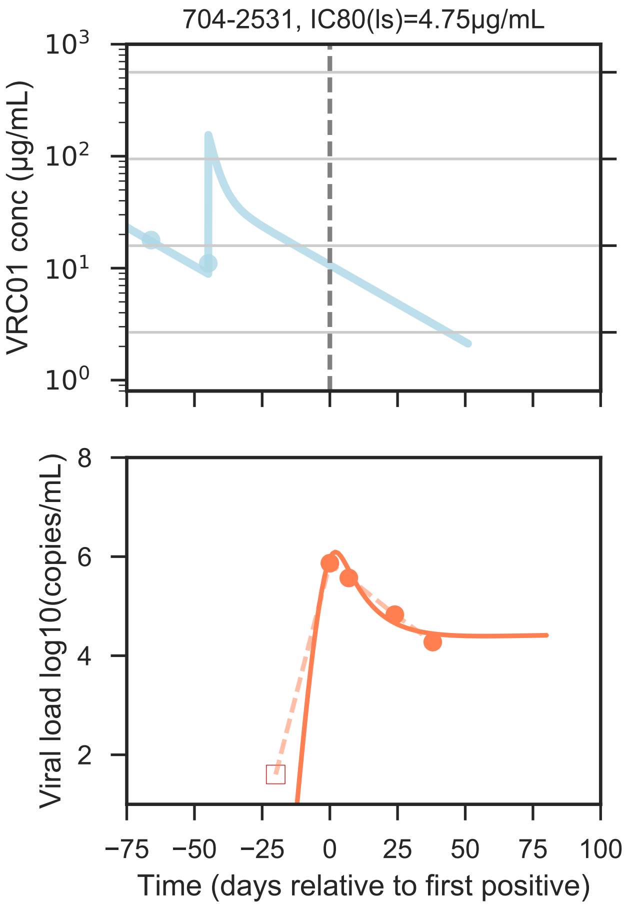

704-2531, IC80(ls)=4.75μg/mL
VRC01 conc (μg/mL)
log10 Titer
Viral load log10(copies/mL)
Time (days relative to first positive)

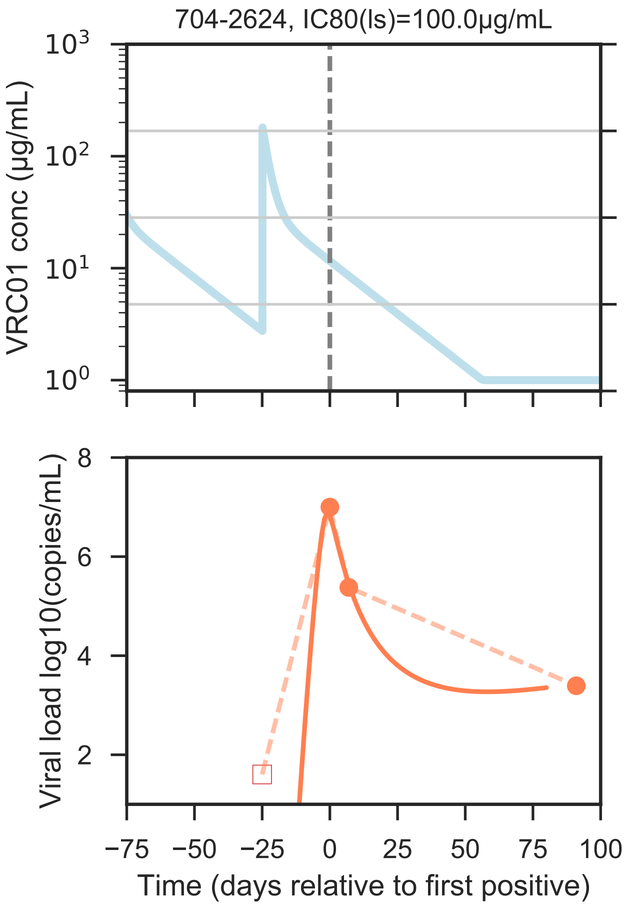
704-2624, IC80(ls)=100.0μg/mL
VRC01 conc (μg/mL)
log10 Titer
Viral load log10(copies/mL)
Time (days relative to first positive)

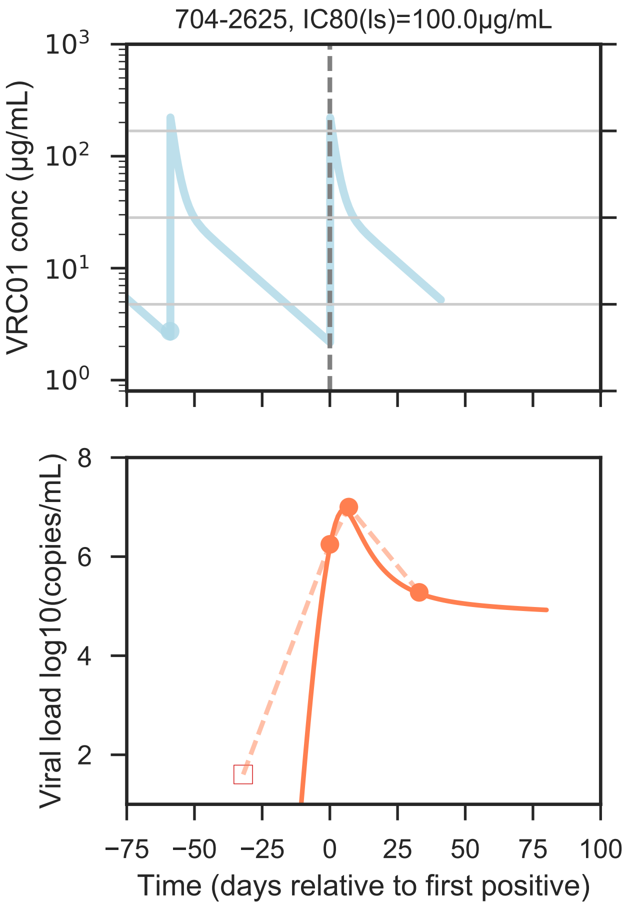

704-2625, IC80(ls)=100.0μg/mL
VRC01 conc (μg/mL)
log10 Titer
Viral load log10(copies/mL)
Time (days relative to first positive)

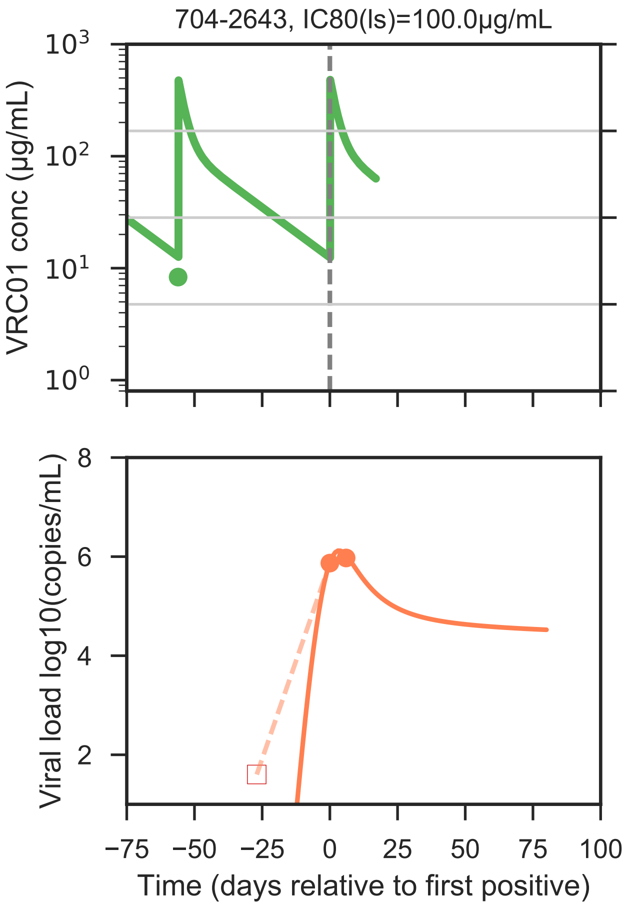

704-2643, IC80(ls)=100.0μg/mL
VRC01 conc (μg/mL)
log10 Titer
Viral load log10(copies/mL)
Time (days relative to first positive)

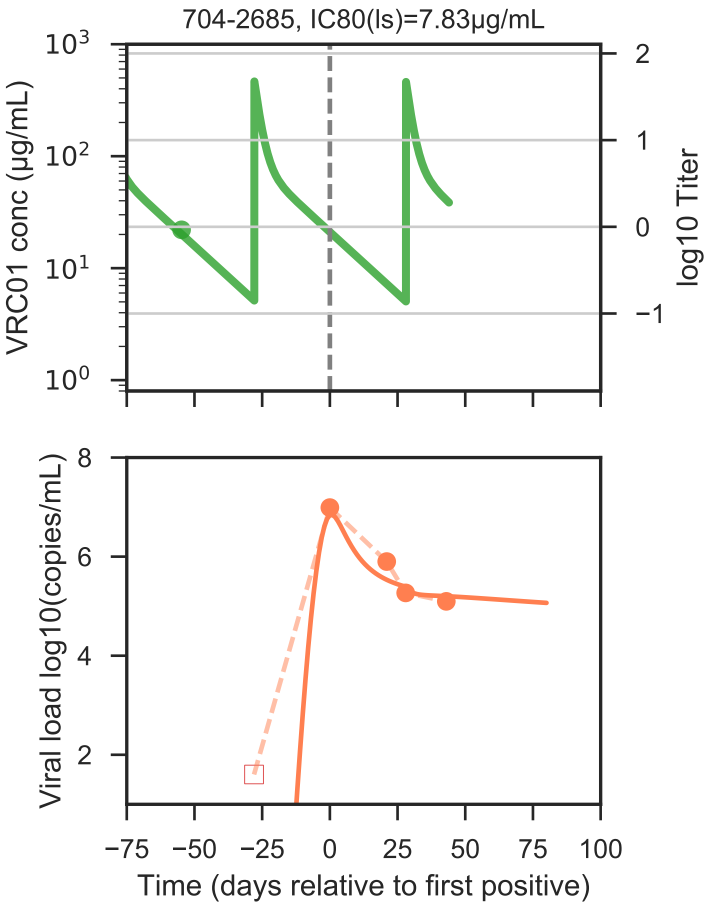

704-2685, IC80(ls)=7.83μg/mL
VRC01 conc (μg/mL)
log10 Titer
10^3
10^2
10^1
10^0
2
1
0
−1
Viral load log10(copies/mL)
8
6
4
2
−75 −50 −25 0 25 50 75 100
Time (days relative to first positive)

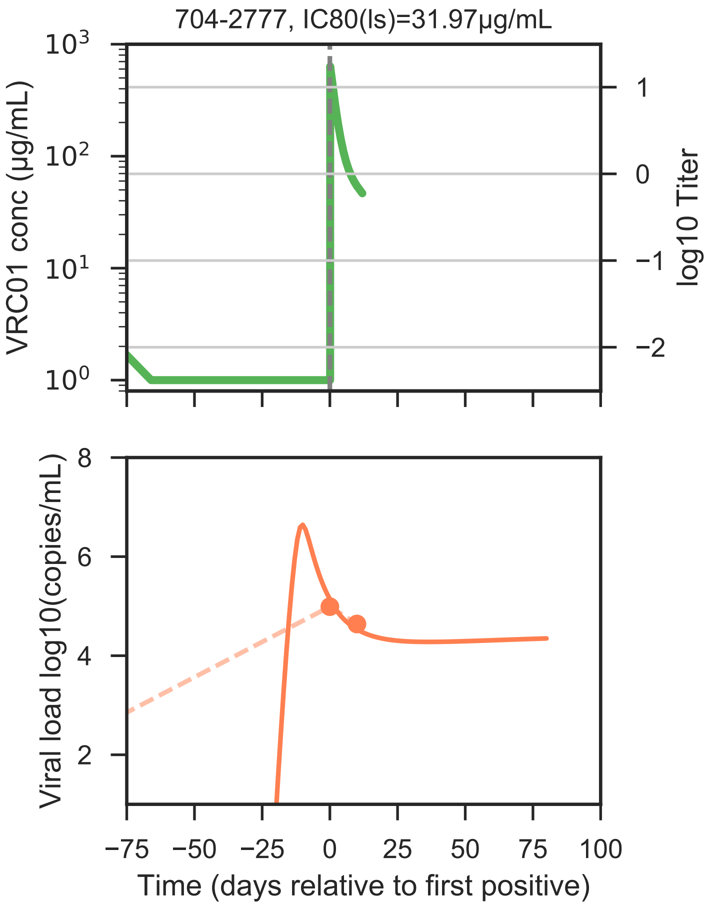
704-2777, IC80(ls)=31.97μg/mL
VRC01 conc (μg/mL)
log10 Titer
Viral load log10(copies/mL)
Time (days relative to first positive)
10^3
10^2
10^1
10^0
1
0
−1
−2
8
6
4
2
−75
−50
−25
0
25
50
75
100

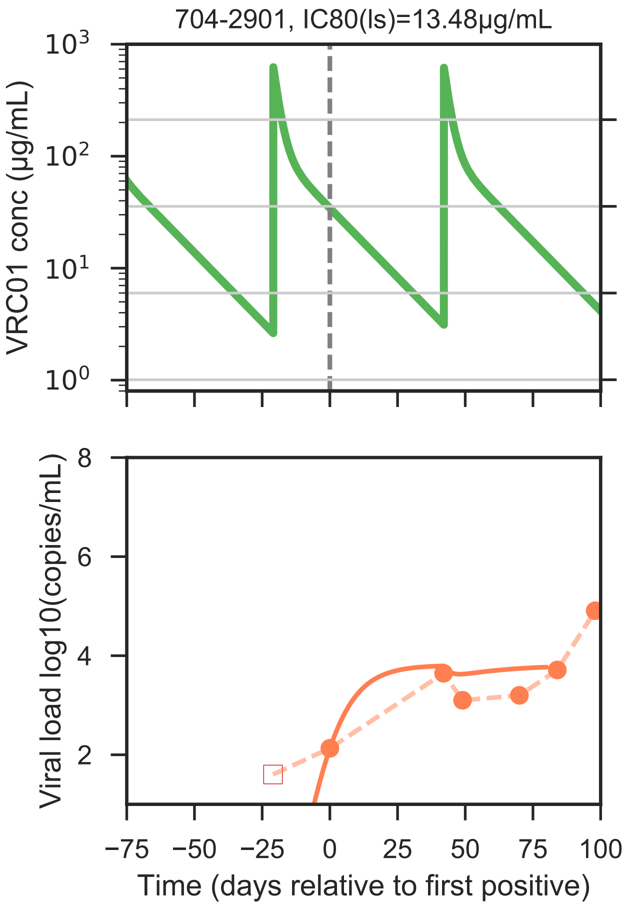
704-2901, IC80(ls)=13.48μg/mL
VRC01 conc (μg/mL)
log10 Titer
Viral load log10(copies/mL)
Time (days relative to first positive)

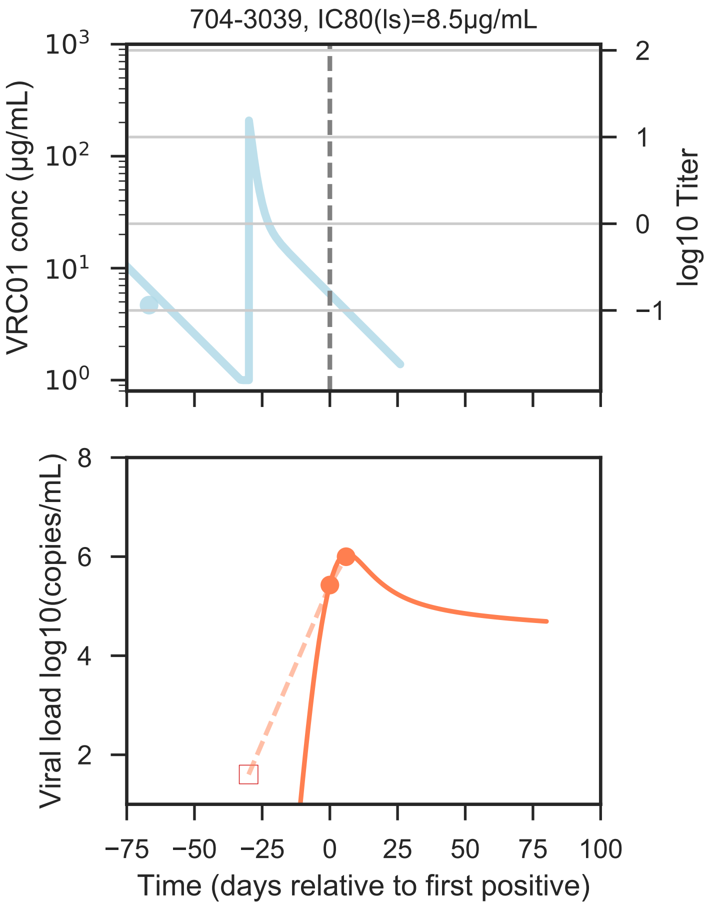
704-3039, IC80(ls)=8.5μg/mL
VRC01 conc (μg/mL)
log10 Titer
Viral load log10(copies/mL)
Time (days relative to first positive)

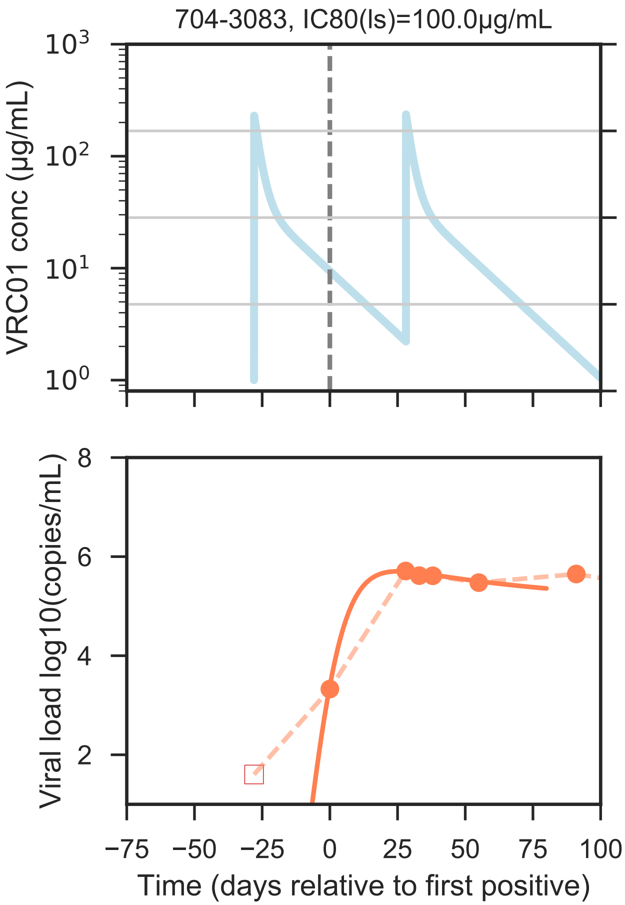
704-3083, IC80(ls)=100.0µg/mL
VRC01 conc (µg/mL)
10^3
10^2
10^1
10^0
log10 Titer
1
0
−1
−2
−3
Viral load log10(copies/mL)
8
6
4
2
−75
−50
−25
0
25
50
75
100
Time (days relative to first positive)

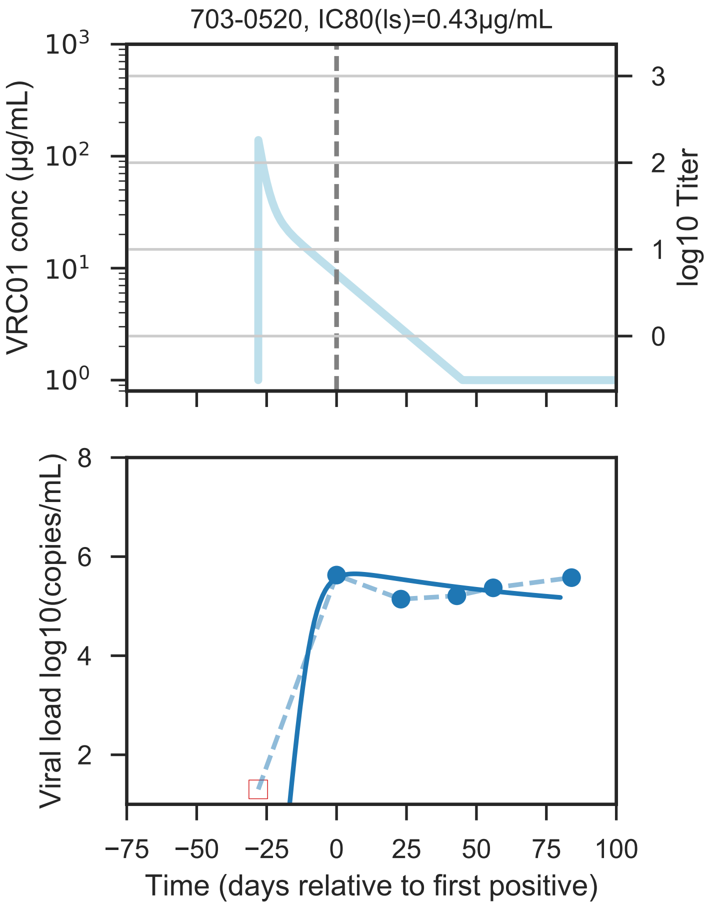

703-0520, IC80(ls)=0.43μg/mL
VRC01 conc (μg/mL)
log10 Titer
Viral load log10(copies/mL)
Time (days relative to first positive)

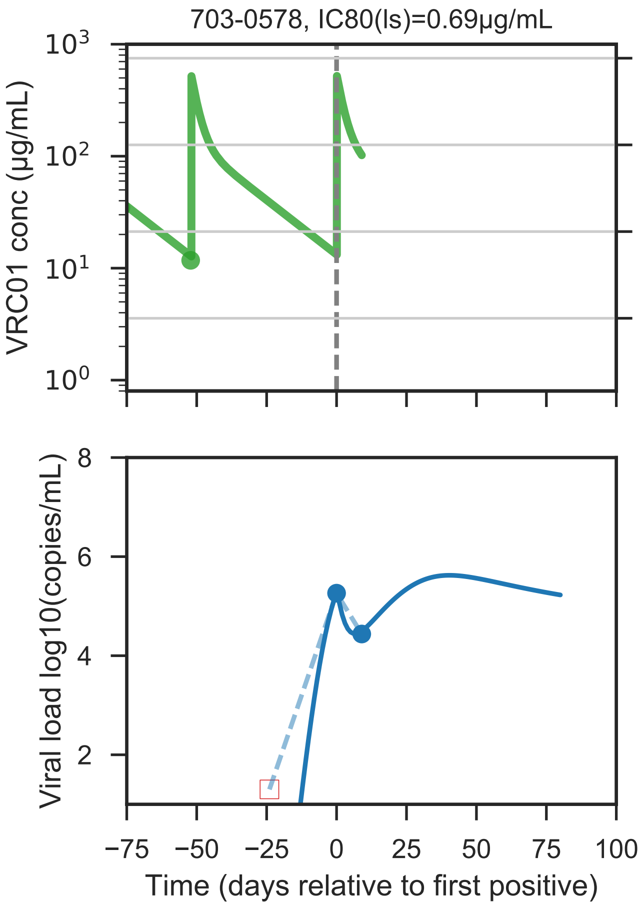
703-0578, IC80(ls)=0.69μg/mL
VRC01 conc (μg/mL)
log10 Titer
Viral load log10(copies/mL)
Time (days relative to first positive)
10^3
10^2
10^1
10^0
3
2
1
0
8
6
4
2
−75
−50
−25
0
25
50
75
100

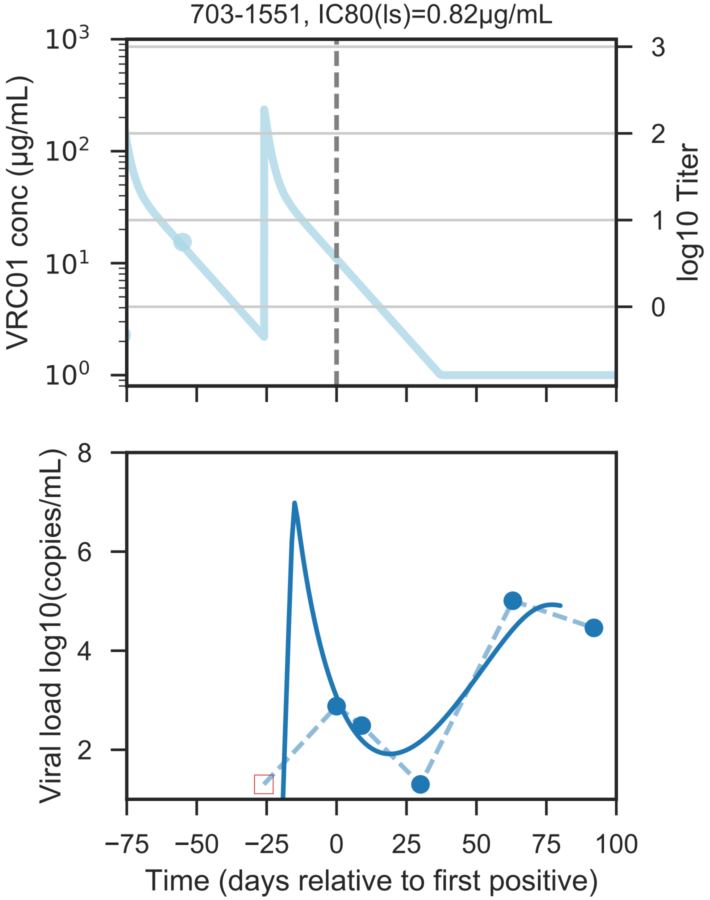
703-1551, IC80(ls)=0.82μg/mL
VRC01 conc (μg/mL)
log10 Titer
Viral load log10(copies/mL)
Time (days relative to first positive)
10^3
10^2
10^1
10^0
3
2
1
0
8
6
4
2
−75
−50
−25
0
25
50
75
100

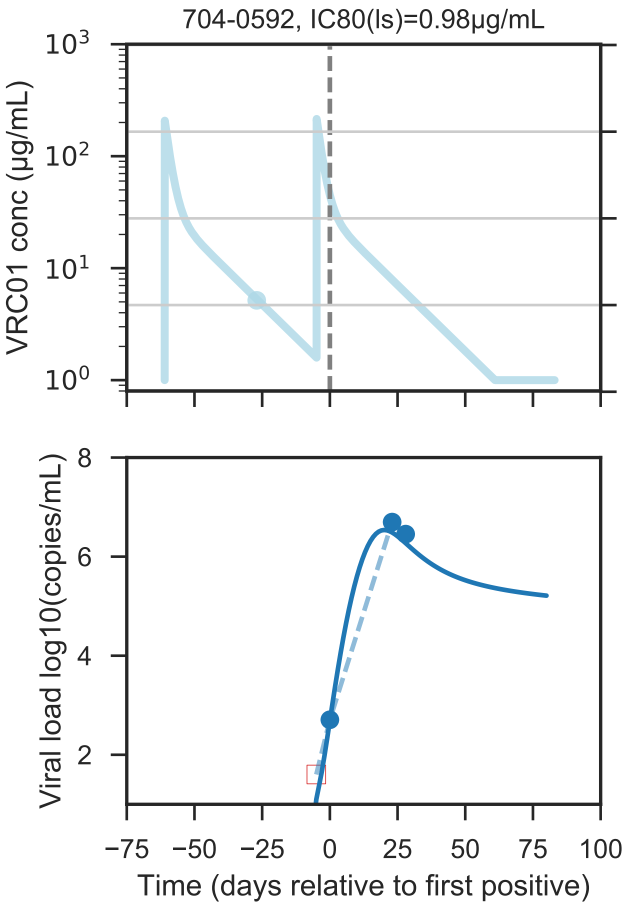

704-0592, IC80(ls)=0.98μg/mL
VRC01 conc (μg/mL)
log10 Titer
Viral load log10(copies/mL)
Time (days relative to first positive)

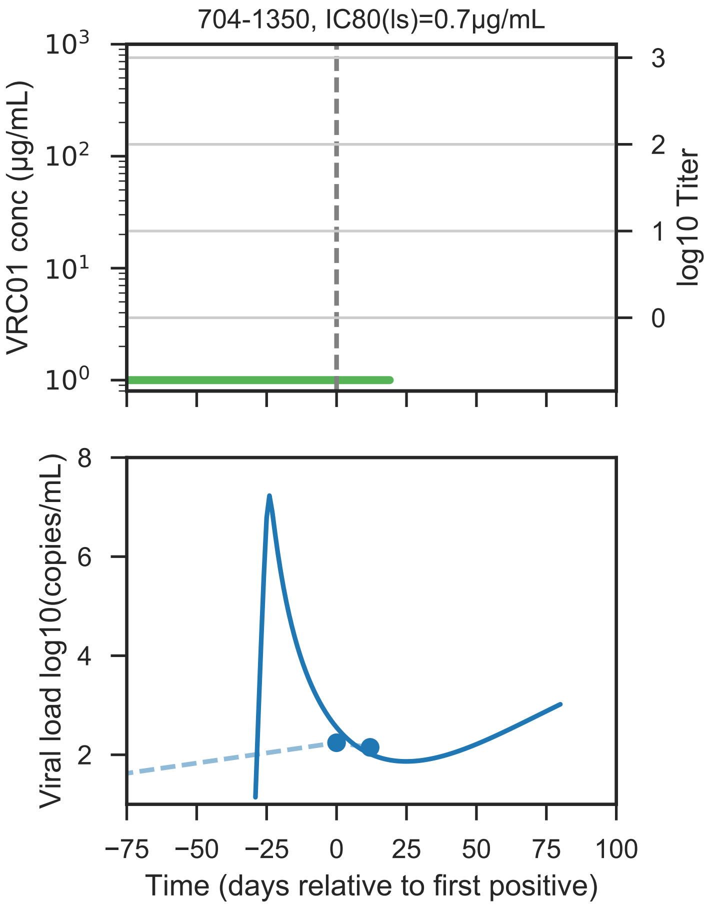
704-1350, IC80(ls)=0.7μg/mL
VRC01 conc (μg/mL)
10^3
10^2
10^1
10^0
log10 Titer
3
2
1
0
Viral load log10(copies/mL)
8
6
4
2
−75
−50
−25
0
25
50
75
100
Time (days relative to first positive)

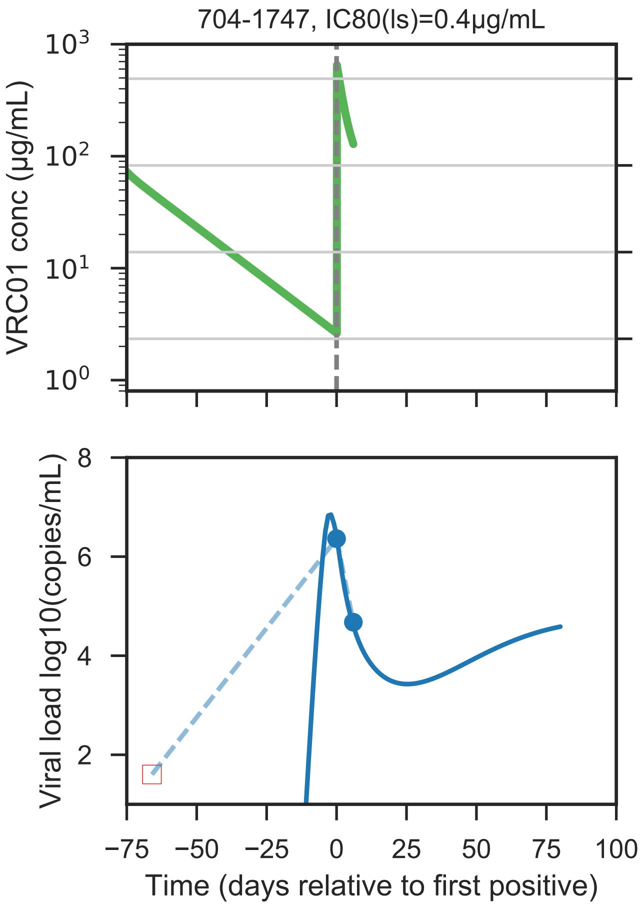

704-1747, IC80(ls)=0.4μg/mL
VRC01 conc (μg/mL)
log10 Titer
Viral load log10(copies/mL)
Time (days relative to first positive)

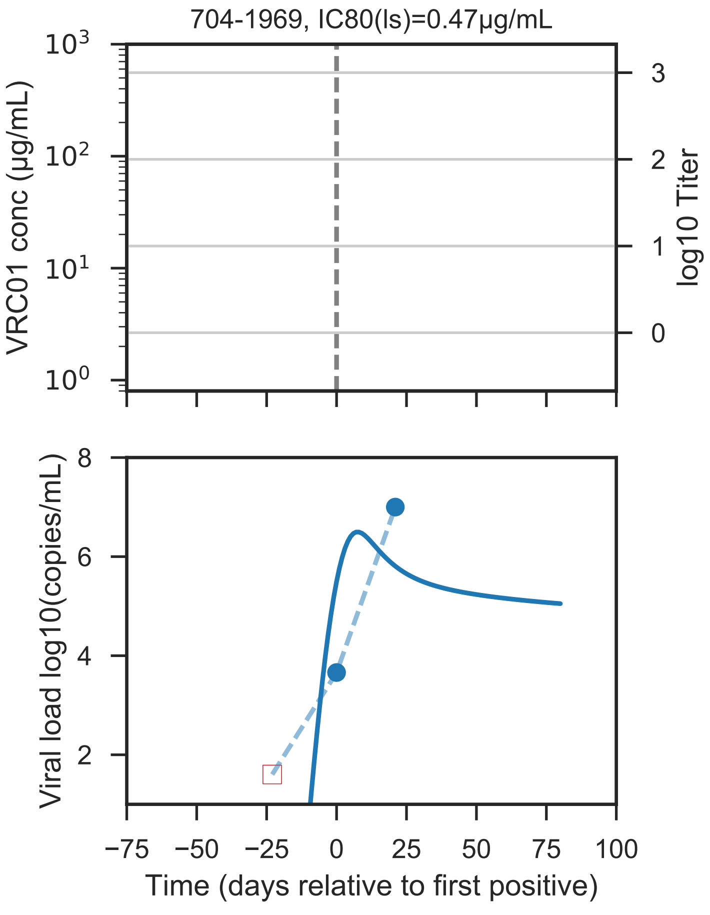

704-1969, IC80(ls)=0.47µg/mL
VRC01 conc (µg/mL)
10^3
10^2
10^1
10^0
log10 Titer
3
2
1
0
Viral load log10(copies/mL)
8
6
4
2
−75
−50
−25
0
25
50
75
100
Time (days relative to first positive)

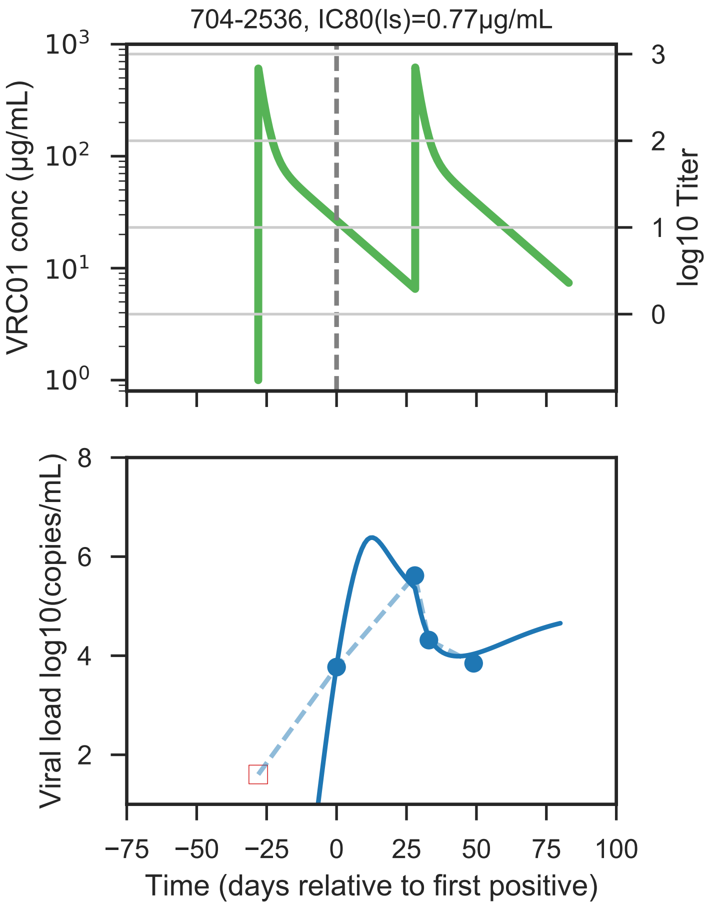

704-2536, IC80(ls)=0.77μg/mL
VRC01 conc (μg/mL)
log10 Titer
Viral load log10(copies/mL)
Time (days relative to first positive)
