# Supplementary figures and images for: High monoclonal neutralization titers reduced breakthrough HIV-1 viral loads in the Antibody Mediated Prevention trials
Source: Nat Commun. 2023 Dec 14;14:8299. doi: 10.1038/s41467-023-43384-y (PMC10721814; doi:10.1038/s41467-023-43384-y)

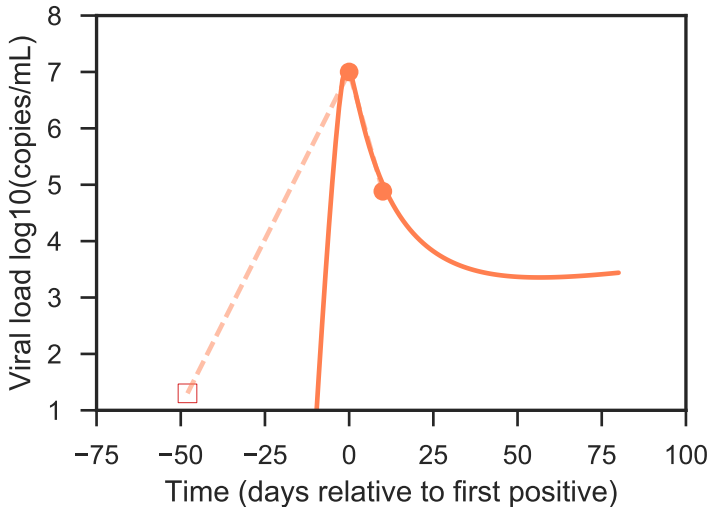

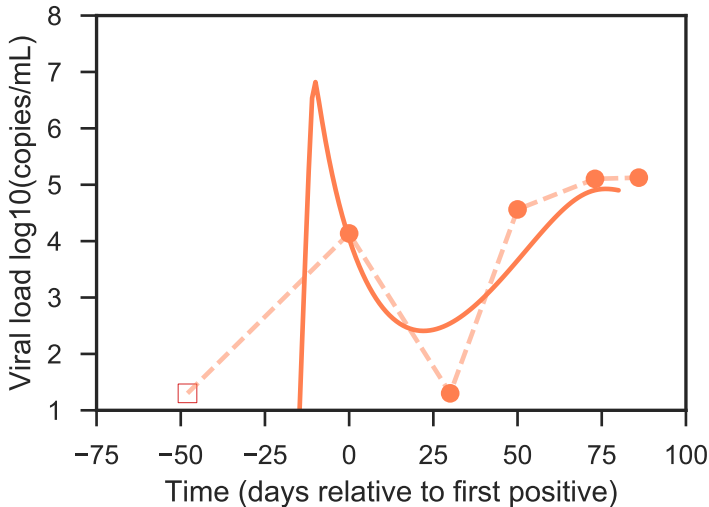

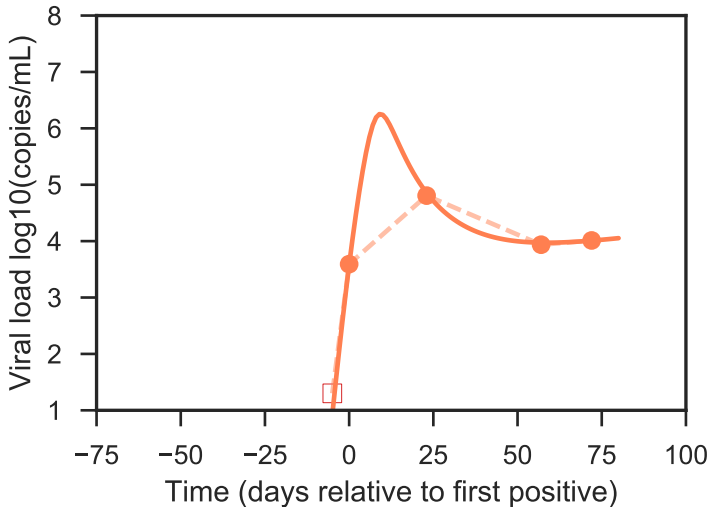

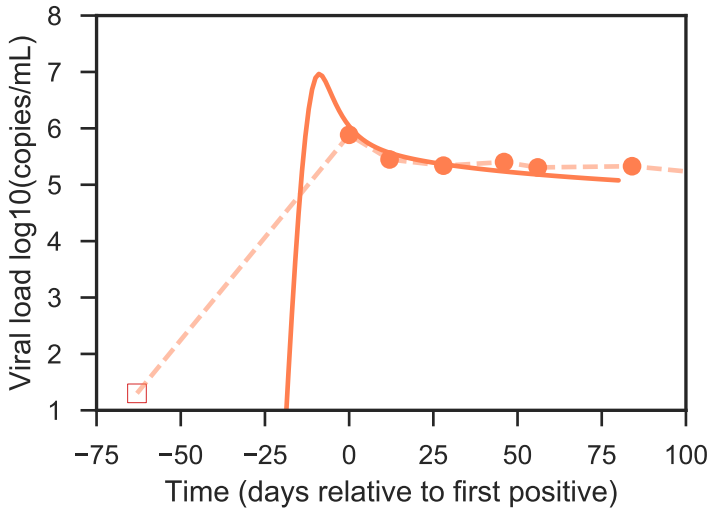

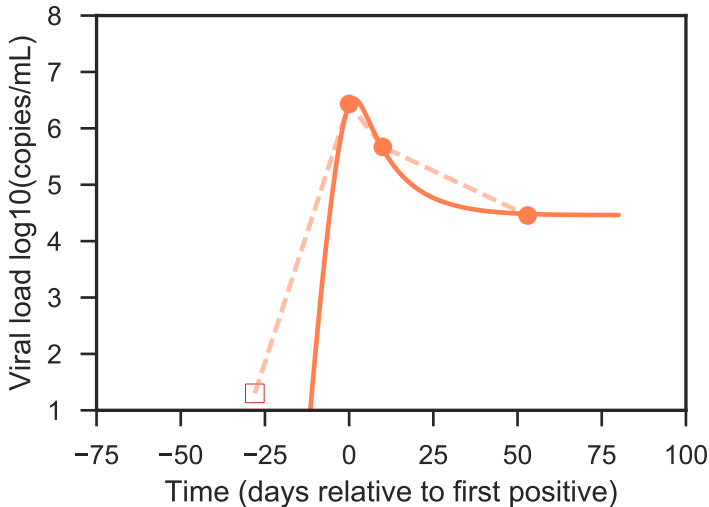

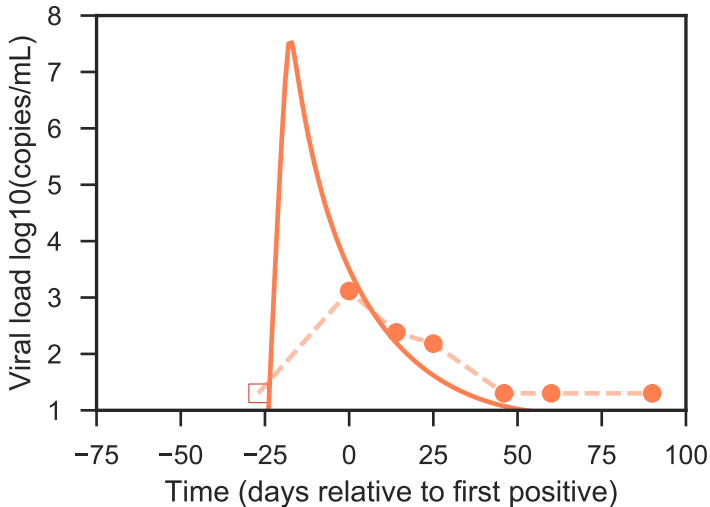

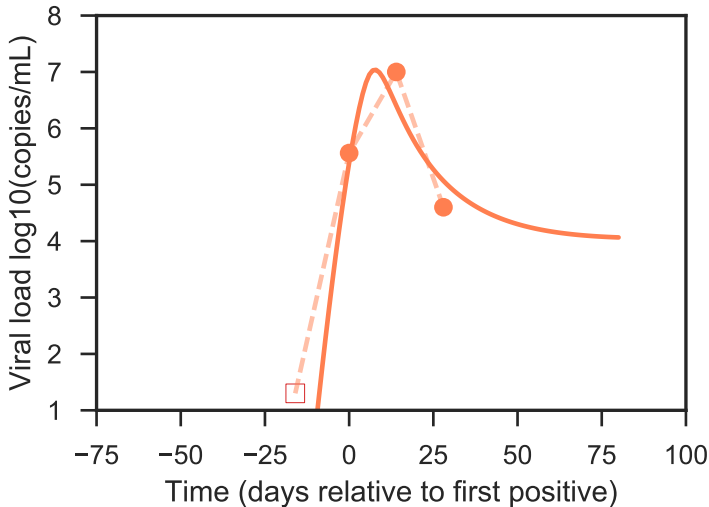

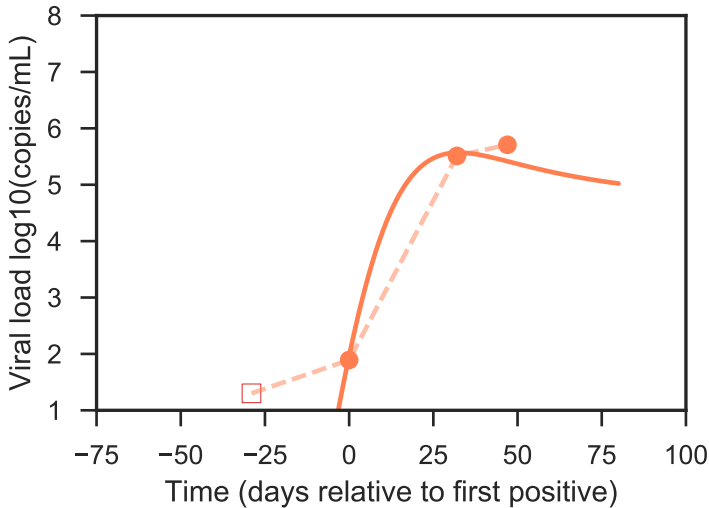

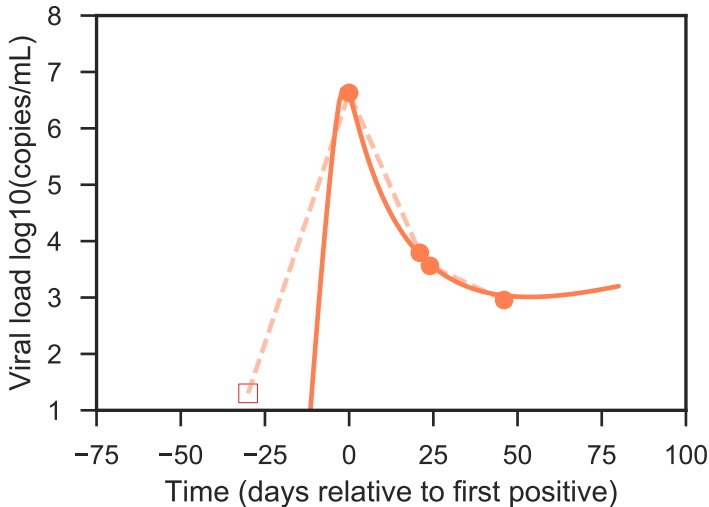

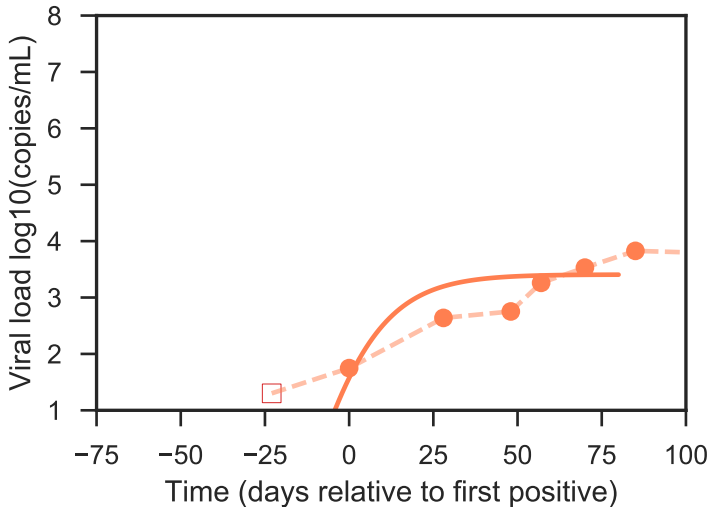

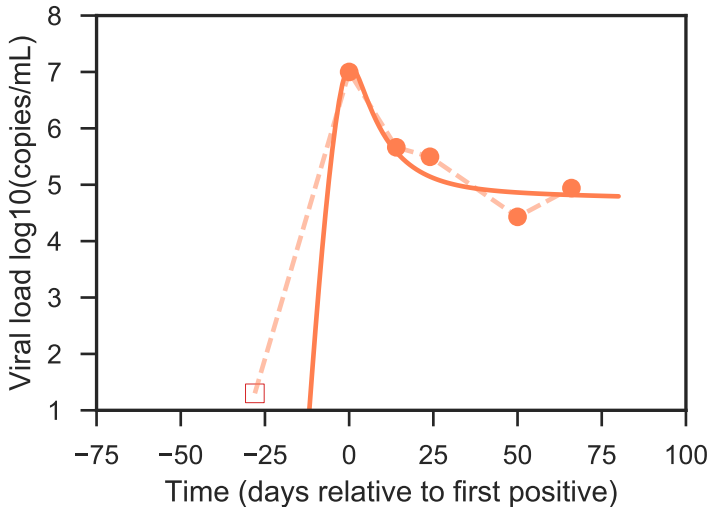

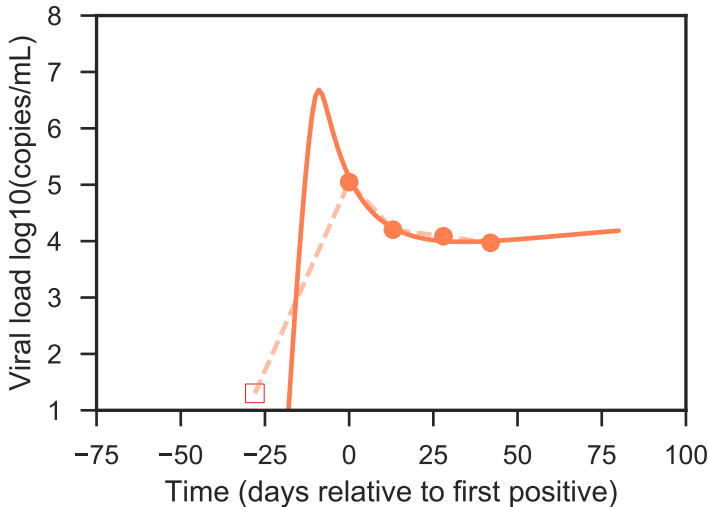

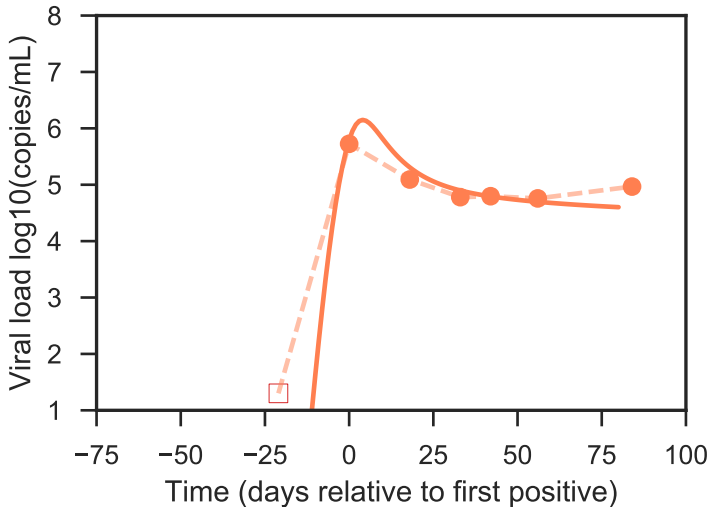

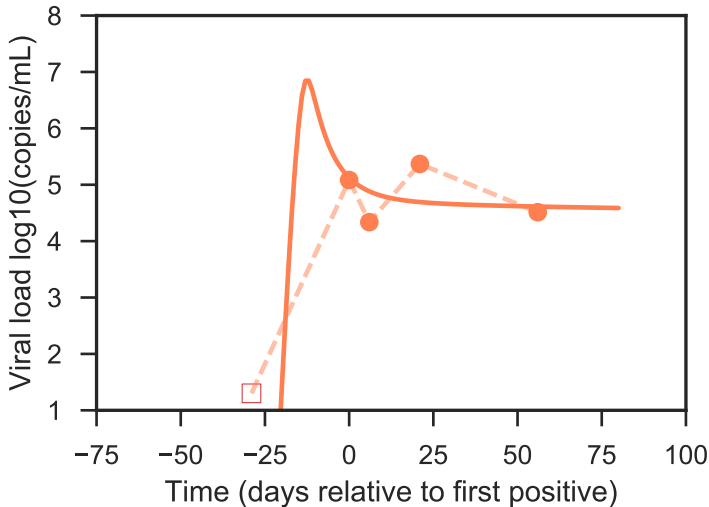

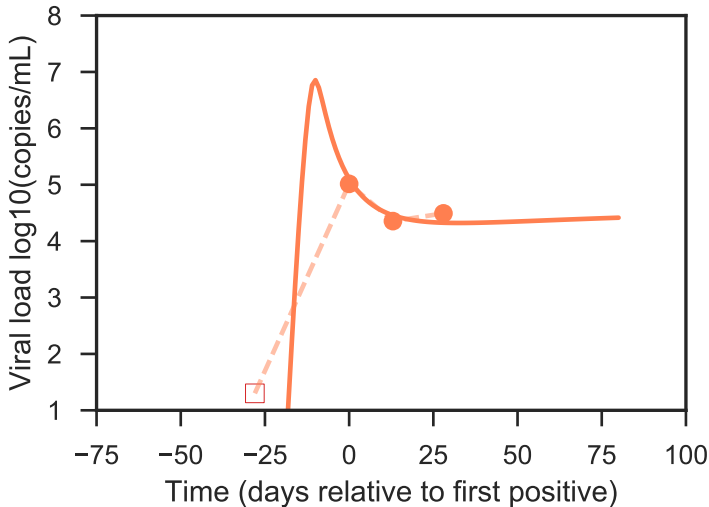

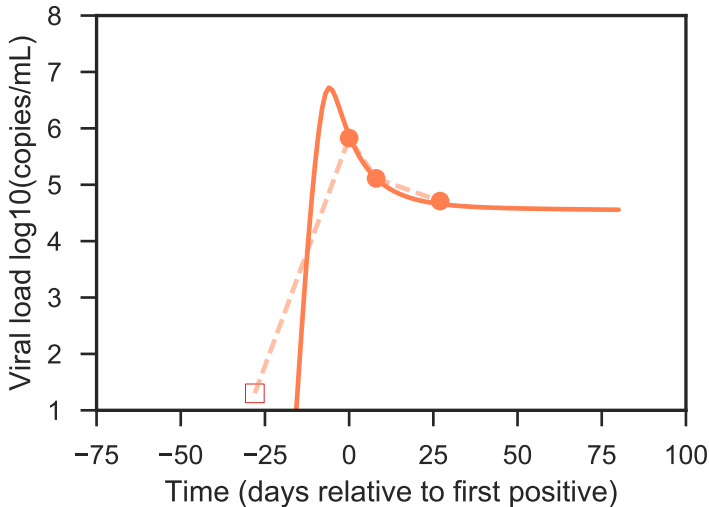

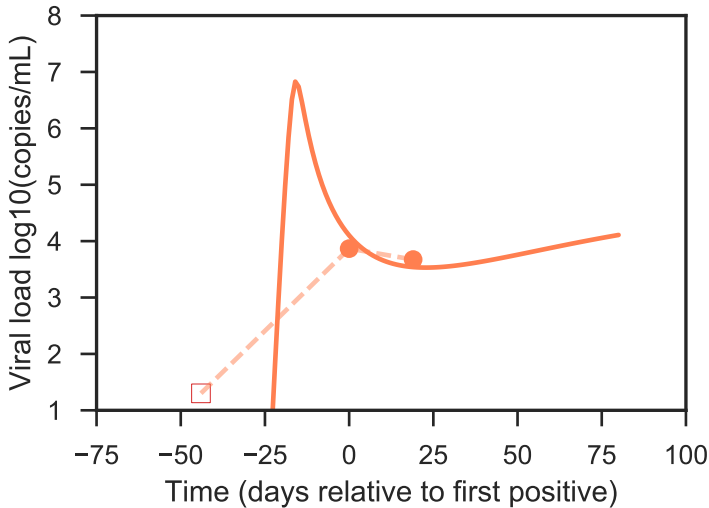

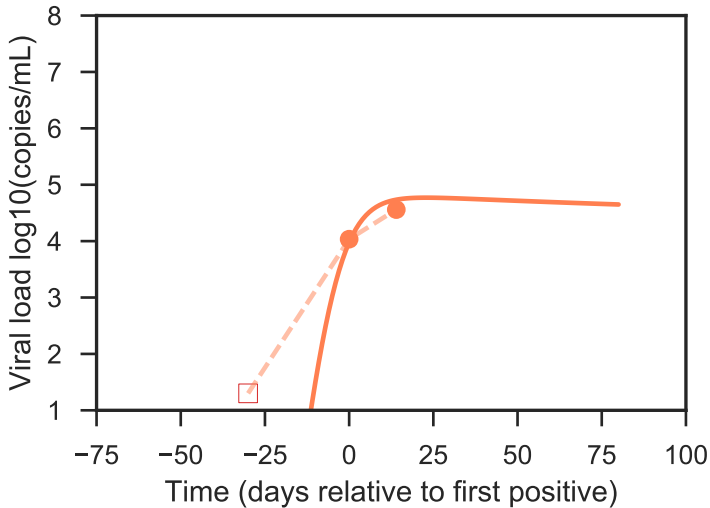

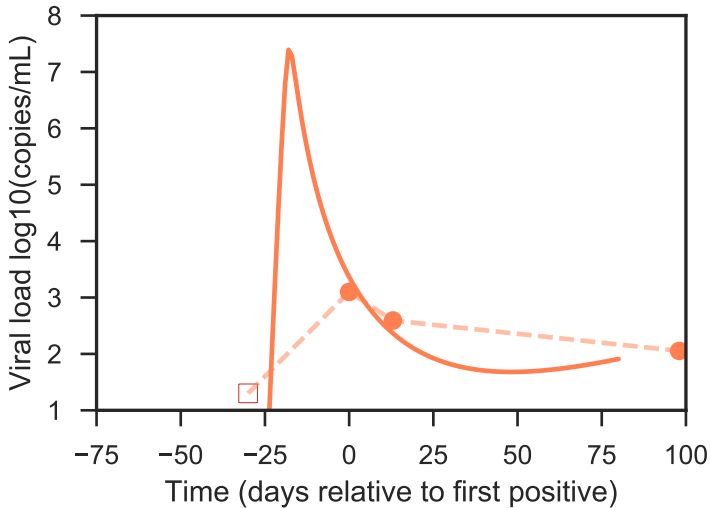

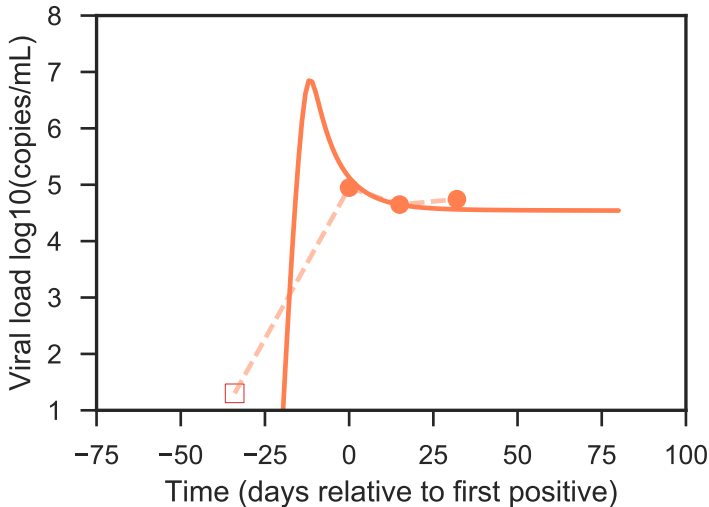

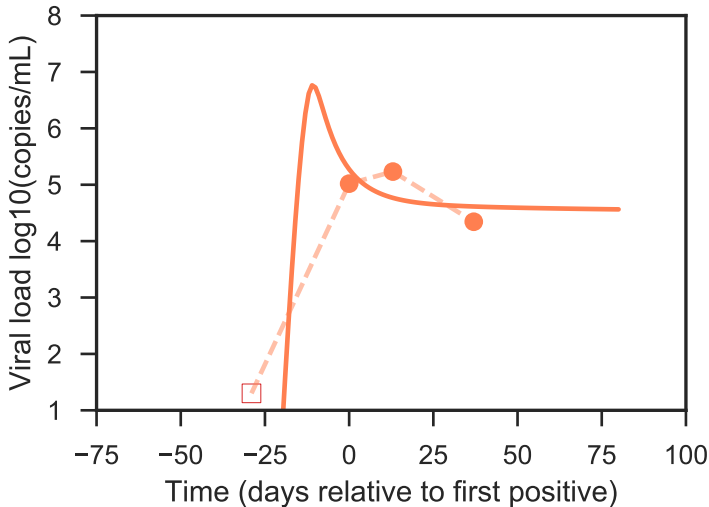

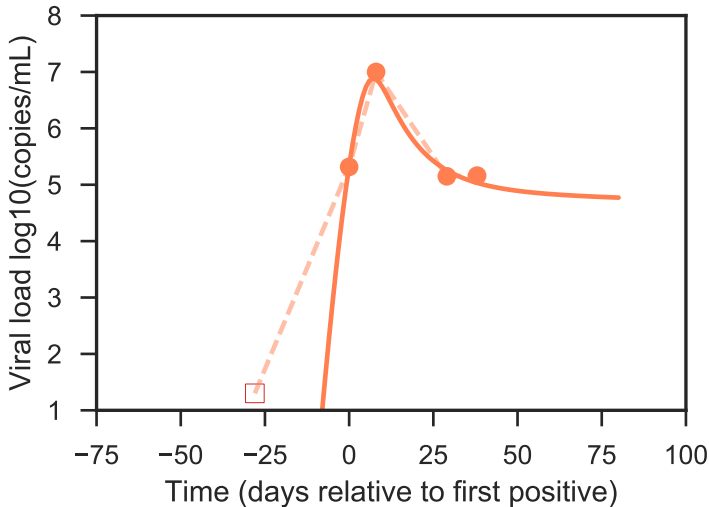

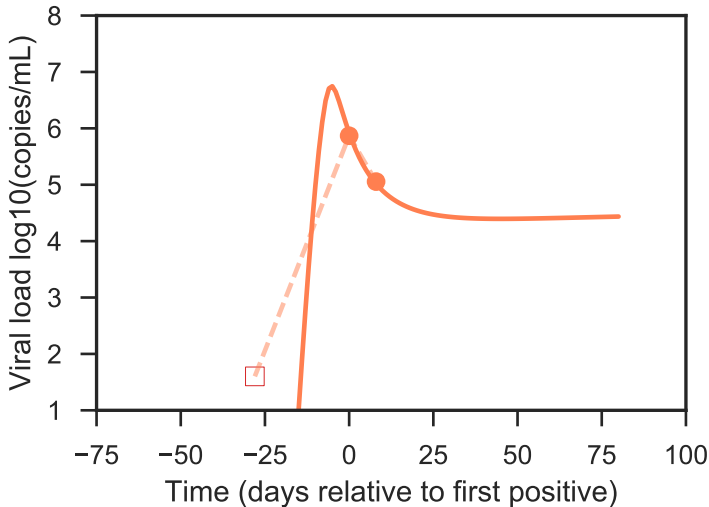

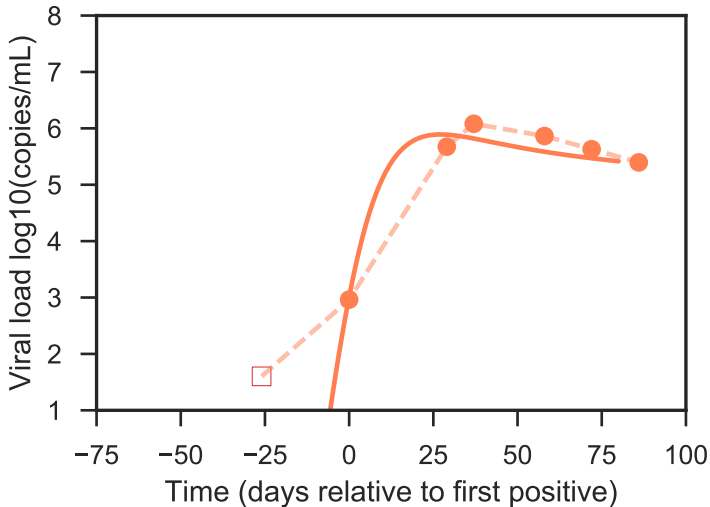

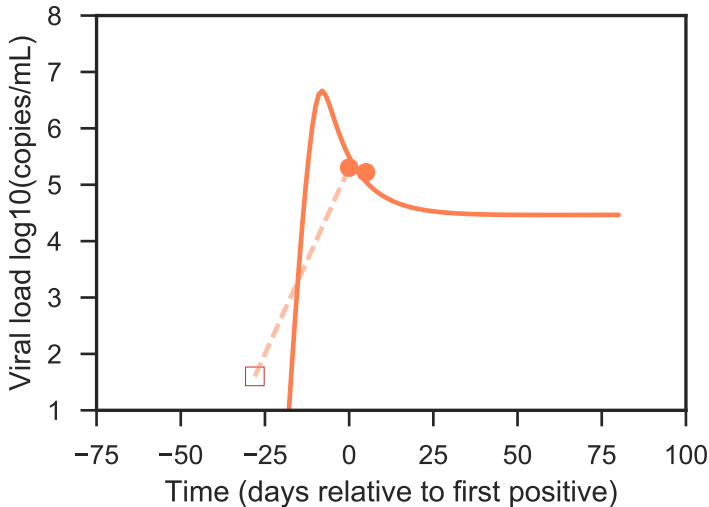

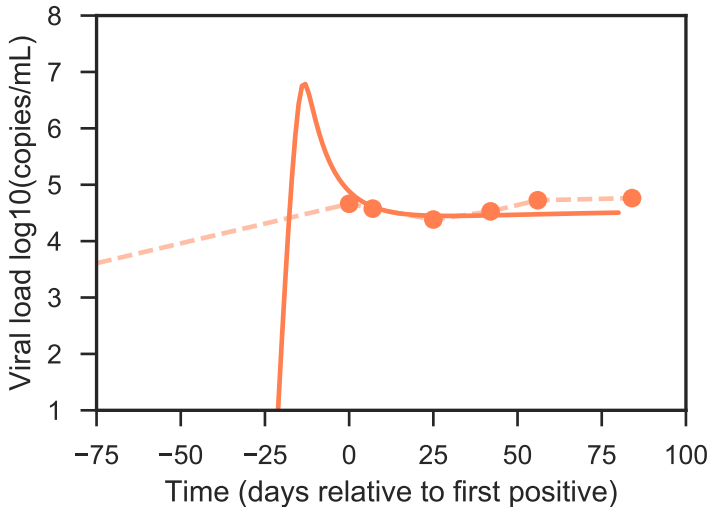

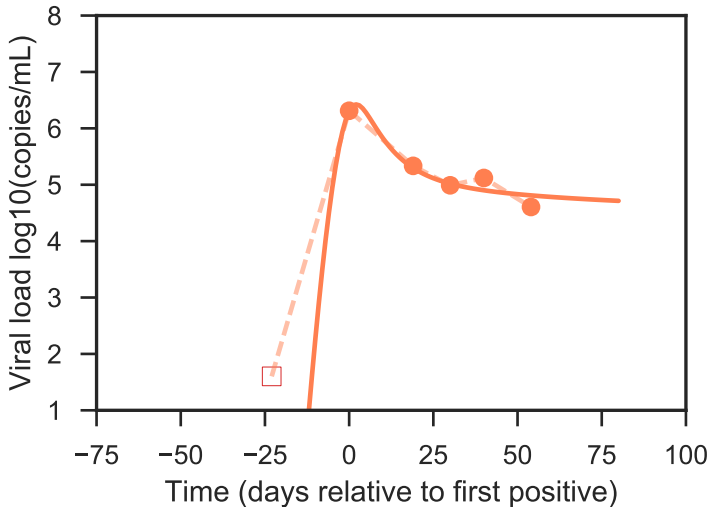

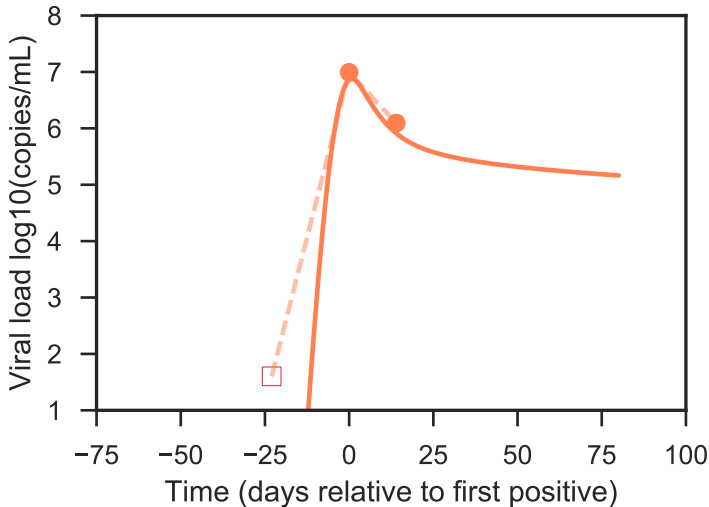

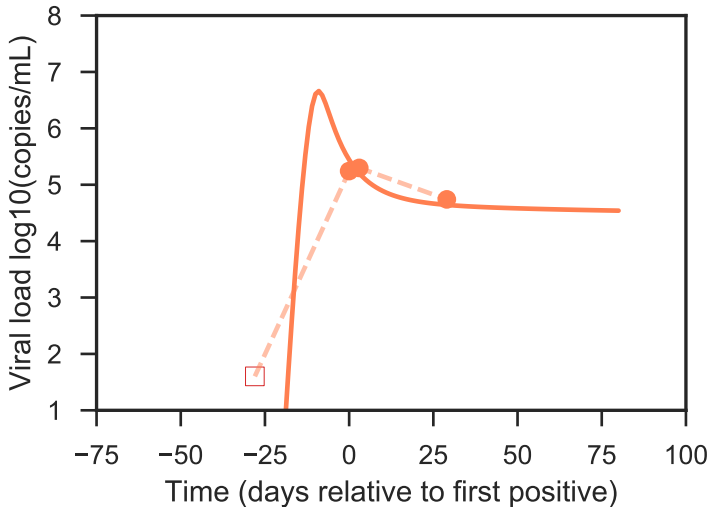

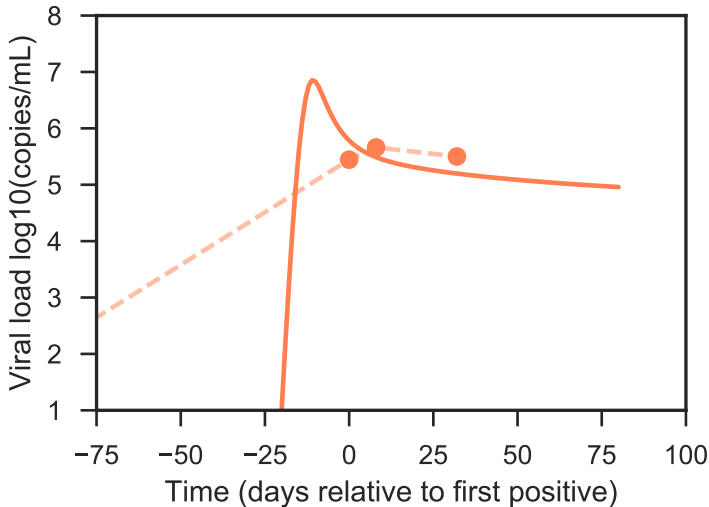

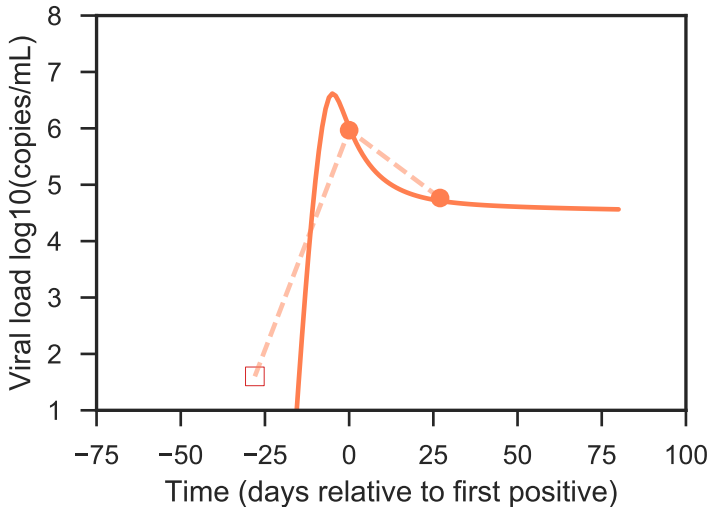

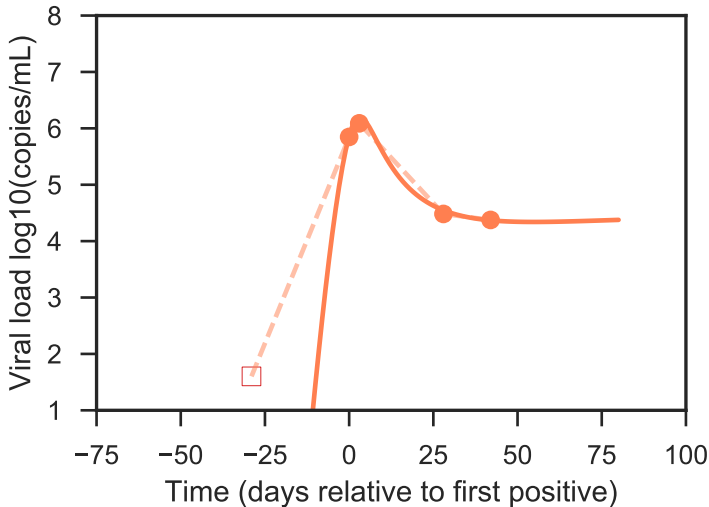

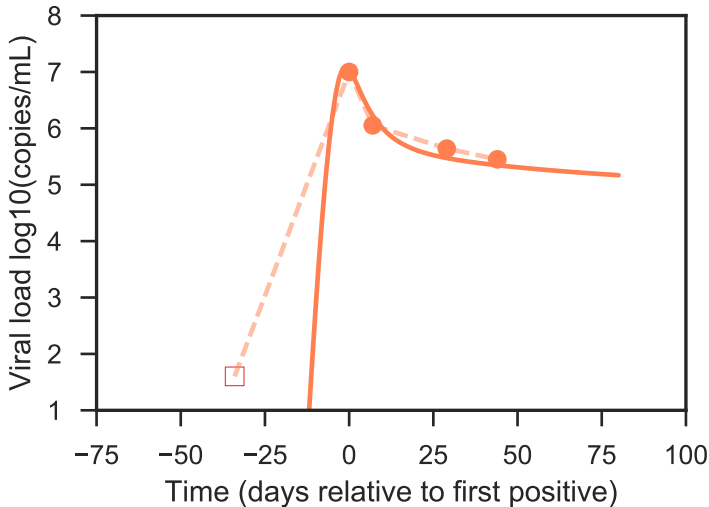

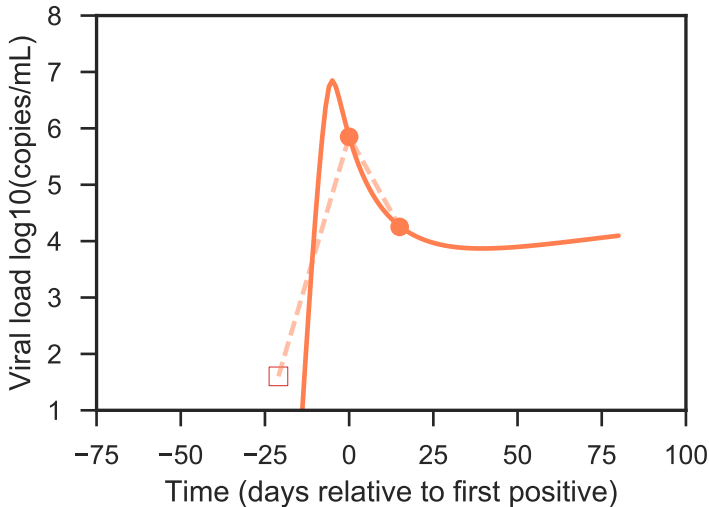

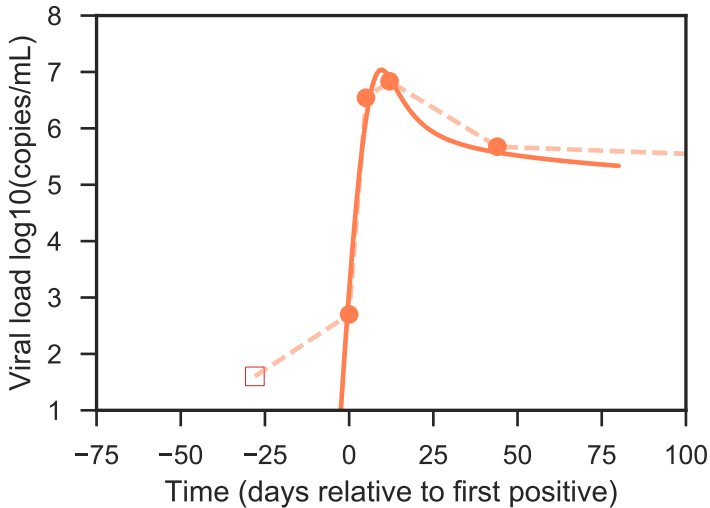

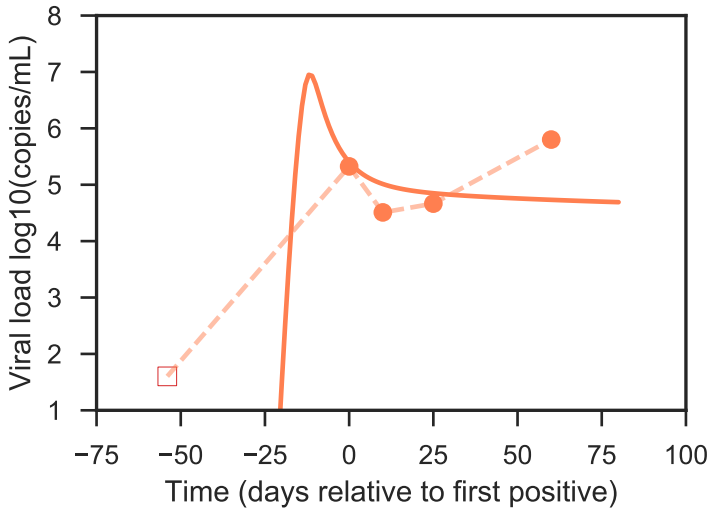

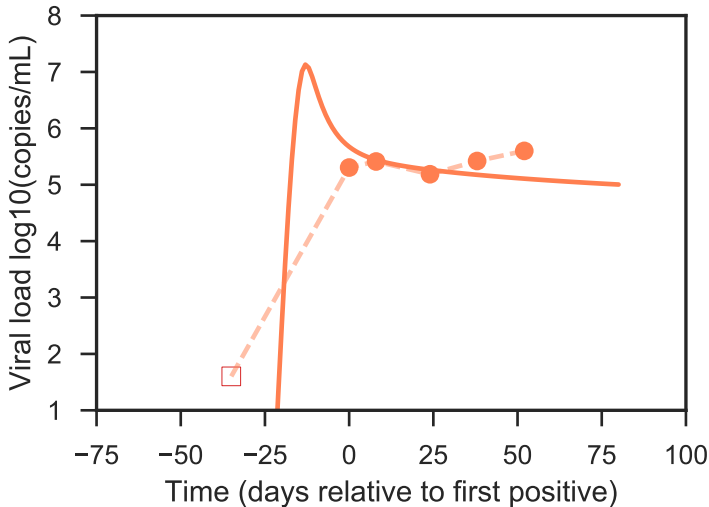

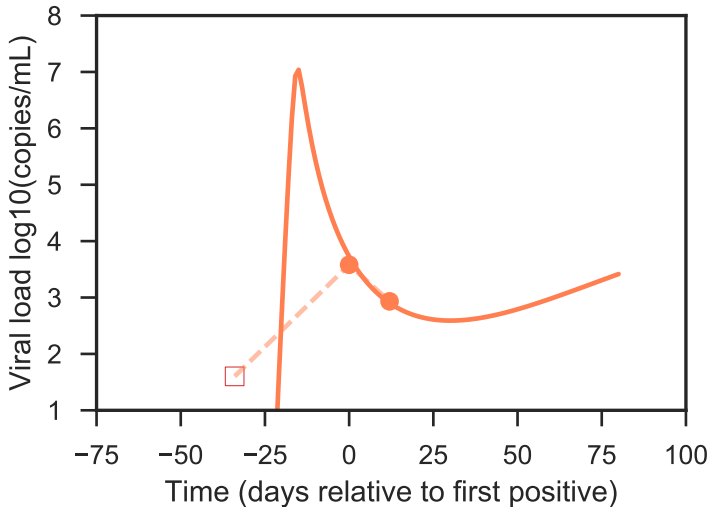

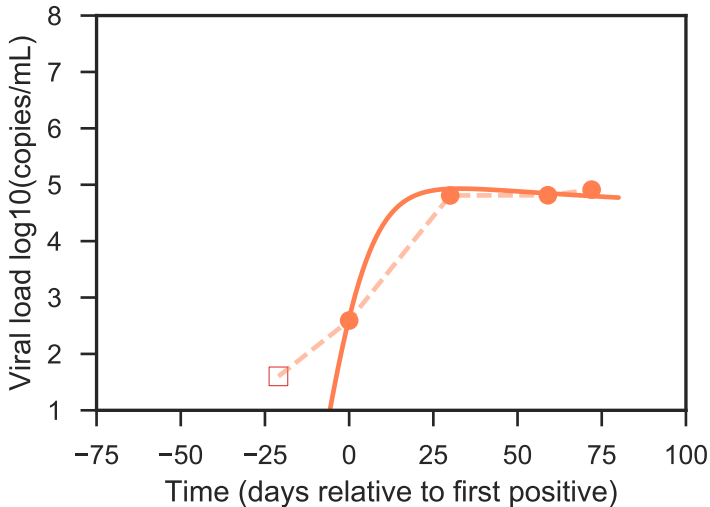

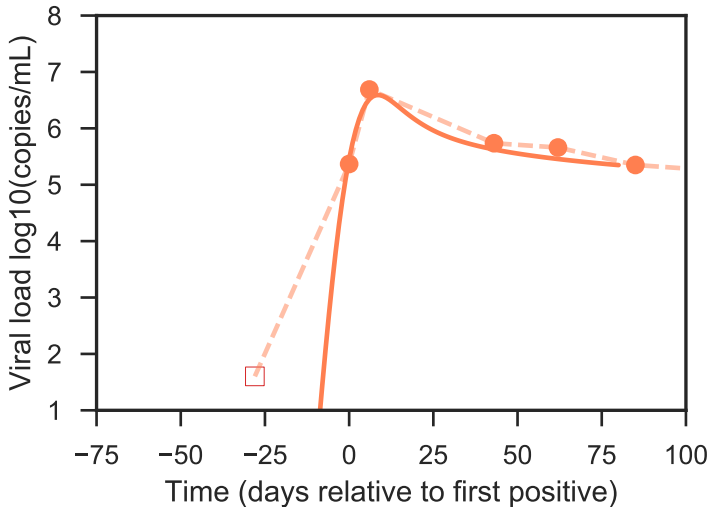

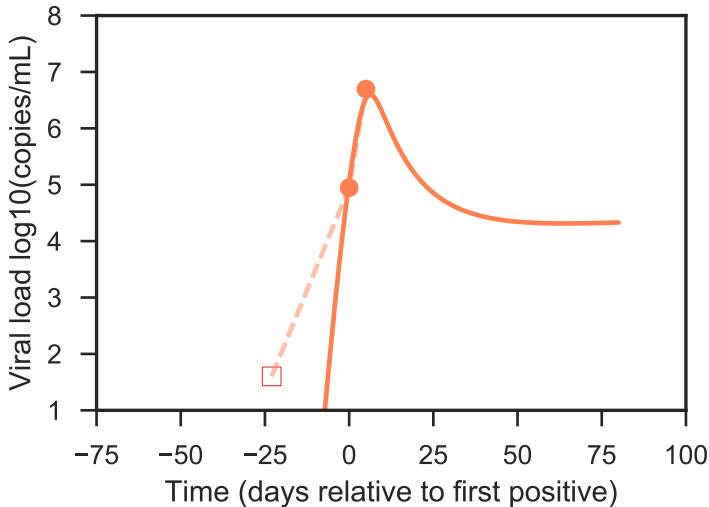

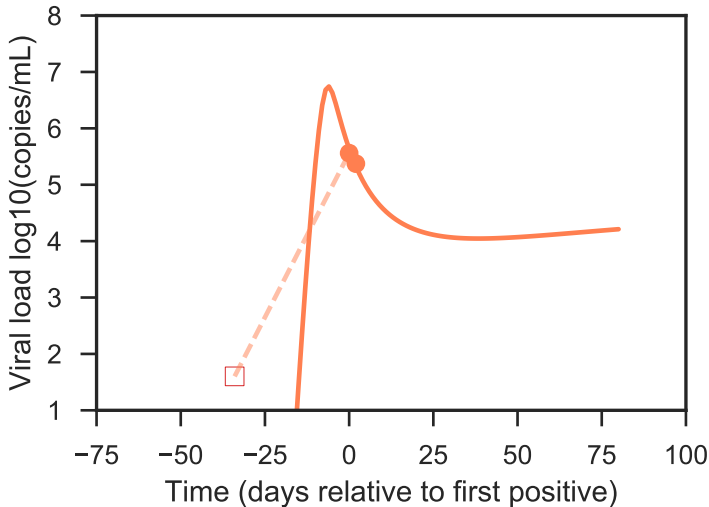

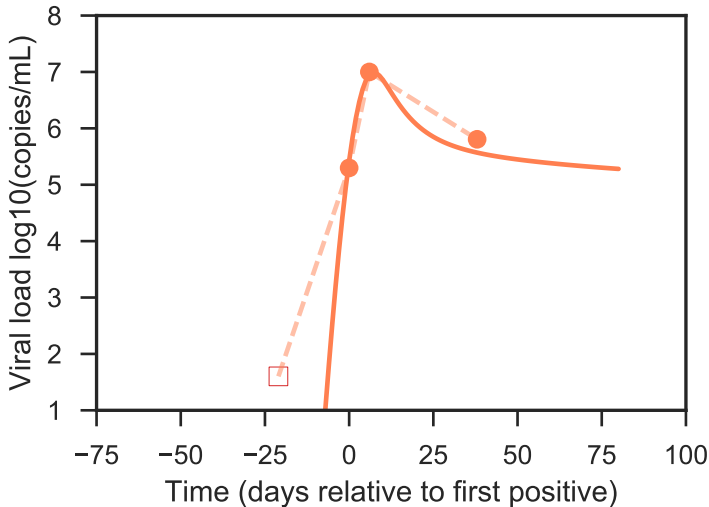

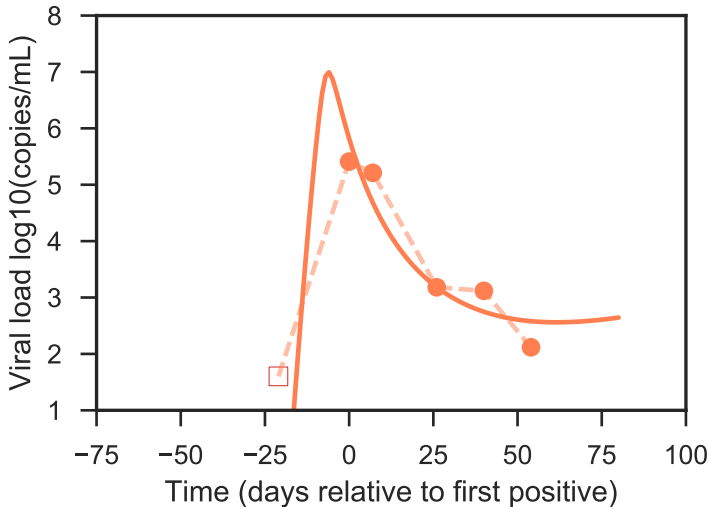

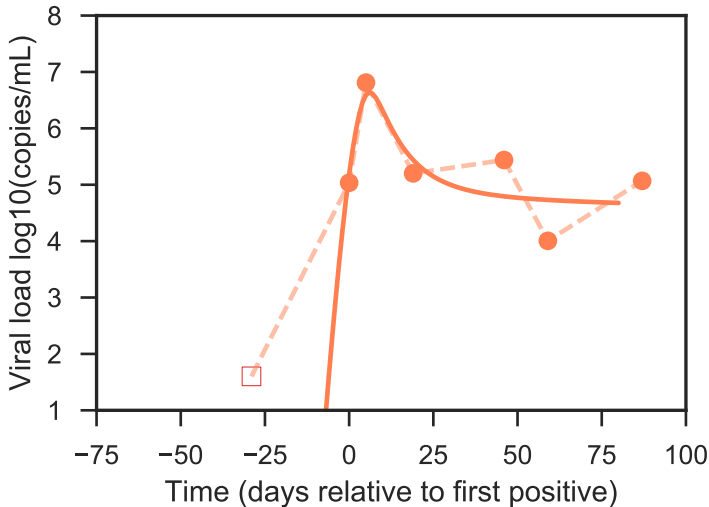

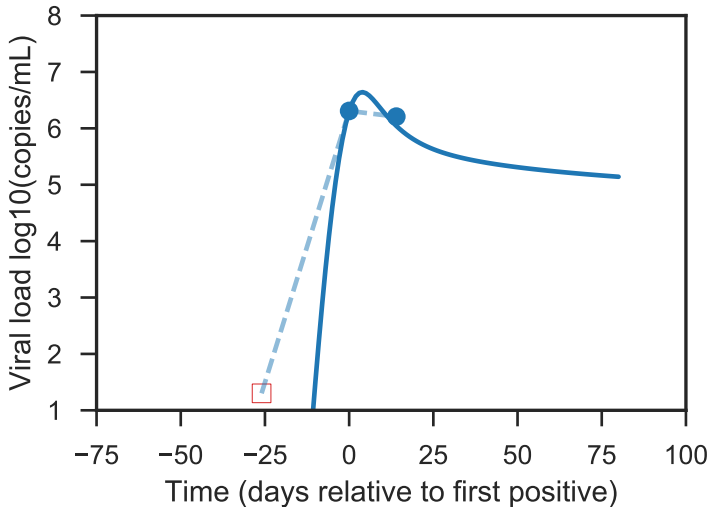

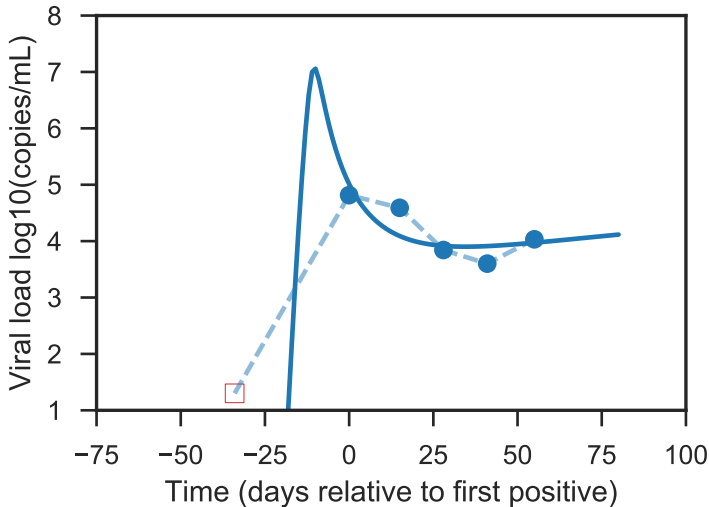

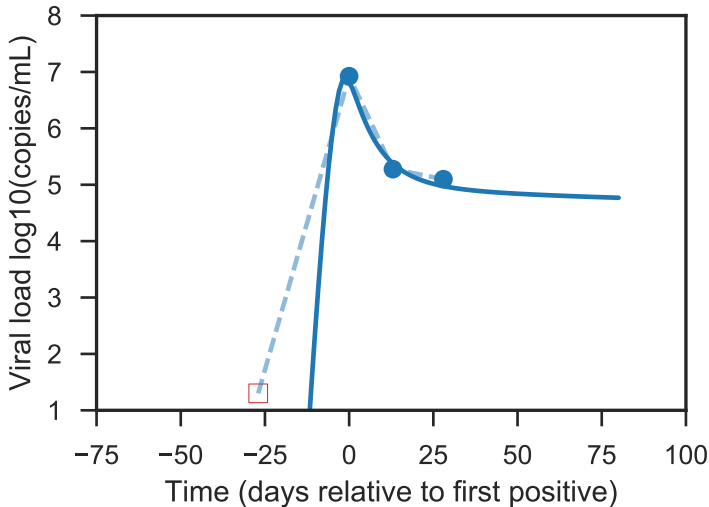

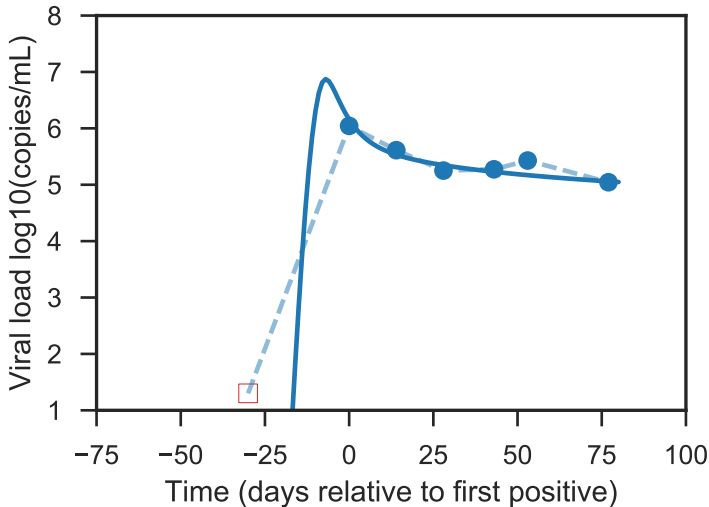

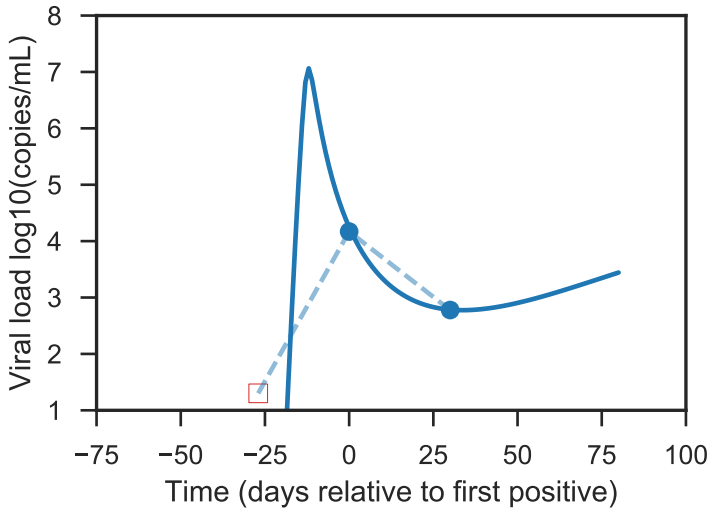

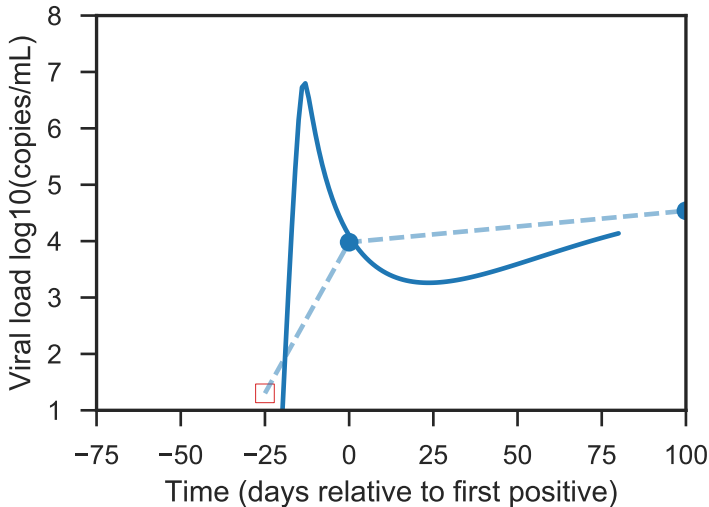

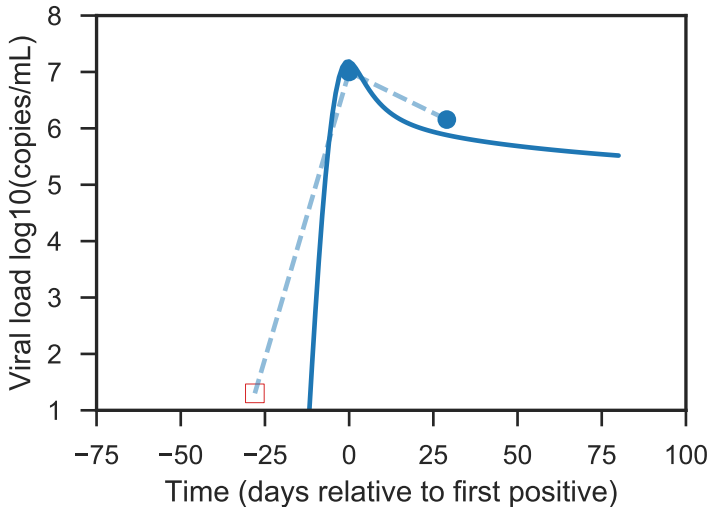

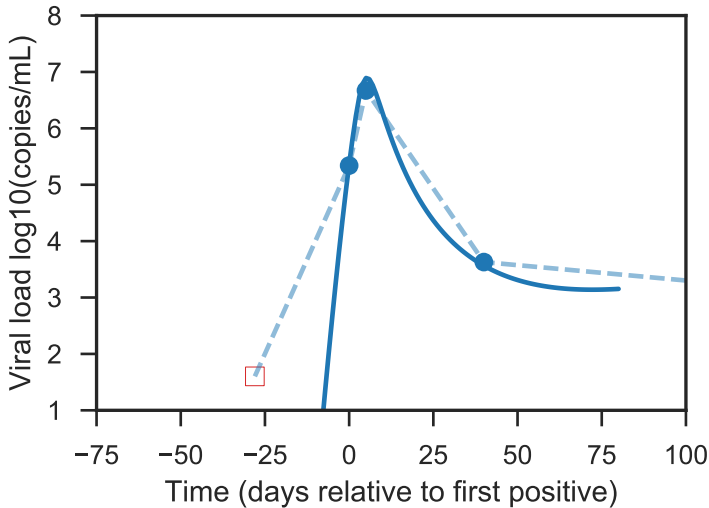

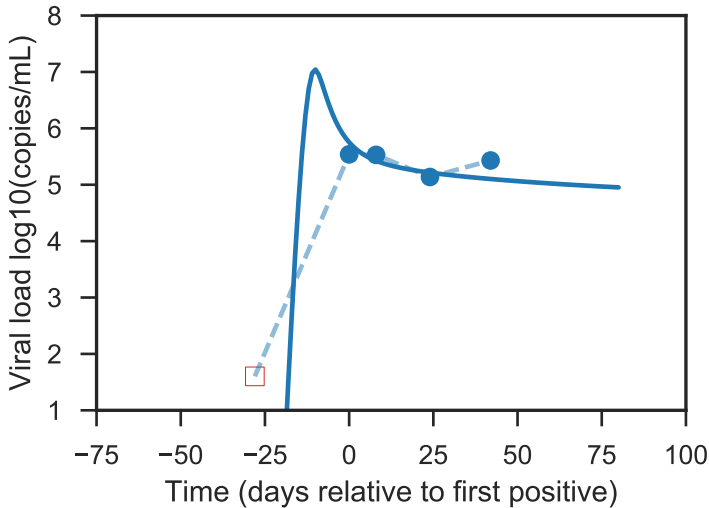

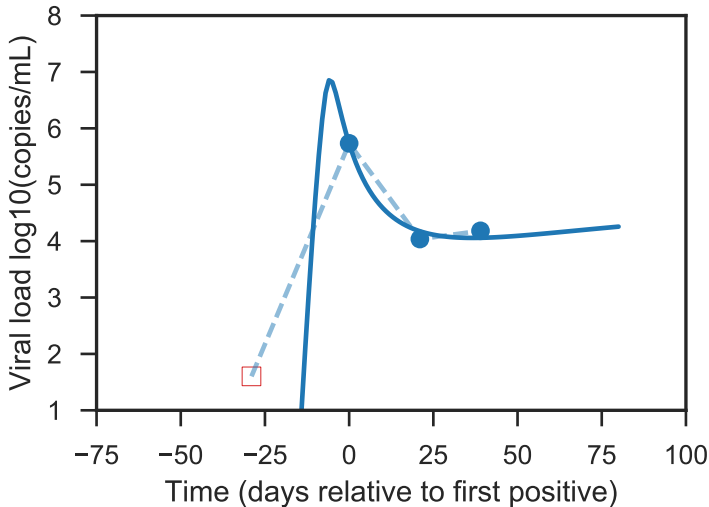

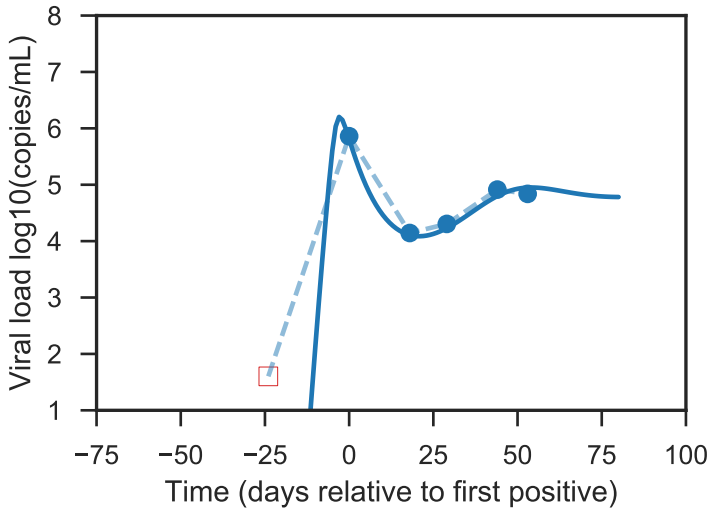

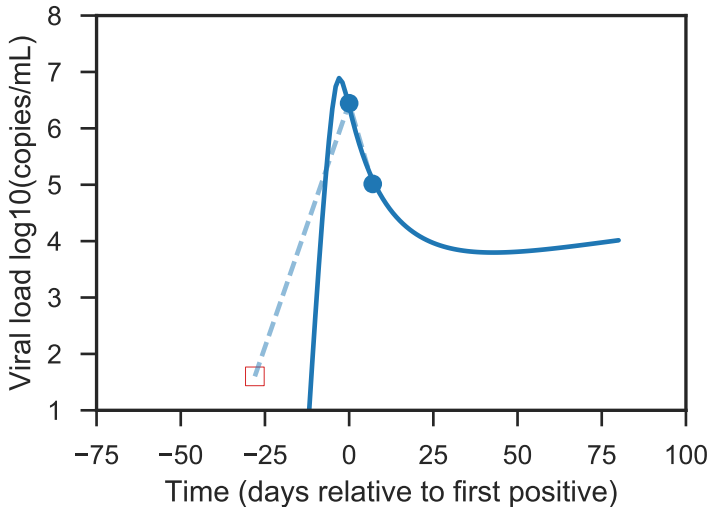

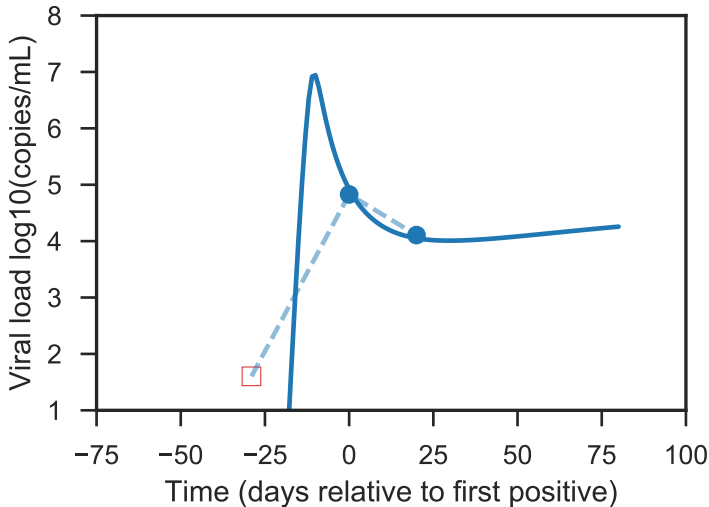

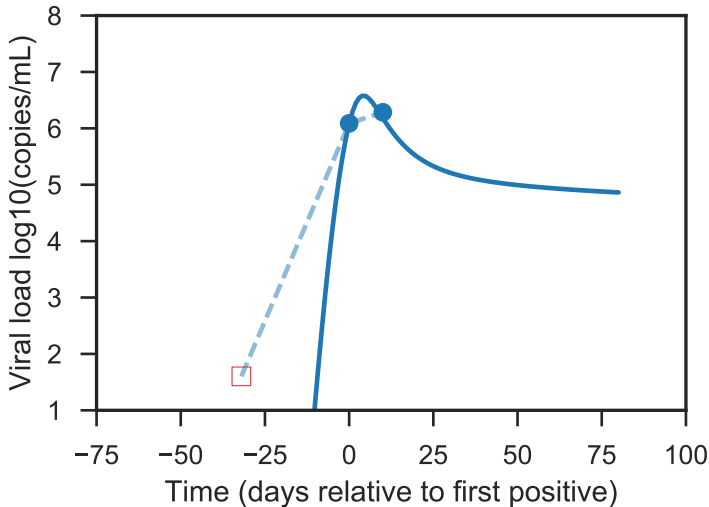

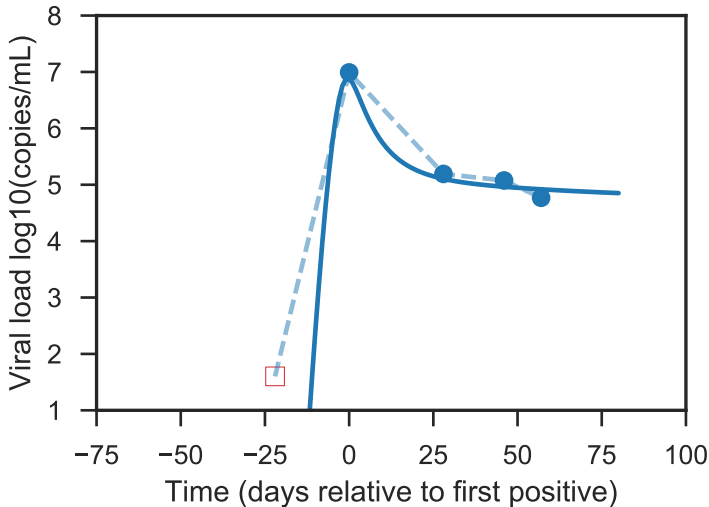

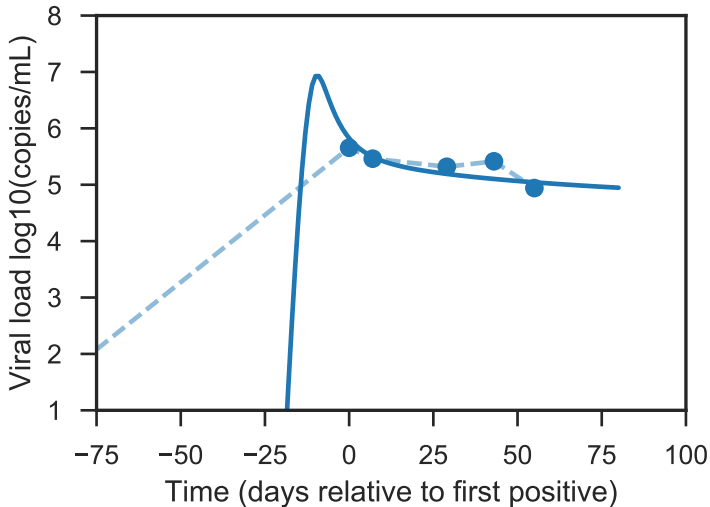

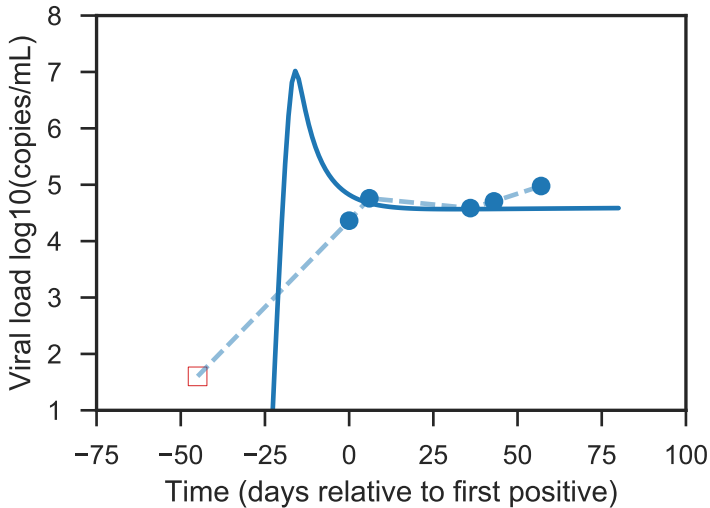

Supplement: Supplementary file 5 — Supplementary Data 2 [file 41467_2023_43384_MOESM5_ESM.pdf]
