# Supplementary material for: High monoclonal neutralization titers reduced breakthrough HIV-1 viral loads in the Antibody Mediated Prevention trials
Source: Nat Commun. 2023 Dec 14;14:8299. doi: 10.1038/s41467-023-43384-y (PMC10721814; doi:10.1038/s41467-023-43384-y)
Supplement: Supplementary file 7 — Reporting Summary [file 41467_2023_43384_MOESM7_ESM.pdf]

nature portfolio

Corresponding author(s): Daniel B. Reeves

Last updated by author(s): Oct 27, 2023

Reporting Summary

Nature Portfolio wishes to improve the reproducibility of the work that we publish. This form provides structure for consistency and transparency in reporting. For further information on Nature Portfolio policies, see our Editorial Policies and the Editorial Policy Checklist.

Statistics

For all statistical analyses, confirm that the following items are present in the figure legend, table legend, main text, or Methods section.

n/a	Confirmed	
☐	☒	The exact sample size (*n*) for each experimental group/condition, given as a discrete number and unit of measurement
☐	☒	A statement on whether measurements were taken from distinct samples or whether the same sample was measured repeatedly
☐	☒	The statistical test(s) used AND whether they are one- or two-sided<br>*Only common tests should be described solely by name; describe more complex techniques in the Methods section.*
☐	☒	A description of all covariates tested
☐	☒	A description of any assumptions or corrections, such as tests of normality and adjustment for multiple comparisons
☐	☒	A full description of the statistical parameters including central tendency (e.g. means) or other basic estimates (e.g. regression coefficient) AND variation (e.g. standard deviation) or associated estimates of uncertainty (e.g. confidence intervals)
☐	☒	For null hypothesis testing, the test statistic (e.g. *F*, *t*, *r*) with confidence intervals, effect sizes, degrees of freedom and *P* value noted<br>*Give P values as exact values whenever suitable.*
☒	☐	For Bayesian analysis, information on the choice of priors and Markov chain Monte Carlo settings
☒	☐	For hierarchical and complex designs, identification of the appropriate level for tests and full reporting of outcomes
☐	☒	Estimates of effect sizes (e.g. Cohen's *d*, Pearson's *r*), indicating how they were calculated

Our web collection on statistics for biologists contains articles on many of the points above.

Software and code

Policy information about availability of computer code

Data collection	Laboratory data were collected at central research labs. For Duke, from the start of the study a luminometer called the Victor X Light was used for data collection using The PerkinElmer 2030 software (Instrument Program version = 4.00.05) which is a 32-bit application running under Windows 7. Starting November 11th 2020, after IQ/OQ, a new luminometer was used called Glomax Navigator System using Glomax Navigator software (software version 3.2.3, firmware version 4.92.0). For NICD, for the duration of the study we used the PerkinElmer Victor X Luminometer for data collection using the PerkinElmer 2030 software.
Data analysis	All code for data analysis and figure generation is freely available from the authors (https://github.com/FredHutch/AMPVLAnalysis). Python (v 3.10), R (v 4.2), ggplot2 (v 3.4.4), and the Seaborn (v 0.7.1) package were used to streamline the modeling pipeline and make figures.

For manuscripts utilizing custom algorithms or software that are central to the research but not yet described in published literature, software must be made available to editors and reviewers. We strongly encourage code deposition in a community repository (e.g. GitHub). See the Nature Portfolio guidelines for submitting code & software for further information.

Data

Policy information about availability of data

All manuscripts must include a data availability statement. This statement should provide the following information, where applicable:

- Accession codes, unique identifiers, or web links for publicly available datasets
- A description of any restrictions on data availability
- For clinical datasets or third party data, please ensure that the statement adheres to our policy

The data generated in this study are provided in Supplementary Data files 1-3, as well as the public-facing HVTN website (https://atlas.scharp.org/cpas/project/HVTN%20Public%20Data/begin.view?). All individual participant data have been deidentified. The HIV-1 Env clones used in the TZM-bl target cell neutralization assay are available at the GenBank database (https://www.ncbi.nlm.nih.gov/genbank/) under the following accession codes: HVTN 704/HPTN085 sequences, ON980814–ON980967; HVTN 703/HPTN081 sequences, ON890939–ON891092.

Research involving human participants, their data, or biological material

Policy information about studies with human participants or human data. See also policy information about sex, gender (identity/presentation), and sexual orientation and race, ethnicity and racism.

Reporting on sex and gender

In AMP, sex assigned at birth and gender identity were self-reported. As the majority of HIV-1 transmissions occur via the vaginal or rectal mucosa, and bnAb distribution at these mucosa may vary at these two sites (with potential implications on prevention efficacy), sex/gender identity was considered in trial design.
In HVTN 704/HPTN 085, participants who were assigned male sex at birth or who were transgender (transfemale or transmale) and met other eligibility criteria were eligible. ~ 10% of ppts were transgender and the remainder were cisgender male. The exact numbers are provided in Methods and were originally reported in Edupuganti et al. 2021 (JAIDS).
In HVTN 703/HPTN 081, participants who were assigned female sex at birth and met other eligibility criteria were eligible. The exact numbers are provided in Methods and were originally reported in Mgodi et al. 2021 (JAIDS).
The trial eligibility criteria-- i.e., the majority or entirely cisgender female or cisgender male population in each trial-- preclude a meaningful analysis by sex and/or gender distinct from the analysis by trial, which was done and is included here.

Reporting on race, ethnicity, or other socially relevant groupings

As provided in the protocols of the HVTN 703/HPTN 081 and HVTN 704/HPTN 085 trials (https://www.nejm.org/doi/suppl/10.1056/NEJMoa2031738/suppl_file/nejmoa2031738_protocol.pdf), volunteer demographics were obtained in compliance with the NIH Policy on
Reporting Race and Ethnicity Data: Subjects in Clinical Research, Aug. 8, 2001 (https://grants.nih.gov/grants/guide/notice-files/NOT-OD-01-053.html). Thus, the categories were as follows:

Ethnic Categories:
Hispanic or Latino: A person of Cuban, Mexican, Puerto Rican, South or Central American, or other Spanish culture or origin, regardless of race. The term "Spanish origin" can also be used in addition to "Hispanic or Latino."
Not Hispanic or Latino

Racial Categories:
American Indian or Alaska Native: A person having origins in any of the original peoples of North, Central, or South America, and who maintains tribal affiliations or community attachment.
Asian: A person having origins in any of the original peoples of the Far East, Southeast Asia, or the Indian subcontinent including, for example, Cambodia, China, India, Japan, Korea, Malaysia, Pakistan, the Philippine Islands, Thailand, and Vietnam. (Note: Individuals from the Philippine Islands have been recorded as Pacific Islanders in previous data collection strategies.)
Black or African American: A person having origins in any of the black racial groups of Africa. Terms such as "Haitian" or "Negro" can be used in addition to "Black or African American."
Native Hawaiian or Other Pacific Islander: A person having origins in any of the original peoples of Hawaii, Guam, Samoa, or other Pacific Islands.
White: A person having origins in any of the original peoples of Europe, the Middle East, or North Africa.

Respondent self-report or self-identification was used to collect an individual's data on ethnicity and race.

Of the 2701 participants enrolled in HVTN 704/HPTN 085, 1545 were Hispanic or Latinx and 1156 Not Hispanic or Latinx; 851 were White, 409 were Black or African American, 69 were Asian, 8 were Native Hawaiian/other Pacific Islander, 17 were American Indian/Alaska Native, and 1259 were Other (Edupuganti et al. 2021 JAIDS).
Of the 1924 participants enrolled in HVTN 703/HPTN 081, 1902 were Black, 9 were Asian, 12 were Other, and 1 was Multiracial (Mgodi et al. 2021 JAIDS).

Population characteristics

HVTN 704/HPTN 085 enrolled 2,701 participants who had been assigned male sex at birth or who self-reported as being transgender and who had sex with cisgender men or transgender persons. Participants ranged from 18 to 50 years of age and were enrolled at sites in the US, Brazil, Peru, and Switzerland between April 2016 and October 2018. HVTN 703/HPTN 081 enrolled 1,924 participants who had been assigned female sex at birth, 18 to 50 years of age, in 7 countries in sub-Saharan Africa between May 2016 and September 2018. Demographic characteristics of the participants in the AMP trials are provided in Table 1 of Corey and Gilbert et al. NEJM (2021), as well as in Edupuganti et al. 2021 JAIDS and in Mgodi et al. 2021 JAIDS.

Recruitment

Participant recruitment for the AMP trials is detailed in Edupuganti et al. 2021 JAIDS May 1;87(1):671-679. "Community

Recruitment	engagement began 6 months before study opening. Community stakeholder meetings with diverse audiences were held regionally and nationally. Recruitment and retention were considered major challenges for the AMP studies, given the 10 IV infusions and 2-year (or 104 week) duration of study participation. Print materials and animated videos were developed in multiple languages. Internet-based recruitment (through social media, Craigslist, dating Web sites, and others), face-to-face outreach by recruiters, and referrals from other participants were some of the key strategies that were used for recruitment. Regional protocol-specific Web sites (www.ampstudy.org) were developed to enhance education of potential study participants and link them to local CRSs." We are not aware of any potential self-selection bias or other biases that may impact results.
Ethics oversight	All work described here complied with all relevant ethical regulations. This work was approved by the Duke University Health System Institutional Review Board (Duke University) through protocol ID Pro00093087. For the NICD the work was approved by the University of the Witwatersrand Human Research Ethics Committee through protocol M201105. All participants provided written informed consent.

Note that full information on the approval of the study protocol must also be provided in the manuscript.

Field-specific reporting

Please select the one below that is the best fit for your research. If you are not sure, read the appropriate sections before making your selection.

☒ Life sciences ☐ Behavioural & social sciences ☐ Ecological, evolutionary & environmental sciences

For a reference copy of the document with all sections, see nature.com/documents/nr-reporting-summary-flat.pdf

Life sciences study design

All studies must disclose on these points even when the disclosure is negative.

Sample size	Sample sizes of the two AMP trials were predetermined using a 1-sided 0.025-level Wald test for comparing log-transformed cumulative incidences of HIV-1 inection between the pooled VRC01 groups versus the control group as described in the protocol and in Gilbert et al. (SCID 2017). Power calculations for the case-control study were described in SCID 2017 and further studied in Gilbert et al. (Stat Med, 2019).
Data exclusions	There are pre-established exclusion criteria applied to all data in the form of an assay SOP QC checklist, any assays that did not pass the checklist criteria were repeated.
Replication	This study utilized two sets of neutralizing antibody assay results, one set was generated with a clinical lot of VRC01 and a second set was generated with autologous serum samples. Assays with the clinical lot of VRC01 were performed three times, where each time the samples were tested in duplicate wells. The three titer values were averaged. Assays with autologous serum samples were performed once using duplicate wells. VRC01 drug product was used as a positive control in each assay run. The assay has been formally validated for accuracy, sensitivity, specificity, precision, linearity, range and robustness. For in vitro neutralization measurements (IC50 or IC80), duplicate values for wells that scored at least 40% neutralization must have agreed within 30% to have passed quality control.
Randomization	In the AMP trials, participants were randomly assigned to treatment arm as described in Corey and Gilbert et al. 2021 NEJM. As described in that reference, the randomization sequence was obtained by computer-generated random numbers and provided to each CRS through a Web-based randomization system. The randomization was done in blocks to ensure balance across arms. At each institution, the pharmacist with primary responsibility for dispensing study products was charged with maintaining security of the treatment assignments (except in emergency situations as specified in the SSP).
Blinding	Laboratory staff conducting the TZM-bl target cell assays were blinded to group allocation during data collection and analysis.

Reporting for specific materials, systems and methods

We require information from authors about some types of materials, experimental systems and methods used in many studies. Here, indicate whether each material, system or method listed is relevant to your study. If you are not sure if a list item applies to your research, read the appropriate section before selecting a response.

Materials & experimental systems

n/a	Involved in the study	
☐	☒	Antibodies
☐	☒	Eukaryotic cell lines
☒	☐	Palaeontology and archaeology
☒	☐	Animals and other organisms
☐	☒	Clinical data
☐	☒	Dual use research of concern
☒	☐	Plants

Methods

n/a	Involved in the study	
☒	☐	ChIP-seq
☒	☐	Flow cytometry
☒	☐	MRI-based neuroimaging

Antibodies

Antibodies used	The VRC01 drug product stock concentrations were prepared at Duke and sent to the NICD; thus the two laboratories worked with identical material.
Validation	The VRC01 drug product was used as a positive control; heat inactivation did not affect the neutralization activity of the VRC01 drug product when spiked into a normal human serum sample. The VRC01 drug product was assayed against each HIV-1 Env-pseudotyped virus three times at starting concentrations of 100 µg/ml and 5 µg/ml using eight three-fold serial dilutions in duplicate. HIV-1 PVO.4 Env-pseudotyped virus was included in each assay as a positive control to confirm the integrity of the VRC01 drug product.<br>The assay has been formally validated for accuracy, sensitivity, specificity, precision, linearity, range and robustness.

Eukaryotic cell lines

Policy information about cell lines and Sex and Gender in Research

Cell line source(s)	293T/17 cells were obtained from American Type Culture Collection (Cat#CRL-11268). TZM-bl cells were obtained from the NIH AIDS Research and Reference Reagent Program (Cat#ARP-8129).
Authentication	293T/17: No additional authentication procedures were conducted except to confirm the morphology and adherence patterns of the cells under the microscope. The cells also performed as expected in virus growth assays.<br>TZM-bl: No additional authentication procedures were conducted except to confirm the morphology and adherence patterns of the cells under the microscope. Obtaining expected results in our external proficiency programme was also part of the authentication.
Mycoplasma contamination	We confirmed on a regular basis that all cell lines tested negative for mycoplasma.
Commonly misidentified lines<br>(See ICLAC register)	None used.

Clinical data

Policy information about clinical studies

All manuscripts should comply with the ICMJE guidelines for publication of clinical research and a completed CONSORT checklist must be included with all submissions.

Clinical trial registration	ClinicalTrials.gov numbers NCT02716675 and NCT02568215
Study protocol	Full trial protocols are available with the primary publication: https://www.nejm.org/doi/full/10.1056/NEJMoa2031738
Data collection	Enrollment is described in the primary publication: https://www.nejm.org/doi/full/10.1056/NEJMoa2031738 "For the AMP trials, between April 6, 2016, and October 5, 2018, a total of 2699 participants were enrolled in HVTN 704/HPTN 085, and between May 17, 2016, and September 20, 2018, a total of 1924 participants were enrolled in HVTN 703/HPTN 081." Clinical data were collected through Case Report Forms (CRFs) that are part of an electronic data capture (EDC) system or through electronic patient-reported outcome (ePRO). Laboratory data were collected at central research labs.<br>For laboratory data collection: Neutralizing antibody titers were measured in serum samples collected according to Schedule 1 - Laboratory procedures for HIV-uninfected participants (Appendix F, Study Protocol; Corey et al. NEJM 2021). Neutralizing antibodies were assayed either by the Montefiori Lab (Duke University, USA) or the Morris Lab (National Institute for Communicable Diseases, South Africa). Viral load measurements were measured in samples collected at pre-ART initiation visits according to Schedule 2 - Laboratory procedures for HIV-uninfected participants (Appendix F, Study Protocol; Corey et al. NEJM 2021). HIV diagnostics and HIV PCR viral load data were assayed by the University of Washington Virology Specialty Laboratory (Seattle, WA, USA) or the Regional Network HIV Diagnostic Lab.
Outcomes	Described in the primary publication: https://www.nejm.org/doi/full/10.1056/NEJMoa2031738. This manuscript does not report primary or secondary outcomes.

Plants

Seed stocks	Not applicable.
Novel plant genotypes	Not applicable.
Authentication	Not applicable.
